# Computational Analysis of Candidate Disease Genes and Variants for Salt-Sensitive Hypertension in Indigenous Southern Africans

**DOI:** 10.1371/journal.pone.0012989

**Published:** 2010-09-27

**Authors:** Nicki Tiffin, Ayton Meintjes, Rajkumar Ramesar, Vladimir B. Bajic, Brian Rayner

**Affiliations:** 1 Division of Nephrology and Hypertension, University of Cape Town/Groote Schuur Hospital, Cape Town, South Africa; 2 Computational Biology, Institute for Infectious Diseases and Molecular Medicine, University of Cape Town, Cape Town, South Africa; 3 Medical Research Council (MRC) Research Unit for Human Genetics, Division of Human Genetics, Institute for Infectious Diseases and Molecular Medicine, University of Cape Town, Cape Town, South Africa; 4 Computational Bioscience Research Center (CBRC), King Abdullah University of Science and Technology (KAUST), Thuwal, Kingdom of Saudi Arabia; King Abdullah University of Science and Technology, Saudi Arabia

## Abstract

Multiple factors underlie susceptibility to essential hypertension, including a significant genetic and ethnic component, and environmental effects. Blood pressure response of hypertensive individuals to salt is heterogeneous, but salt sensitivity appears more prevalent in people of indigenous African origin. The underlying genetics of salt-sensitive hypertension, however, are poorly understood. In this study, computational methods including text- and data-mining have been used to select and prioritize candidate aetiological genes for salt-sensitive hypertension. Additionally, we have compared allele frequencies and copy number variation for single nucleotide polymorphisms in candidate genes between indigenous Southern African and Caucasian populations, with the aim of identifying candidate genes with significant variability between the population groups: identifying genetic variability between population groups can exploit ethnic differences in disease prevalence to aid with prioritisation of good candidate genes. Our top-ranking candidate genes include parathyroid hormone precursor (*PTH*) and type-1angiotensin II receptor (*AGTR1*). We propose that the candidate genes identified in this study warrant further investigation as potential aetiological genes for salt-sensitive hypertension.

## Introduction

Essential hypertension is a complex disease characterised by a sustained elevation in blood pressure with no known underlying medical or biological cause. The heritability of hypertension is thought to range from 30% to 60%, with variable clinical presentation and drug response due to multiple contributory genes, genetic/ethnic heterogeneity and environmental effects [1], [2]. Genome-wide linkage, association, and candidate gene studies in patients and animal hypertension models have identified an array of associated variants for hypertension that include sodium channels, the sympathetic nervous system and adrenergic pathways, and the renin-angiotensin-aldosterone system (reviewed in [1], [3], [4]).

Although high dietary salt intake may be associated with hypertension, blood pressure response to salt in individuals is heterogeneous and most likely due to the genetic background of individuals. Thus, in some cases, hypertension appears to be salt-sensitive and reduction of dietary salt results in a significant decrease in blood pressure, and conversely salt resistance if there is little or no change. The underlying genetics of this type of hypertension, however, have not been fully elucidated [5]. Evidence to date suggests the involvement of variants of sodium channels, renal ion channels, the mineralocorticoid receptor, and related proteins affecting synthesis and levels of mineralocorticoids (reviewed in [2]). Additional proposed disease variants include those of the α-adducin gene (increasing sodium/potassium pump activity), the glucagon gene, (reducing cAMP production and impairing natriuresis), the serum- and glucocorticoid-regulated kinase (*SGK1*) gene (enhancing aldosterone–induced expression of sodium channels) [6], and genes involved in arachidonic acid metabolism [7] (reviewed in [8]). Additionally, variation in the *Cyp4A10* gene, a member of the cytochrome P450 family involved in the functional regulation of sodium channels, may cause salt-sensitive hypertension [9].

Salt-sensitive hypertension appears to be more prevalent in people of indigenous African origin, for example it was found in 73% of African-American hypertensive patients when compared with 51% of the hypertensive population and 26% of normotensive individuals [10], [11]. Of interest, it has also been shown that Caucasians secrete a water load more rapidly than indigenous Africans, perhaps due to a difference in renal water handling. This may also have an impact on occurrence of salt-sensitive hypertension in the two populations [12]. In a study from South Africa, suppressed plasma renin activity (an index of salt sensitivity) was significantly lower in both normotensive and hypertensive indigenous African patients compared to Caucasians despite comparable sodium intake. This was particularly evident in normotensives where 14.9% of Caucasians and 70% of indigenous Africans have a plasma renin activity <1.1 ng/ml/hr [13]. We have previously shown a relationship of the R563Q mutation of the *SCNN1b* gene with hypertension, but the prevalence of this SNP in the hypertensive population is 5% and <1% in normotensives and cannot account for the suppressed plasma renin activity in 70% of indigenous Africans [14].

Although it can be shown that certain risk factors and diseases vary as a function of ethnicity, it is difficult to translate this effectively into therapeutics until the underlying genotypes are better understood. The ultimate aim would be to optimise treatment based solely on genotype and regardless of ethnicity. However in the first instance, population diversity and ethnic differences in disease risk present an avenue of research towards understanding the underlying disease genetics [15].

Computational identification of most likely etiological genes can facilitate more efficient identification of genes of diagnostic, prognostic and therapeutic value by presenting strong candidates for future empirical research. The aim of this study, therefore, is to use computational approaches to prioritize and present most likely disease gene candidates for salt-sensitive hypertension, for further empirical analysis by translational researchers. In this study, we have used gene functional annotation associated with hypertension and salt-sensitivity to predict and rank novel candidates for salt-sensitive hypertension. Our analysis uses a computational approach that combines text-mining of PubMed abstracts for potentially relevant genes, and extensive mining of gene annotation data. We have then analysed Affymetrix single nucleotide polymorphism (SNP) data in a total of 126 indigenous South African individuals, spanning five distinct indigenous South African population groups, for top-scoring candidate genes from the computational analysis. We have compared allele frequencies at all SNPs assayed in indigenous Southern Africans, with those in the Caucasian population assayed in the HapMap project [16]. We have also looked for variation in the copy number of candidate genes between the different population groups. Our intention is to prioritise by computational approaches most likely candidate genes for salt-sensitive hypertension; and to prioritise further those candidates that are sufficiently different between the South African and Caucasians to potentially underlie susceptibility to salt-sensitive hypertension in indigenous Southern African populations.

## Methods

### Selection of candidate genes by Gene Ontology (GO) annotation

Data was accessed from the Ensembl database (Ensembl_mart_47) [17]. GO annotations were selected using AmiGO ([18], www.geneontology.org) to reflect a range of pathways and functions. An overview of the functions and pathways included is shown in Table 1 (1–9). The full descriptions of all GO terms used are shown in Supplementary Data File S1.

**Table 1:** Criteria for terms used to identify candidate genes.

Genes involved in regulatory pathways, and second messenger/signalling pathways, as annotated with GO terms	Genes	Score
1. Renin-angiotensin-aldosterone pathway	183	1
i.‘angiotensin’-containing GO terms		
ii. ‘renin’-containing GO terms		
iii. ‘aldosterone’-containing GO terms		
2. Renin-angiotensin-aldosterone pathway second messengers		
i. cAMP/protein kinase- associated GO terms		
ii. Calcium signalling – related GO terms		
iii. Protein Kinase A – related GO terms		
3. Adrenergic or sympathetic nervous system	1707	1
i. “adrenergic”- containing GO terms		
ii. “sympathetic”-containing GO terms		
4. Adrenergic or sympathetic nervous system second messengers		
i. GTP-binding – related GO terms		
5. Brain and atrial natriuretic peptide	17	1
i. natriuretic peptide receptor activity –related GO terms		
ii. regulation of systemic arterial blood pressure by atrial natriuretic peptide – related GO terms		
6. Brain and atrial natriuretic peptide signalling-related terms		
i. cGMP/Protein Kinase G – related GO terms		
7. Dopaminergic system	210	1
i. ‘dopamine receptor’– containing GO terms		
8. Dopaminergic system signalling-related terms		
i. ‘dopamine receptor signaling’ – related GO terms		
ii. adenylate-cyclase/dopamine receptor/G-protein – related GO terms		
iii. cAMP-dependent protein kinase–related GO terms		
iv. phospholipase C-related GO terms		
**Gene product is part of sodium pump or channel**		
9. “sodium channel”- containing GO terms	49	1
**Gene influences sodium excretion or reabsorption in the kidney**		
10. Gene names co-occurring with sodium excretion/reabsorption/kidney terms in PubMed abstracts		
i. Genes co-occurring with “sodium excretion” and “kidney” [4173 PubMed hits]	59	0.5
ii. Genes co-occurring with “sodium reabsorption” and “kidney” [1568 PubMed hits]	84	
iii. Genes co-occurring with “sodium” and “reabsorption” and “kidney” [4643 PubMed hits]	140	0.5
iv. Genes co-occurring with “sodium” and “excretion” and “kidney” [9799 PubMed hits]	99	
**Activity of gene is associated with low renin and with variable aldosterone levels**		
11. Gene names co-occurring with low renin/variable aldosterone levels in PubMed abstracts		
i. Genes co-occurring with “low renin” [997 PubMed hits]	24	1
ii. Genes co-occurring with “aldosterone levels” [1786 PubMed hits]	41	

### Selection of candidate genes by text mining of PubMed abstracts

Text-mining was used to identify gene names that most frequently occur in conjunction with a variety of salt-sensitive hypertension-related terms. These terms are described in Table 1 (10–11). The PubMed queries were made on March 24, 2008. Six queries (Table 1) retrieved in total 22,966 PubMed abstracts that were analyzed by Dragon Explorer System (DES), a licensed tool of OrionCell http://www.orioncell.org. DES uses proprietary and manually curated dictionaries of entities related to various topics and maps them to the text documents for extracting potentially relevant information from these documents. The functioning of the text-mining modules of DES is based on similar concepts as described in [19]and [20], and has been previously applied in the creation of a DDESC database of sodium channels [21] and for parts of the DDOC database [22] and DDEC databases of esophageal cancer [23]. In this study, DES is applied with the dictionary of “human genes and proteins” that contains over 300,000 variants of names, symbols, aliases, previous names and previously used symbols of genes and proteins, compiled from the literature and public databases. In the study by Sagar et al. the accuracy of DES systems to correctly identify human genes and proteins in PubMed abstracts was estimated to be with sensitivity of 81%, specificity of 96% and F-measure of 88% [21]. After gene and protein names have been identified, the respective EntrezGene IDs are determined, which eliminates naming redundancies. These genes have been used for further analysis in our study.

### Scoring and ranking of candidate genes

Gene lists were generated that fulfilled the various categories as described above and in Table 1. For each gene included, a cumulative score was assigned for every category assayed that was met by the gene. For most categories, the gene was assigned a score of one if the category was met. However for some of the terms found in PubMed abstracts, this score was divided such that a score of 0.5 was assigned if the gene co-occurred with the independent components of the given phrase. An additional score of 0.5 was subsequently only assigned if certain of these components occurred together as a complete phrase. For example a gene co-occurring with “sodium” and “reabsorption” and “kidney” will score 0.5, whereas a gene co-occurring with “sodium reabsorption” and “kidney” will score (0.5+0.5) = 1.

### Selection of SNPs by population-specific allele frequencies

This study was reviewed by the Ethics Committee of the University of Cape Town and received research ethics approval (REC REF 305/2009: “Genome Wide Microarray Analysis of Southern African Human Populations”). For five self-identified ethnic/linguistic indigenous South African population groups, allele frequencies in the genetic material from a total of 126 individuals were analysed using the Affymetrix GenomeWideSNP 6.0 Array (Homo sapiens, Genome assembly: NCBI Build 36, UCSC hg18, covering 906 600 SNPs and more than 946 000 probes for the detection of copy number variation). All individuals were collected as unrelated and confirmed that their parents and grandparents were from the same ethnic groups. DNA was prepared from peripheral blood by standard phenol–chloroform procedures and shipped to Affymetrix (http://www.affymetrix.com) for genotyping (full data under preparation for publication). Genotypes were called using the Birdseed algorithm distributed with Affymetrix Power Tools [24]. Quality of CEL files was assessed with the Dynamic Model (DM) algorithm, and only individuals (CEL files) with QC>90 were included in downstream genotyping calling. Population groups included are, with number of individuals in parentheses, Khoisan (22), Xhosa (34), Hererro (25), Setswana (25) and Zulu (20). For each candidate gene, all SNPs analysed using the Affymetrix array were selected (a total of 1079 SNPs, full data in supplementary data file S2), and the allele frequencies calculated across the South African populations. All of these South African allele frequencies for each South African population group were then compared to the allele frequencies for these SNPs as reported for Caucasians by the HapMap project [16]. Information regarding the nature of each SNP was downloaded from the Ensembl database (www.ensembl.org, [25]) wherever such data was available.

### Analysis of Copy Number Variation

Copy number analysis was performed with the Birdsuite package (version 1.5.2) [26], which uses hybridisation intensities of both SNP and CN probes to provide greater coverage and enable the detection of novel as well as known copy-number variations. Default settings, as described in [26] were used, with the exception that copy number models were not limited to known variants. Reference CEL files for the HapMap CEU population were processed using the same configuration. In addition to the previously mentioned samples filtered due to low quality, two Zulu samples were identified as having high copy number variance and removed. For each gene and its flanking sequence, a heatmap was generated to indicate copy number of probes assayed.

### Statistical Analysis

The significance level of differences in allele frequencies for the same SNPs between the different populations was calculated using the Fishers Exact Test, using Python scripting with the RPy module (http://rpy.sourceforge.net/) and R statistical software [27].

## Results

### Identification of candidate genes

In total, 2057 unique genes were included across all gene lists, and were used as the primary set of candidate genes. Each of these was then assayed for the various characteristics (see Table 1), by both text-mining and GO annotation, and assigned a cumulative score, shown in Table 2. The genes were ranked by this score, and the top scoring candidates prioritised as most likely candidate disease genes (scoring for all candidates shown in Supplementary Data File S3). The top scoring candidates were curated to exclude any spurious results. The top ranking genes were *PTH* - Parathyroid hormone precursor and *AGTR1* - Type-1 angiotensin II receptor. A selection of additional likely candidates was made from the genes ranked in the top twenty positions, as shown in Table 3 (full set of top candidates shown in Supplementary Data File S4).

**Table 2:** Scoring of candidates selected for further analysis (full data shown in file S2).

Gene Symbol	adrenergic_sympathetic	atrial_natriuretic_peptide	dopamine_receptor	renin_angiotensin_aldosterone	sodium_transport	sodium_reabsorption_kidney	(sodium_reabsorption)_kidney	(sodium_excretion)_kidney	sodium_excretion_kidney	low_renin	aldosterone_levels	Total
*PTH*	1	-	1	-	-	0.5	0.5	0.5	0.5	1	1	**6**
*AGTR1*	1	-	-	1	-	0.5	0.5	0.5	0.5	1	1	**6**
*EDNRA*	1	-	1	-	-	0.5	0.5	0.5	0.5	1	-	**5**
*AGT* *	-	-	-	1	-	0.5	0.5	0.5	0.5	1	1	**5**
*REN*	-	-	-	1	-	0.5	0.5	0.5	0.5	1	1	**5**
*HCN4*	1	-	1	1	1	-	-	-	-	-	-	**4**
*EDNRB* *	1	-	1	-	-	0.5	0.5	0.5	0.5	-	-	**4**
*ANG*	-	-	1	-	-	0.5	0.5	0.5	0.5	-	1	**4**
*NPPA* *	-	-	-	-	-	0.5	0.5	0.5	0.5	1	1	**4**
*INS*	-	-	-	-	-	0.5	0.5	0.5	0.5	1	1	**4**
*ACE* *	-	-	-	-	-	0.5	0.5	0.5	0.5	1	1	**4**
*EDN1*	-	-	-	-	-	0.5	0.5	0.5	0.5	1	1	**4**

Genes with appropriate second-messenger annotations are included in each category.

*Genes also annotated with hypertension-related GO terms.

**Table 3:** Candidate genes selected for further analysis.

Gene Symbol	Ensembl ID	Description
*PTH*	ENSG00000152266	Parathyroid hormone precursor (Parathyrin) (PTH) (Parathormone)
*AGTR1*	ENSG00000144891	Type-1 angiotensin II receptor (AT1) (AT1AR) (AT1BR).
*EDNRA*	ENSG00000151617	Endothelin-1 receptor precursor (Endothelin A receptor) (ET-A) (hET- AR) (ETA-R).
*AGT*	ENSG00000135744	Angiotensinogen precursor (Serpin A8) [Contains: Angiotensin-1 (Angiotensin I) (Ang I); Angiotensin-2 (Angiotensin II) (Ang II); Angiotensin-3 (Angiotensin III) (Ang III) (Des-Asp[1]-angiotensin II)].
*REN*	ENSG00000143839	Renin precursor (EC 3.4.23.15) (Angiotensinogenase)
*HCN4*	ENSG00000138622	Potassium/sodium hyperpolarization-activated cyclic nucleotide-gated channel 4
*EDNRB*	ENSG00000136160	Endothelin B receptor precursor (ET-B) (Endothelin receptor Non- selective type).
*HCN2*	ENSG00000099822	Potassium/sodium hyperpolarization-activated cyclic nucleotide-gated channel 2 (Brain cyclic nucleotide-gated channel 2) (BCNG-2).
*ANG*	ENSG00000214274	Angiogenin precursor (EC 3.1.27.-) (Ribonuclease 5) (RNase 5).
*NPPA*	ENSG00000175206	Atrial natriuretic factor precursor (ANF) (Atrial natriuretic peptide) (ANP) (Prepronatriodilatin) (CDD-ANF) [Contains: Cardiodilatin-related peptide (CDP)].
*INS*	ENSG00000129965	Insulin precursor [Contains: Insulin B chain; Insulin A chain]
*ACE*	ENSG00000159640	Angiotensin-converting enzyme
*EDN1*	ENSG00000078401	Endothelin-1 precursor (Preproendothelin-1) (PPET1) [Contains: Endothelin-1 (ET-1); Big endothelin-1].

### Comparative analysis of SNP frequencies in candidate genes, between populations

**Figure 1:**
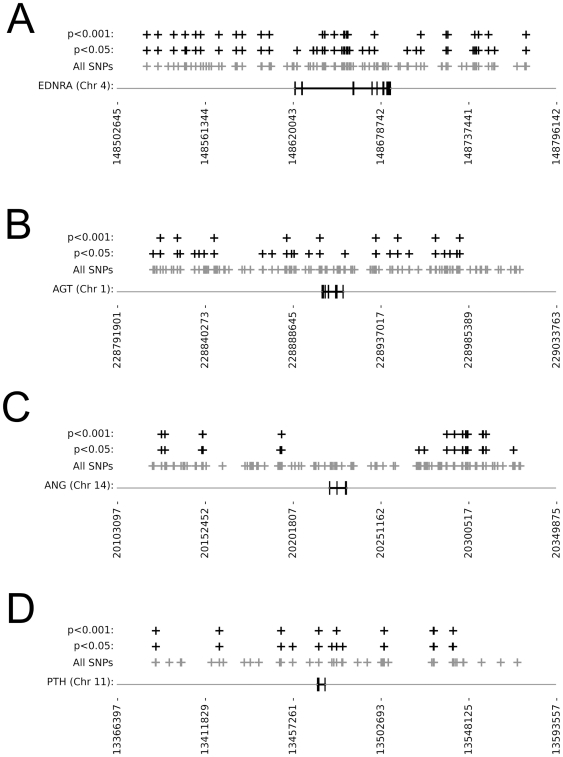
Examples of distribution of SNPs with significantly different allele frequencies when comparing indigenous Southern African and Caucasian populations (all genes shown in supplementary data file S5). Gene exon structure (bold vertical bars), chromosome and base pair position (horizontal axis) are shown. Each black cross represents a SNP with allele frequencies that differ between Caucasian and South African populations at the level of significance indicated, and the grey crosses represent all SNPs analysed. A. *EDNRA* (many SNPs within the gene) B. *AGT* (a few SNPs within the gene, most SNPs in flanking regions); C. *ANG* (no SNPs within the gene); D. *PTH* (few SNPs assayed within the gene).

A total of 1079 SNPs were selected from the Affymetrix data as associated with the prioritised candidate genes, falling either within the genes or within 100 000 bp either upstream or downstream of the gene (Supplementary Data File S2) (the *SCNN1b* gene was not included in this analysis as it was not one of the prioritised genes). Of these, 303 had allele frequencies that were significantly different between Caucasian and all indigenous South African populations at a threshold of p<0.05. There were 159 SNPs that had allele frequencies that were significantly different at a threshold of p<0.001. The categories and numbers of SNPs selected are shown in Table 4. Additionally, the numbers of SNPs falling within the coding region of the gene and not the flanking sequence are shown. These were distributed across the candidate genes as shown in Table 5, and results for a selection of genes are shown in Figure 1 (SNP distribution in all genes and flanking sequences are shown in supplementary file S5). The distribution of SNPs within the genes and excluded from the flanking sequence are also shown.

**Table 4:** The categories and numbers of all SNPs selected.

	With 100000 bp flanking sequence		Without flanking sequence	
SNP Type	P<0.05	P<0.001	P<0.05	P<0.001
3′ Downstream	18	13	1	1
3′ UTR	5	3	1	0
5′ Upstream	14	6	0	0
Coding synonymous	3	3	2	2
Coding nonsynonymous	1*	0	0	0
Intronic	104	43	43	22
Undefined	158	91	0	0

*This falls in a neighbouring gene, and not the candidate gene of interest.

**Table 5:** The numbers of SNPs selected for each candidate gene.

		Flanking sequence included			Gene sequence only		
Gene Name	Gene Length (bp)	Total SNPs Assayed	SNPs selected p<0.05	SNPs selected p<0.001	Total SNPs Assayed	SNPs selected p<0.05	SNPs selected p<0.001
HCN4	47242	49	7	2	8	0	0
HCN2	27264	17	4	3	1	1	1
INS	32100	46	9	3	9	1	1
NPPA	2633	59	11	4	3	0	0
EDNRB	24126	82	21	6	16	4	1
PTH	3965	48	14	9	1	1	1
AGT	11667	131	27	9	9	0	0
ACE	44769	45	16	9	19	4	2
ANG	9571	130	22	15	8	0	0
REN	11517	110	35	16	15	2	2
EDN1	6800	124	38	24	9	5	3
EDNRA	63980	101	42	27	42	18	9
AGTR1	45123	137	57	32	25	11	5

### Analysis of Copy Number Variation

Variation in copy number of individual probes is evident, as shown on the heatmaps generated; however there was no evidence of a change in copy number at the gene level for any of the candidate genes analysed (supplementary data file S6).

## Discussion

The high ranking genes in the candidate gene list included several previously identified causative genes for hypertension. The top scoring *AGTR1* gene has been extensively implicated in controlling blood pressure and volume in the cardiovascular system and pharmacologic agents that interrupt the action of angiotensin II action by antagonizing the AGTR1 receptor are highly successful in the treatment of angiotensin II-dependent hypertension [28]. *AGT*, *AGTR1*, *ACE* and *REN* are all members of the renin-angiotensin-aldosterone pathway (as shown in www.pharmgkb.org, the ACE inhibitor pathway). ACE inhibitors and AGTR1-blocking drugs are already extensively exploited as therapy for hypertension. Given that these genes are well annotated and form the foundation of the characteristics used to select new candidates, it is unsurprising that they are highly scored in the analysis and act as an internal control showing that the selective process is accurate. The same is true of *NPPA*- Atrial natriuretic peptide [29], although interestingly this gene was not selected by atrial natriuretic peptide-specific GO annotation, but rather by association in PubMed abstract with terms including sodium reabsorption/excretion, low renin and aldosterone levels.

Despite our previous description of the association of hypertension with the R563Q mutation, the *SCNN1b* gene was not identified as a good candidate gene. It only fulfilled one of the characteristics that we used to prioritise candidates (“sodium transport”), and therefore was not assigned a high priority as a candidate gene. The focus of this computational analysis is to synthesise various sources of information to highlight less obvious good candidates, such as *PTH*, rather than selecting more obvious candidates such as *SCNN1b* based only on a one-dimensional analysis of the direct link between gene and disease. The limitations of detecting genetic associations with hypertension must also be noted, whereby detection of common variants with small effects is more likely than detection of a rare variant dominant effect like the R563Q mutation. [30].

*PTH* is also top-scoring alongside *AGTR1*. It has a less defined role in essential hypertension, which makes it an interesting novel candidate for salt-sensitive hypertension. Several lines of research support a potential role for PTH in the pathogenesis of salt-sensitive hypertension. It has been proposed that the level of PTH may be raised as a secondary response to hypertension [31], [32], as a response to dysregulated sodium excretion from the kidney. It has, however, also been shown that administration of PTH to healthy volunteers under normal glycaemic conditions, in the presence of controlled circulating insulin levels, results in a significant and consistent increase in blood pressure and concurrently in ionised calcium concentration. The increase in blood pressure was strongly correlated to changes in calcium ion concentration [33]. Similarly, hypercalcaemia and hypertension were observed in response to administration of supraphysiological levels of PTH [[34]. Further observations have been made previously that in uraemic patients with secondary hyperparathyroidism, in patients with secondary hyperparathyroidism, and in genetically pre-hypertensive patients a positive correlation is found between blood pressure and PTH/Calcium ion concentrations [35], [36], [37]. In humans salt-induced increases in blood pressure have been linked to increases in intracellular free calcium [38]. The calcium binding to the Ca-sensing receptor expressed in the thick ascending limb inhibits the Na,K, 2CL cotransporter, which decreases both sodium reabsorption and thus influences blood pressure [39]. Finally, the prevalence of hypertension is known to be high in patients with primary hyperparathyroidism [40].

Additionally, PTH levels are found to be elevated in African American individuals when compared to those of Caucasian descent [41], which further supports a role for *PTH* as an underlying causative gene for salt-sensitive hypertension in indigenous South African patients. However it is possible that this is an indirect association as these elevated levels may be the result of vitamin D insufficiency, due to less sun-stimulated vitamin D synthesis in dark-skinned people [41], [42].

Finally, in a prospective study, Taylor et al. examined the association between plasma intact PTH levels and the risk of incident hypertension in 481 men without baseline hypertension, and suggested that plasma levels of intact PTH (even within ranges considered normal) are positively and independently associated with a higher risk of incident hypertension. These authors proposed that PTH could potentially serve as a novel target for the prevention of hypertension [43], supporting its ranking as a primary candidate gene in our study. This may also have implications for therapy as these patients could potentially respond better to calcium channel blockers.

Of the additional high-scoring candidates, some have been previously directly associated with hypertension and salt-sensitivity. Candidates *EDNRA*, *EDN1* and *EDNRB* function together in a regulatory pathway with endothelin-converting enzyme *ECE1*, which has been implicated in essential hypertension. Additionally, Rothermund et al. proposed a role for *EDNRA* and *EDNRB* in salt-sensitive hypertension [44]. Our study suggests that these genes are good candidates and should be revisited for a potential role in salt-sensitive hypertension. The association between the insulin gene, *INS*, and hypertension has been well documented. There is extensive cross-talk between insulin and angiotensin II regulating the metabolic and circulatory systems, with effects at both the extracellular level whereby ACE controls angiotensin II synthesis and interferes with insulin signalling through angiotensin II regulation and accumulation of bradykinin; and at the intracellular level whereby signal transduction pathways are affected (reviewed in [45], [46]). Clinically, the association between insulin resistance and pre-hypertension has been demonstrated [47], and it has also been proposed that insulin promotes renal sodium retention ([48] and references therein). Therefore insulin is a good candidate for salt-sensitive hypertension, as it has been implicated in both hypertension and sodium retention.

Other high-scoring candidates have not been shown previously to have a direct role in hypertension, although they do have roles in vascular and heart physiology, and have been associated with other diseases. *HCN2* and *HCN4* are hyperpolarisation-activated cation channels of the *HCN* gene family, and contribute to spontaneous rhythmic activity in both heart and brain. Expression of HCN4 is predominantly in the heart, as well as the thalamus and the developing central nervous system. Mutations in *HCN4* have been associated with the cardiac disease, sinus bradycardia (OMIM #163800). The gene product of *ANG*, the angiogenin gene, is an inducer of neovascularization, and the RNase activity of ANG is important for its angiogenic activity. It is induced by hypoxia to elicit angiogenesis and is expressed in motor neurons; mutations in *ANG* are associated with the neurodegenerative disease amyotrophic lateral sclerosis in some populations (OMIM # 611895).

The computational approach we have used has selected both hypertension-associated and novel candidate genes for salt-sensitive hypertension. We have used a variety of gene characteristics and functional annotations to select most likely candidates for this disease. Although text- and data-mining do rely on existing information to inform the results, such computational approaches are able to synthesise huge volumes of existing data both through the biomedical literature, and gene annotation in genomic databases. The systematic analysis and categorisation of such volumes of existing data allow novel connections and inferences to be made computationally. The scoring system is based on the premise that a candidate gene selected by multiple sources of evidence is a better candidate than one selected by fewer lines of evidence (as previously demonstrated in [49], [50], [51]), and therefore allows objective weighting of the value and extent of such inferences. In general, the ability of our method to identify a number of genes already implicated in hypertension indicates that the methodology we have used is sound, and that the characteristics by which we have prioritised candidates are appropriate. Our method, however, has also highlighted novel candidates for which disparate existing knowledge recorded in the biomedical literature and in functional gene annotation has not previously been collated to support a role in salt-sensitive hypertension.

The sample set of indigenous South Africans is relatively small, and because of this, the information about allele frequency and copy number variation is presented here as preliminary data. Because of the high incidence of hypertension in indigenous South Africans (for example up to 25% in urban Zulu individuals [52]), of which up to 50% show suppressed plasma renin activity, an indirect measure of salt sensitivity [13], it is however likely that a genetic predisposition to this disease is represented in a fair proportion of the population, and that the evidence for this can be found in our sample set when compared to Caucasians. Such information can be invaluable to the researcher designing studies to identify the underlying genetics of salt-sensitive hypertension: as with all candidate disease gene studies, we present here a prioritisation of alleles for further investigation – only further empirical analysis of appropriately sized sample sets would be able to confirm the association of particular alleles with salt-sensitive hypertension.

It is difficult to make a direct comparison between the numbers, positions and types of SNPs identified for each candidate gene, as the gene sizes and total number of SNPs are so varied. The distribution of SNPs within candidate genes can be described in several approximated categories, and examples are shown in Figure 1. In some genes, there are no selected SNPs falling within the gene itself despite many SNPs being assayed in those regions, and those in flanking sequences are fairly distant from the gene – these are *AGT*, *ANG*, *HCN4* and *NPPA*. In some, a few SNPs are selected within and close to the gene, although many more SNPs were assayed than selected in these regions – these are *REN*, *ACE*, *EDNRB* and *INS*. We propose that these candidates that show fewer differences in allele frequency are less likely to underlie the salt-sensitive hypertension that is seen in indigenous Southern African populations. In a few candidates, many of the assayed SNPs were selected falling within or close to the gene – these are *EDNRA*, *EDN1* and *AGTR1*. Finally, in a few of the candidates, few SNPs were assayed within or close to the gene, although these were generally selected as having significantly different alleles between the populations – these are *HCN2*, and the primary candidate *PTH*. The two latter categories contain candidates that appear to have substantial differences in allele frequencies between Caucasian and the indigenous Southern African populations, and as such are more likely to contain variants that are responsible for the salt-sensitive hypertension that is prevalent in the indigenous Southern African population. Our analysis of copy number variation did not suggest that the candidate genes investigated here have any alteration in copy number at the gene level in either of the population groups. Copy number variation of these genes is therefore unlikely to underlie the salt-sensitive hypertension that is prevalent in the indigenous Southern African populations (although it is interesting to note that the heatmaps generated for the genes *AGTR1* and *INS* do show evidence of population-specific variation at sites within the genes - see supplementary data file S6).

The selection of top-scoring candidates in this study is inevitably affected by some inherent bias: appropriate genes that have more extensive annotations are more likely to be selected in the study than those that have not yet been analysed, so it is more difficult to select entirely novel candidates. However, we have aimed to combine existing knowledge about hypertension and salt sensitivity to draw new conclusions about candidate genes for salt-sensitive hypertension. Computational analysis thus allows the synthesis of existing information to make novel predictions.

Due to the population-specific nature of salt-sensitive hypertension, we propose that the SNPs in the prioritised candidate genes that show significantly different allele frequencies between Caucasian and indigenous African populations provide good targets for further clinical and empirical research. We provide here a list of good candidate genes and alleles related to salt-sensitive hypertension that have been selected and prioritised by computational methods. These prioritized genes may have a significant contribution to the occurrence of salt-sensitive hypertension, and thus warrant further investigation by translational researchers.

## Supporting Information

Data File S1 GO Terms used for the Selection of Candidate Genes for Salt-Sensitive Hypertension.
(0.03 MB PDF)

Data File S2 All SNPs assayed.
(0.08 MB PDF)

Data File S3 Final Scoring matrix for all genes.
(0.08 MB PDF)

Data File S4 Ensembl IDs, HUGO symbols and descriptions of all top candidates.
(0.01 MB PDF)

Data File S5 Distribution of SNPs with significantly different allele frequencies when comparing Black South African and Caucasian populations. Each black cross represents a SNP with allele frequencies that differ at the indicated significance, between Caucasian and South African populations. Grey crosses indicate all SNPs assayed. Gene name, chromosome and position are shown along the horizontal axis; Gene exons are shown as vertical black bars.
(1.13 MB PDF)

Data File S6 Heatmaps showing copy number variation analysis for candidate genes. Red shading shows a deletion at that position, blue shading shows a copy number gain at that position. HUGO gene symbol is shown. Chrm = chromosome. Green lines denote the gene start and end. Horizontal axis shows position on the chromosome. Vertical axis shows the individuals from each population: CEU = HapMap Central European population, XHS = Xhosa population, HER = Hererro population, STS = Setswana population, ZUL = Zulu population, KHS = Khoisan population.
(3.53 MB PDF)
