# Supplementary material for: Computational Analysis of Candidate Disease Genes and Variants for Salt-Sensitive Hypertension in Indigenous Southern Africans
Source: PLoS One. 2010 Sep 27;5(9):e12989. doi: 10.1371/journal.pone.0012989 (PMC2946338; doi:10.1371/journal.pone.0012989)
Supplement: Data File S1 — GO Terms used for the Selection of Candidate Genes for Salt-Sensitive Hypertension. (0.03 MB PDF) [file pone.0012989.s001.pdf]

Supplementary File S1: GO Terms used for the Selection of Candidate Genes for Salt-Sensitive Hypertension

1. Gene located in metabolic pathways

Use of Gene Ontology (GO) terms (www.geneontology.org) to select genes associated with the pathways.

i. Renin-angiotensin-aldosterone

GO terms:

i. 'renin'-containing terms
- brain renin-angiotensin system ; GO:0002035
- gamma-renin activity ; GO:0004253
- regulation of blood vessel size by renin-angiotensin ; GO:0002034
- regulation of systemic arterial blood pressure by circulatory renin-angiotensin ; GO:0001991
- regulation of systemic arterial blood pressure by local renal renin-angiotensin ; GO:0003086
- regulation of systemic arterial blood pressure by renin-angiotensin ; GO:0003081
- renal response to blood flow during renin-angiotensin regulation of systemic arterial blood pressure ; GO:0001999
- renin activity ; GO:0004195
- renin secretion into blood stream ; GO:0002001
- renin-angiotensin regulation of aldosterone production ; GO:0002018

ii. 'angiotensin'-containing terms
- angiotensin catabolic process in blood ; GO:0002005
- angiotensin maturation ; GO:0002003
- angiotensin mediated vasoconstriction involved in regulation of systemic arterial blood pressure ; GO:0001998
- angiotensin receptor activity ; GO:0001595
- angiotensin receptor binding ; GO:0031701
- angiotensin type I receptor activity ; GO:0001596
- angiotensin type II receptor activity ; GO:0004945
- angiotensin-converting enzyme inhibitor activity ; GO:0042031
- negative regulation of renal output by angiotensin ; GO:0003083
- positive regulation of diuresis by angiotensin ; GO:0003102
- positive regulation of natriuresis by angiotensin ; GO:0003107
- positive regulation of renal output by angiotensin ; GO:0003082
- regulation of angiotensin levels in blood ; GO:0002002
- regulation of angiotensin metabolic process ; GO:0060177
- regulation of glomerular filtration by angiotensin ; GO:0003106
- regulation of renal output by angiotensin ; GO:0002019
- type 1 angiotensin receptor binding ; GO:0031702
- type 2 angiotensin receptor binding ; GO:0031703

- vasodilation by angiotensin involved in regulation of systemic arterial blood pressure ; GO:0002033

iii. 'aldosterone'-containing terms:

- aldosterone biosynthetic process ; GO:0032342
- aldosterone catabolic process ; GO:0032343
- aldosterone metabolic process ; GO:0032341
- negative regulation of aldosterone biosynthetic process ; GO:0032348
- negative regulation of aldosterone metabolic process ; GO:0032345
- positive regulation of aldosterone biosynthetic process ; GO:0032349
- positive regulation of aldosterone metabolic process ; GO:0032346
- regulation of aldosterone biosynthetic process ; GO:0032347
- regulation of aldosterone metabolic process ; GO:0032344
- renal regulation of blood volume by aldosterone ; GO:0002017

ii. Adrenergic or sympathetic nervous system

GO terms:

i. "adrenergic"- containing terms:

- adrenergic receptor binding ; GO:0031690
- alpha-1A adrenergic receptor binding ; GO:0031691
- alpha-1B adrenergic receptor binding ; GO:0031692
- alpha-1D adrenergic receptor binding ; GO:0031693
- alpha-2A adrenergic receptor binding ; GO:0031694
- alpha-2B adrenergic receptor binding ; GO:0031695
- alpha-2C adrenergic receptor binding ; GO:0031696
- alpha-adrenergic receptor activity ; GO:0004936
- alpha1-adrenergic receptor activity ; GO:0004937
- alpha2-adrenergic receptor activity ; GO:0004938
- beta-1 adrenergic receptor binding ; GO:0031697
- beta-2 adrenergic receptor binding ; GO:0031698
- beta-3 adrenergic receptor binding ; GO:0031699
- beta-adrenergic receptor activity ; GO:0004939
- beta-adrenergic receptor kinase activity ; GO:0047696
- beta1-adrenergic receptor activity ; GO:0004940
- beta2-adrenergic receptor activity ; GO:0004941
- beta3-adrenergic receptor activity ; GO:0015052

ii. "sympathetic"-containing terms:
- sympathetic nervous system development ; GO:0048485

iii. Brain and atrial natriuretic peptide

GO terms:

- natriuretic peptide receptor activity ; GO:0016941

- regulation of systemic arterial blood pressure by atrial natriuretic peptide ; GO:0003050

iv. Dopaminergic system

D1-like dopamine receptors (D1 and D5):
- signalling mediated by heterotrimeric G proteins: Galphas, Galphaolf
- causes sequential activation of adenylate cyclase, cyclic AMP-dependent protein kinase, and DARPP-32.
- D1 also signals via Phospholipase C-dependent and cAMP- INdependent intracellular Ca+ mobilisation

D2-like receptor signalling (D2, D3, D4):
- mediated by the heterotrimeric G proteins Galphai and Galphao
- these regulator some effectors via Galpha subunit e.g. adenylate cyclase
- regulate many more effectors such as ion channels, phospholipases, protein kinases and receptor tyrosine kinases by liberated Gbetagamma subunits.

Other protein:protein interactions e.g. receptor oligomerisation, receptor interactions with scaffolding and signal-switching proteins are critical for regulation of dopamine receptor signalling.

GO terms:

i. List of GO annotations with ‘dopamine receptor’ annotations:
- D1 dopamine receptor binding ; GO:0031748
- D2 dopamine receptor binding ; GO:0031749
- D3 dopamine receptor binding ; GO:0031750
- D4 dopamine receptor binding ; GO:0031751
- D5 dopamine receptor binding ; GO:0031752
- dopamine D1 receptor activity ; GO:0001590
- dopamine D2 receptor activity ; GO:0001670
- dopamine D3 receptor activity ; GO:0001592
- dopamine D4 receptor activity ; GO:0001593
- dopamine D5 receptor activity ; GO:0001589
- dopamine receptor activity ; GO:0004952
- dopamine receptor binding ; GO:0050780

<u>2. Gene located in second messenger or signalling pathways of above systems</u>

Compile list of all genes with the following annotations:

i. Renin-angiotensin-aldosterone

.

i. cAMP/protein kinase- associated GO terms are described (as for the dopaminergic system, repeated here) as well as one cAMP signalling-associated GO term.

- cAMP-mediated signaling ; GO:0019933
- G-protein signaling, coupled to cAMP nucleotide second messenger ; GO:0007188

- cAMP-dependent protein kinase activity ; GO:0004691
- cAMP-dependent protein kinase complex ; GO:0005952
- cAMP-dependent protein kinase inhibitor activity ; GO:0004862
- cAMP-dependent protein kinase regulator activity ; GO:0008603

ii. Calcium signalling – related GO terms:

- calcium-mediated signaling ; GO:0019722
- negative regulation of calcium-mediated signaling ; GO:0050849
- positive regulation of calcium-mediated signaling ; GO:0050850
- regulation of calcium-mediated signaling ; GO:0050848

iii. Protein Kinase A – related GO terms
- activation of protein kinase A activity ; GO:0034199
- protein kinase A binding ; GO:0051018
- protein kinase A catalytic subunit binding ; GO:0034236
- protein kinase A regulatory subunit binding ; GO:0034237

ii. Adrenergic or sympathetic nervous system

i. List of GTP-binding – associated genes, using GO
- GTP binding ; GO:0005525
- G-protein coupled receptor kinase activity ; GO:0004703
- G-protein coupled receptor protein signaling pathway ; GO:0007186

iii. Atrial natriuretic peptide signalling cascade:

i. List of cGMP/Protein Kinase G – associated terms, using GO annotation

- cGMP-dependent protein kinase activity ; GO:0004692
- -protein coupled receptor kinase activity ; GO:0004703
- G-protein signaling, coupled to S1P second messenger (sphingosine kinase activating) ; GO:0001789
- GTP-dependent protein kinase activity ; GO:0034211
- cGMP-mediated signaling ; GO:0019934
- G-protein signaling, coupled to cGMP nucleotide second messenger ; GO:0007199
- intracellular cGMP activated cation channel activity ; GO:0005223

iv. Dopaminergic system:

i. List of GO annotations with ‘dopamine receptor signaling’ annotations:
- dopamine receptor signaling pathway ; GO:0007212
- dopamine receptor, adenylate cyclase activating pathway ; GO:0007191
- dopamine receptor, adenylate cyclase inhibiting pathway ; GO:0007195
- dopamine receptor, phospholipase C activating pathway ; GO:0060158
- negative regulation of dopamine receptor signaling pathway ; GO:0060160
- positive regulation of dopamine receptor signaling pathway ; GO:0060161
- regulation of dopamine receptor signaling pathway ; GO:0060159

ii. List of adenylate-cyclase/dopamine receptor/G-protein terms:

- dopamine receptor, adenylate cyclase activating pathway ; GO:0007191
- dopamine receptor, adenylate cyclase inhibiting pathway ; GO:0007195
- G-protein signaling, adenylate cyclase activating pathway ; GO:0007189
- G-protein signaling, adenylate cyclase inhibiting pathway ; GO:0007193
- negative regulation of adenylate cyclase activity ; GO:0007194
- positive regulation of adenylate cyclase activity ; GO:0045762
- regulation of adenylate cyclase activity ; GO:0045761

iii. List of cAMP-dependent protein kinase-related GO terms:

- cAMP-dependent protein kinase activity ; GO:0004691
- cAMP-dependent protein kinase complex ; GO:0005952
- cAMP-dependent protein kinase inhibitor activity ; GO:0004862
- cAMP-dependent protein kinase regulator activity ; GO:0008603

iv. List of phospholipase C-related GO terms:

- activation of phospholipase C activity ; GO:0007202
- dopamine receptor, phospholipase C activating pathway ; GO:0060158
- inhibition of phospholipase C activity ; GO:0030845
- negative regulation of dopamine receptor, phospholipase C activating pathway ; GO:0060162
- phospholipase C activity ; GO:0004629

3. Gene part of sodium pump or channel

"sodium channel": 39 genes

amiloride-sensitive sodium channel activity ; GO:0015280.	molecular function
clustering of voltage-gated sodium channels ; GO:0045162. The process by which voltage gated sodium channels become localized together in high densities. In animals, nodes of Ranvier differ dramatically from internodal axonal regions in very high densities of voltage-dependent sodium (Nav) channels responsible for the rapid, inward ionic currents that produce membrane depolarization.	biological process
intracellular sodium activated potassium channel activity ; GO:0005228. Catalysis of the transmembrane transfer of potassium by a channel that opens in response to stimulus by a sodium ion or ions. Transport by a channel involves catalysis of facilitated diffusion of a solute (by an energy-independent process) involving passage through a transmembrane aqueous pore or channel, without evidence for a carrier-mediated mechanism. Sodium activated potassium channels have distinctive properties, including a large single channel conductance, subconductance states, and a block of single channel currents at positive potentials, similar to inward rectification.	molecular function
sodium channel activity ; GO:0005272. Catalysis of facilitated diffusion of a sodium ion (by an energy-independent process) involving passage through a	molecular function

transmembrane aqueous pore or channel without evidence for a carrier-mediated mechanism.

sodium channel auxiliary protein activity ; GO:0016974.	molecular function
sodium channel inhibitor activity ; GO:0019871.	molecular function
sodium channel regulator activity ; GO:0017080.	molecular function
voltage-gated sodium channel activity ; GO:0005248. Catalysis of the transmembrane transfer of a sodium ion by a voltage-gated channel.	molecular function
voltage-gated sodium channel complex ; GO:0001518. A sodium channel in a cell membrane whose opening is governed by the membrane potential.	cellular component

"Sodium Pump": 11 genes

sodium:potassium-exchanging ATPase activity ; GO:0005391. Catalysis of the transfer of a solute or solutes from one side of a membrane to the other according to the reaction: ATP + H2O + Na+(in) + K+(out) = ADP + phosphate + Na+(out) + K+(in).	molecular function
sodium:potassium-exchanging ATPase complex ; GO:0005890. Sodium:potassium-exchanging ATPases are tetrameric proteins, consisting of two large alpha subunits and two smaller beta subunits. The alpha subunits bear the active site and penetrate the membrane, while the beta subunits carry oligosaccharide groups and face the cell exterior.	cellular component
