# Supplementary material for: Computational Analysis of Candidate Disease Genes and Variants for Salt-Sensitive Hypertension in Indigenous Southern Africans
Source: PLoS One. 2010 Sep 27;5(9):e12989. doi: 10.1371/journal.pone.0012989 (PMC2946338; doi:10.1371/journal.pone.0012989)
Supplement: Data File S2 — All SNPs assayed. (0.08 MB PDF) [file pone.0012989.s002.pdf]

Supplementary data file S2: All SNPs assayed

Gene Symbol	dbSNP ID	Affymetrix ID	Allele1	Allele2	Chromosome	Gene_start	Gene_end	SNP position
ACE	rs1029765	SNP_A-1866325	A	G	17	58808166	59052935	58813016
ACE	rs17760841	SNP_A-8562735	C	T	17	58808166	59052935	58826552
ACE	rs2270133	SNP_A-1905032	C	A	17	58808166	59052935	58827057
ACE	rs16946910	SNP_A-2130002	A	T	17	58808166	59052935	58833422
ACE	rs7211831	SNP_A-8671801	C	G	17	58808166	59052935	58838237
ACE	rs2286571	SNP_A-8300574	T	G	17	58808166	59052935	58871952
ACE	rs4968775	SNP_A-4273894	C	G	17	58808166	59052935	58872498
ACE	rs9896864	SNP_A-2292157	A	G	17	58808166	59052935	58890040
ACE	rs6504162	SNP_A-4254119	C	T	17	58808166	59052935	58891134
ACE	rs9905945	SNP_A-1881944	T	C	17	58808166	59052935	58892788
ACE	rs9912458	SNP_A-8321661	T	C	17	58808166	59052935	58903970
ACE	rs4293	SNP_A-2257238	A	G	17	58808166	59052935	58909398
ACE	rs4297	SNP_A-8509652	C	G	17	58808166	59052935	58910286
ACE	rs12709426	SNP_A-2245620	G	A	17	58808166	59052935	58915488
ACE	rs4324	SNP_A-8557538	G	A	17	58808166	59052935	58916903
ACE	rs4325	SNP_A-8560002	C	A	17	58808166	59052935	58916932
ACE	rs4329	SNP_A-8527087	G	A	17	58808166	59052935	58917190
ACE	rs12709430	SNP_A-2068378	A	G	17	58808166	59052935	58918801
ACE	rs4343	SNP_A-8680186	G	A	17	58808166	59052935	58919763
ACE	rs13306091	SNP_A-8689846	C	T	17	58808166	59052935	58920067
ACE	rs12709435	SNP_A-4231379	A	G	17	58808166	59052935	58922752
ACE	rs4349	SNP_A-8293739	T	C	17	58808166	59052935	58922876
ACE	rs4351	SNP_A-4214465	A	G	17	58808166	59052935	58923464
ACE	rs4351	AFFX-SNP_3894260	A	G	17	58808166	59052935	58923464
ACE	rs8075924	SNP_A-8610297	C	T	17	58808166	59052935	58936624
ACE	rs4267385	SNP_A-2091532	C	T	17	58808166	59052935	58937488
ACE	rs4459610	SNP_A-2235978	A	T	17	58808166	59052935	58938452
ACE	rs11658531	SNP_A-8646558	A	G	17	58808166	59052935	58941524
ACE	rs8066114	SNP_A-1910897	G	C	17	58808166	59052935	58943572
ACE	rs4611524	SNP_A-2209783	C	T	17	58808166	59052935	58945384
ACE	rs11658641	SNP_A-8609541	T	G	17	58808166	59052935	58964632
ACE	rs7224716	SNP_A-8381931	A	G	17	58808166	59052935	58965125
ACE	rs7225568	SNP_A-8642753	C	T	17	58808166	59052935	58965155
ACE	rs7221979	SNP_A-8435306	G	A	17	58808166	59052935	58968249
ACE	rs12603614	SNP_A-8292250	G	A	17	58808166	59052935	58976435
ACE	rs9896476	SNP_A-8698448	C	G	17	58808166	59052935	58983424
ACE	rs12451850	SNP_A-8512161	C	T	17	58808166	59052935	58986654
ACE	rs12601211	SNP_A-2313311	A	G	17	58808166	59052935	58991153
ACE	rs8069188	SNP_A-1798481	C	T	17	58808166	59052935	58991213
ACE	rs9906747	SNP_A-2309791	A	T	17	58808166	59052935	58993426
ACE	rs17631394	SNP_A-8490639	A	G	17	58808166	59052935	58999732
ACE	rs8077278	SNP_A-8637189	T	G	17	58808166	59052935	59010772
ACE	rs17631783	SNP_A-4243701	T	C	17	58808166	59052935	59041332
ACE	rs9904598	SNP_A-2046136	C	T	17	58808166	59052935	59042560
ACE	rs9911426	SNP_A-2166834	G	T	17	58808166	59052935	59042962
AGT	rs1359865	SNP_A-8666757	C	A	1	228804897	229016564	228811901
AGT	rs11122554	SNP_A-1828392	T	A	1	228804897	229016564	228812038
AGT	rs853463	SNP_A-8666758	T	C	1	228804897	229016564	228812517
AGT	rs12058420	SNP_A-2263791	C	G	1	228804897	229016564	228813609

AGT	rs16852085	SNP_A-2170858	C	T	1	228804897	229016564	228813913
AGT	rs17659489	SNP_A-2238501	C	T	1	228804897	229016564	228815868
AGT	rs11122555	SNP_A-2208047	T	C	1	228804897	229016564	228817306
AGT	rs11122556	SNP_A-2027723	C	G	1	228804897	229016564	228818932
AGT	rs699900	SNP_A-8714970	C	G	1	228804897	229016564	228822031
AGT	rs1410138	SNP_A-8462425	T	C	1	228804897	229016564	228823120
AGT	rs714214	SNP_A-8384171	G	A	1	228804897	229016564	228825228
AGT	rs11122558	SNP_A-2259741	G	C	1	228804897	229016564	228826766
AGT	rs853470	SNP_A-8550025	G	A	1	228804897	229016564	228826936
AGT	rs4846997	SNP_A-8511487	T	C	1	228804897	229016564	228834660
AGT	rs4846998	SNP_A-8377967	T	A	1	228804897	229016564	228834754
AGT	rs11122561	SNP_A-8557427	T	A	1	228804897	229016564	228837142
AGT	rs7529178	SNP_A-1874455	C	T	1	228804897	229016564	228839812
AGT	rs4846863	SNP_A-8597210	G	A	1	228804897	229016564	228839948
AGT	rs12025651	SNP_A-2160160	G	A	1	228804897	229016564	228840585
AGT	rs11122564	SNP_A-8486789	A	C	1	228804897	229016564	228840709
AGT	rs1078198	SNP_A-8529377	T	C	1	228804897	229016564	228841100
AGT	rs1078197	SNP_A-1820781	T	C	1	228804897	229016564	228841474
AGT	rs16852125	SNP_A-2195303	G	A	1	228804897	229016564	228842306
AGT	rs4846999	SNP_A-8525773	A	G	1	228804897	229016564	228845502
AGT	rs11122565	SNP_A-8321179	T	G	1	228804897	229016564	228845889
AGT	rs1887492	SNP_A-1855113	G	A	1	228804897	229016564	228846377
AGT	rs1887491	SNP_A-2292436	G	C	1	228804897	229016564	228846598
AGT	rs1887490	SNP_A-1850365	G	A	1	228804897	229016564	228846617
AGT	rs3827743	SNP_A-8501289	G	A	1	228804897	229016564	228847194
AGT	rs4847000	SNP_A-2314090	G	A	1	228804897	229016564	228848364
AGT	rs3761952	SNP_A-8677210	A	C	1	228804897	229016564	228850898
AGT	rs3789638	SNP_A-8326004	G	C	1	228804897	229016564	228851412
AGT	rs3827744	SNP_A-8566444	C	T	1	228804897	229016564	228853590
AGT	rs9658890	SNP_A-8480066	G	A	1	228804897	229016564	228860849
AGT	rs2225146	SNP_A-1792852	G	A	1	228804897	229016564	228862264
AGT	rs2296802	SNP_A-8561242	G	A	1	228804897	229016564	228865431
AGT	rs16852184	SNP_A-8666767	A	G	1	228804897	229016564	228872137
AGT	rs16852189	SNP_A-8666768	G	A	1	228804897	229016564	228872409
AGT	rs6681346	SNP_A-8444553	A	T	1	228804897	229016564	228877379
AGT	rs1998997	SNP_A-8667023	T	C	1	228804897	229016564	228880706
AGT	rs1467670	SNP_A-2000761	G	T	1	228804897	229016564	228884482
AGT	rs3789651	SNP_A-4276470	A	G	1	228804897	229016564	228884927
AGT	rs2479130	SNP_A-8705721	A	G	1	228804897	229016564	228885034
AGT	rs3789653	SNP_A-2143656	G	A	1	228804897	229016564	228885248
AGT	rs2479131	SNP_A-2053670	T	G	1	228804897	229016564	228885464
AGT	rs3789654	SNP_A-8511748	C	A	1	228804897	229016564	228886915
AGT	rs2479135	SNP_A-8646880	G	A	1	228804897	229016564	228886974
AGT	rs2296798	SNP_A-8580208	G	A	1	228804897	229016564	228887253
AGT	rs2296799	SNP_A-1961060	T	C	1	228804897	229016564	228888107
AGT	---	SNP_A-4218265	T	C	1	228804897	229016564	228888619
AGT	rs2493147	SNP_A-8290630	T	C	1	228804897	229016564	228889526
AGT	rs6656060	SNP_A-8349714	G	A	1	228804897	229016564	228890416
AGT	rs1051038	SNP_A-8593508	A	G	1	228804897	229016564	228895762
AGT	rs12059975	SNP_A-8601007	A	G	1	228804897	229016564	228897681
AGT	rs2493127	SNP_A-2000763	G	A	1	228804897	229016564	228898816
AGT	rs16852352	SNP_A-8385805	T	G	1	228804897	229016564	228903409
AGT	rs943580	SNP_A-8321955	G	A	1	228804897	229016564	228903667
AGT	rs10864770	SNP_A-8494145	A	G	1	228804897	229016564	228904060
AGT	rs11568046	SNP_A-2077287	A	G	1	228804897	229016564	228904738
AGT	rs1926723	SNP_A-1794507	C	T	1	228804897	229016564	228906719

AGT	rs2493129	SNP_A-8301284	A	G	1	228804897	229016564	228907565
AGT	rs11568035	SNP_A-4287554	C	T	1	228804897	229016564	228911313
AGT	rs11122576	SNP_A-4289331	C	T	1	228804897	229016564	228913302
AGT	rs11568030	SNP_A-1938818	T	C	1	228804897	229016564	228913466
AGT	rs11568026	SNP_A-1840348	A	G	1	228804897	229016564	228914146
AGT	rs11122577	SNP_A-1843329	A	C	1	228804897	229016564	228914179
AGT	rs11122578	SNP_A-8412309	A	G	1	228804897	229016564	228914412
AGT	rs11568023	SNP_A-2296476	A	G	1	228804897	229016564	228914802
AGT	rs5046	SNP_A-8411139	A	G	1	228804897	229016564	228917021
AGT	rs2071406	SNP_A-1862100	G	A	1	228804897	229016564	228917264
AGT	rs2071404	SNP_A-4250141	A	C	1	228804897	229016564	228917563
AGT	rs4028824	SNP_A-8355095	G	A	1	228804897	229016564	228920764
AGT	rs7555650	SNP_A-2139551	C	T	1	228804897	229016564	228921622
AGT	rs7549009	SNP_A-4253515	A	G	1	228804897	229016564	228921694
AGT	rs4628514	SNP_A-2160373	C	T	1	228804897	229016564	228922741
AGT	rs1926721	SNP_A-8667039	A	G	1	228804897	229016564	228931453
AGT	rs4847008	SNP_A-4207551	G	T	1	228804897	229016564	228931849
AGT	rs10864775	SNP_A-8693889	T	C	1	228804897	229016564	228932842
AGT	rs2478521	SNP_A-8503049	T	C	1	228804897	229016564	228933737
AGT	rs4846868	SNP_A-2231833	A	G	1	228804897	229016564	228934296
AGT	rs2478518	SNP_A-1987929	G	C	1	228804897	229016564	228934493
AGT	rs2478517	SNP_A-2246438	A	G	1	228804897	229016564	228934510
AGT	rs10864777	SNP_A-1987931	G	T	1	228804897	229016564	228934686
AGT	rs10495299	SNP_A-4212027	G	A	1	228804897	229016564	228936260
AGT	rs6663784	SNP_A-8637895	G	A	1	228804897	229016564	228942330
AGT	rs12409662	SNP_A-8506363	G	C	1	228804897	229016564	228943423
AGT	rs16852515	SNP_A-1987938	C	A	1	228804897	229016564	228943767
AGT	rs2478541	SNP_A-8355356	T	C	1	228804897	229016564	228946575
AGT	rs6662197	SNP_A-8713625	G	A	1	228804897	229016564	228946778
AGT	rs7540252	SNP_A-8390324	C	T	1	228804897	229016564	228949072
AGT	rs2493153	SNP_A-1852552	A	G	1	228804897	229016564	228952814
AGT	rs2478546	SNP_A-8644374	T	C	1	228804897	229016564	228952888
AGT	rs1887208	SNP_A-8317648	G	A	1	228804897	229016564	228952994
AGT	rs16852579	SNP_A-1888430	T	C	1	228804897	229016564	228957896
AGT	rs2493146	SNP_A-2240688	T	C	1	228804897	229016564	228957956
AGT	rs3790958	SNP_A-8610339	C	T	1	228804897	229016564	228964347
AGT	rs2282318	SNP_A-2177257	T	A	1	228804897	229016564	228964854
AGT	rs12121628	SNP_A-8486413	G	A	1	228804897	229016564	228967379
AGT	rs12059555	SNP_A-8484089	A	G	1	228804897	229016564	228967649
AGT	rs12059573	SNP_A-8640604	C	G	1	228804897	229016564	228967721
AGT	rs12027189	SNP_A-8566730	T	C	1	228804897	229016564	228969629
AGT	rs872504	SNP_A-8524038	C	T	1	228804897	229016564	228970882
AGT	rs4847017	SNP_A-1815960	C	T	1	228804897	229016564	228972346
AGT	rs4618981	SNP_A-8652470	T	A	1	228804897	229016564	228972717
AGT	rs16852607	SNP_A-8453303	C	T	1	228804897	229016564	228973197
AGT	rs3790971	SNP_A-8460303	G	A	1	228804897	229016564	228973398
AGT	rs6677009	SNP_A-8709145	C	T	1	228804897	229016564	228973577
AGT	rs12404010	SNP_A-2066575	G	A	1	228804897	229016564	228973854
AGT	rs4520425	SNP_A-1836656	C	T	1	228804897	229016564	228973999
AGT	rs3790972	SNP_A-8667296	A	G	1	228804897	229016564	228975335
AGT	rs16852647	SNP_A-1809278	A	G	1	228804897	229016564	228976658
AGT	rs7517389	SNP_A-1805520	A	G	1	228804897	229016564	228976962
AGT	rs7537410	SNP_A-2304693	A	G	1	228804897	229016564	228979103
AGT	rs12068313	SNP_A-2200739	G	A	1	228804897	229016564	228979241
AGT	rs11122597	SNP_A-2256835	A	G	1	228804897	229016564	228979807
AGT	rs11122598	SNP_A-4274419	G	A	1	228804897	229016564	228979994

AGT	rs12757624	SNP_A-2179822	A	G	1	228804897	229016564	228980744
AGT	rs11804774	SNP_A-4248922	G	C	1	228804897	229016564	228986530
AGT	rs12038826	SNP_A-8610065	C	T	1	228804897	229016564	228989574
AGT	rs12731961	SNP_A-8357795	T	C	1	228804897	229016564	228989936
AGT	rs6661547	SNP_A-2077606	G	A	1	228804897	229016564	228992087
AGT	rs11584791	SNP_A-8301273	A	G	1	228804897	229016564	228992680
AGT	rs2282321	SNP_A-8631891	T	C	1	228804897	229016564	228994974
AGT	rs16852681	SNP_A-1898392	A	G	1	228804897	229016564	228995050
AGT	rs3828128	SNP_A-8585172	A	G	1	228804897	229016564	228995933
AGT	rs11808288	SNP_A-4249842	A	G	1	228804897	229016564	229002027
AGT	rs6667160	SNP_A-8439835	G	A	1	228804897	229016564	229003393
AGT	rs4847020	SNP_A-8513060	G	A	1	228804897	229016564	229008833
AGT	rs11122610	SNP_A-2115458	C	A	1	228804897	229016564	229010982
AGT	rs16852707	SNP_A-2115199	G	A	1	228804897	229016564	229011805
AGT	rs1202519	SNP_A-4226170	T	C	1	228804897	229016564	229013763
AGTR1	rs1522940	SNP_A-2255411	C	A	3	149798355	150043478	149798377
AGTR1	rs1357424	SNP_A-8619402	T	C	3	149798355	150043478	149801516
AGTR1	rs2687661	SNP_A-2278432	T	A	3	149798355	150043478	149801923
AGTR1	rs932226	SNP_A-8500125	A	G	3	149798355	150043478	149817089
AGTR1	rs6803324	SNP_A-2211885	T	C	3	149798355	150043478	149817720
AGTR1	rs188019	SNP_A-8365965	A	G	3	149798355	150043478	149818887
AGTR1	rs1357426	SNP_A-8376958	G	A	3	149798355	150043478	149824969
AGTR1	rs1357427	SNP_A-8488170	T	C	3	149798355	150043478	149825145
AGTR1	rs11718532	SNP_A-8484648	A	C	3	149798355	150043478	149826480
AGTR1	rs1403432	SNP_A-8696218	C	T	3	149798355	150043478	149826710
AGTR1	rs275690	SNP_A-2207374	C	T	3	149798355	150043478	149828216
AGTR1	rs11715970	SNP_A-8427405	A	G	3	149798355	150043478	149829360
AGTR1	rs275696	SNP_A-8375847	A	G	3	149798355	150043478	149835896
AGTR1	rs275697	SNP_A-8530310	T	C	3	149798355	150043478	149836430
AGTR1	rs6440561	SNP_A-4280023	G	A	3	149798355	150043478	149841272
AGTR1	rs6440562	SNP_A-2251525	C	T	3	149798355	150043478	149841395
AGTR1	rs13070340	SNP_A-8442533	T	C	3	149798355	150043478	149841530
AGTR1	rs6440565	SNP_A-8603132	T	C	3	149798355	150043478	149841622
AGTR1	rs9862062	SNP_A-2113145	G	C	3	149798355	150043478	149842414
AGTR1	rs9811115	SNP_A-1841426	T	G	3	149798355	150043478	149842736
AGTR1	rs1492091	SNP_A-2035843	A	C	3	149798355	150043478	149846833
AGTR1	rs2933255	SNP_A-4236305	A	T	3	149798355	150043478	149847437
AGTR1	rs275679	SNP_A-1974767	G	C	3	149798355	150043478	149850993
AGTR1	rs10513336	SNP_A-1974768	A	C	3	149798355	150043478	149851077
AGTR1	rs16860657	SNP_A-8619403	T	C	3	149798355	150043478	149851458
AGTR1	rs275676	SNP_A-8539550	T	C	3	149798355	150043478	149852074
AGTR1	rs275709	SNP_A-8692456	C	T	3	149798355	150043478	149854725
AGTR1	rs275710	SNP_A-8326503	A	T	3	149798355	150043478	149855071
AGTR1	rs275711	SNP_A-2065921	G	A	3	149798355	150043478	149857321
AGTR1	rs1565588	SNP_A-8619404	C	T	3	149798355	150043478	149859899
AGTR1	rs10804731	SNP_A-8514333	C	T	3	149798355	150043478	149861560
AGTR1	rs1492105	SNP_A-8535474	G	A	3	149798355	150043478	149861765
AGTR1	rs9836959	SNP_A-1873869	A	G	3	149798355	150043478	149862397
AGTR1	rs718424	SNP_A-1974770	C	T	3	149798355	150043478	149863233
AGTR1	rs2087737	SNP_A-2147700	C	T	3	149798355	150043478	149864212
AGTR1	rs16860674	SNP_A-1881814	G	A	3	149798355	150043478	149864692
AGTR1	rs903051	SNP_A-8683585	G	A	3	149798355	150043478	149865095
AGTR1	rs17762633	SNP_A-8687368	A	C	3	149798355	150043478	149865762
AGTR1	rs16860677	SNP_A-8440733	G	A	3	149798355	150043478	149866422
AGTR1	rs275702	SNP_A-8357703	C	T	3	149798355	150043478	149866676
AGTR1	rs2934080	SNP_A-8714273	C	T	3	149798355	150043478	149868047

AGTR1	rs2320031	SNP_A-8466927	C	A	3	149798355	150043478	149868971
AGTR1	rs872212	SNP_A-2274493	G	A	3	149798355	150043478	149869437
AGTR1	rs2012052	SNP_A-1832186	A	T	3	149798355	150043478	149869546
AGTR1	rs275674	SNP_A-4238987	G	C	3	149798355	150043478	149879218
AGTR1	rs454530	SNP_A-1974772	G	A	3	149798355	150043478	149883347
AGTR1	rs1492090	SNP_A-8619405	T	A	3	149798355	150043478	149884743
AGTR1	rs2638359	SNP_A-1825911	A	T	3	149798355	150043478	149889073
AGTR1	rs2638358	SNP_A-1836701	T	C	3	149798355	150043478	149889227
AGTR1	rs2638357	SNP_A-4274974	T	C	3	149798355	150043478	149889309
AGTR1	rs2933251	SNP_A-2233133	G	T	3	149798355	150043478	149889489
AGTR1	rs409742	SNP_A-2168102	G	A	3	149798355	150043478	149895055
AGTR1	rs4681157	SNP_A-2026820	C	A	3	149798355	150043478	149895098
AGTR1	rs275651	SNP_A-2079652	A	T	3	149798355	150043478	149897577
AGTR1	rs275652	SNP_A-2035602	T	G	3	149798355	150043478	149897674
AGTR1	rs12721267	SNP_A-1919171	T	G	3	149798355	150043478	149899017
AGTR1	rs2933249	SNP_A-1884603	A	G	3	149798355	150043478	149899210
AGTR1	rs12721286	SNP_A-8538929	G	T	3	149798355	150043478	149901605
AGTR1	rs12721221	SNP_A-8540580	T	C	3	149798355	150043478	149902287
AGTR1	rs2131127	SNP_A-2154498	A	G	3	149798355	150043478	149906833
AGTR1	rs12695877	SNP_A-1885548	C	T	3	149798355	150043478	149909724
AGTR1	rs4681440	SNP_A-2043696	C	T	3	149798355	150043478	149914722
AGTR1	rs12721255	SNP_A-2280404	C	T	3	149798355	150043478	149914903
AGTR1	rs4681443	SNP_A-2087349	A	G	3	149798355	150043478	149915159
AGTR1	rs4681444	SNP_A-2164060	C	A	3	149798355	150043478	149915264
AGTR1	rs1492103	SNP_A-2149351	A	G	3	149798355	150043478	149915654
AGTR1	rs1492100	SNP_A-8418874	T	A	3	149798355	150043478	149920117
AGTR1	rs3772616	SNP_A-8387800	T	C	3	149798355	150043478	149920881
AGTR1	rs4524238	SNP_A-1939005	G	A	3	149798355	150043478	149922478
AGTR1	rs12695891	SNP_A-8524289	A	G	3	149798355	150043478	149923336
AGTR1	rs12695894	SNP_A-4238770	G	A	3	149798355	150043478	149925330
AGTR1	rs12721328	SNP_A-2294086	T	G	3	149798355	150043478	149925644
AGTR1	rs379600	SNP_A-8355836	G	A	3	149798355	150043478	149927175
AGTR1	rs16860760	SNP_A-1974774	G	A	3	149798355	150043478	149927327
AGTR1	rs1826361	SNP_A-8619406	C	A	3	149798355	150043478	149927418
AGTR1	rs12695902	SNP_A-1781484	A	G	3	149798355	150043478	149931108
AGTR1	rs12721234	SNP_A-8410636	T	C	3	149798355	150043478	149938624
AGTR1	rs12695918	SNP_A-2057247	T	C	3	149798355	150043478	149939317
AGTR1	rs1800766	SNP_A-8310675	C	T	3	149798355	150043478	149940332
AGTR1	rs12695923	SNP_A-8692229	T	C	3	149798355	150043478	149940600
AGTR1	rs275646	SNP_A-2192501	C	T	3	149798355	150043478	149946212
AGTR1	rs275644	SNP_A-4199672	T	C	3	149798355	150043478	149948230
AGTR1	rs275643	SNP_A-2104975	A	G	3	149798355	150043478	149948294
AGTR1	rs275642	SNP_A-2259778	A	G	3	149798355	150043478	149948381
AGTR1	rs430349	SNP_A-2230335	C	T	3	149798355	150043478	149948587
AGTR1	rs16860812	SNP_A-2164242	G	C	3	149798355	150043478	149948641
AGTR1	rs172102	SNP_A-8619407	A	G	3	149798355	150043478	149949025
AGTR1	rs13097326	SNP_A-2212642	G	A	3	149798355	150043478	149951436
AGTR1	rs10513338	SNP_A-1974776	T	C	3	149798355	150043478	149952280
AGTR1	rs2675513	SNP_A-1974777	T	G	3	149798355	150043478	149952317
AGTR1	rs16860840	SNP_A-2230834	A	T	3	149798355	150043478	149953118
AGTR1	rs17713681	SNP_A-8529994	C	T	3	149798355	150043478	149960403
AGTR1	rs2934082	SNP_A-8365887	T	G	3	149798355	150043478	149960913
AGTR1	rs16860864	SNP_A-8679262	T	C	3	149798355	150043478	149963196
AGTR1	rs6772419	SNP_A-2238664	G	A	3	149798355	150043478	149963938
AGTR1	rs2934118	SNP_A-2123142	T	C	3	149798355	150043478	149964152
AGTR1	rs1565586	SNP_A-1974778	T	C	3	149798355	150043478	149965021

AGTR1	rs16860871	SNP_A-1974779	C	T	3	149798355	150043478	149965893
AGTR1	rs16860881	SNP_A-8619408	C	G	3	149798355	150043478	149967520
AGTR1	rs7614516	SNP_A-1846268	C	T	3	149798355	150043478	149972797
AGTR1	rs2934114	SNP_A-4216744	C	T	3	149798355	150043478	149972971
AGTR1	rs2934086	SNP_A-8524585	G	A	3	149798355	150043478	149973048
AGTR1	rs2934115	SNP_A-8619409	C	T	3	149798355	150043478	149973786
AGTR1	rs1027311	SNP_A-1916936	G	A	3	149798355	150043478	149978016
AGTR1	rs2934127	SNP_A-8335364	C	T	3	149798355	150043478	149987476
AGTR1	rs9822689	SNP_A-8317223	A	G	3	149798355	150043478	149987561
AGTR1	rs6801709	SNP_A-8555990	T	C	3	149798355	150043478	149988075
AGTR1	rs16860909	SNP_A-2304023	A	G	3	149798355	150043478	149988788
AGTR1	rs11925963	SNP_A-8383567	A	G	3	149798355	150043478	149988801
AGTR1	rs12634627	SNP_A-8543444	C	T	3	149798355	150043478	149989410
AGTR1	rs16860930	SNP_A-8492004	G	A	3	149798355	150043478	149992254
AGTR1	rs13079446	SNP_A-8381225	T	A	3	149798355	150043478	149992304
AGTR1	rs16860946	SNP_A-2134895	C	G	3	149798355	150043478	149997316
AGTR1	rs4681449	SNP_A-8428002	G	A	3	149798355	150043478	150004291
AGTR1	rs2934122	SNP_A-8438135	G	C	3	149798355	150043478	150008061
AGTR1	rs7611062	SNP_A-4272721	A	G	3	149798355	150043478	150008340
AGTR1	rs7644988	SNP_A-4275506	G	T	3	149798355	150043478	150008355
AGTR1	rs6762761	SNP_A-2242654	A	C	3	149798355	150043478	150013536
AGTR1	rs16860970	SNP_A-1897663	G	A	3	149798355	150043478	150015874
AGTR1	rs17714660	SNP_A-4224372	T	C	3	149798355	150043478	150016087
AGTR1	rs6798306	SNP_A-8656182	G	T	3	149798355	150043478	150017044
AGTR1	rs16860973	SNP_A-8349533	A	C	3	149798355	150043478	150017560
AGTR1	rs4681158	SNP_A-8647207	T	C	3	149798355	150043478	150021974
AGTR1	rs10935727	SNP_A-2052259	A	G	3	149798355	150043478	150028390
AGTR1	rs9821558	SNP_A-8445120	A	G	3	149798355	150043478	150030470
AGTR1	rs7642396	SNP_A-2170939	C	T	3	149798355	150043478	150032179
AGTR1	rs4144706	SNP_A-1819672	C	T	3	149798355	150043478	150032525
AGTR1	rs16861027	SNP_A-4245331	T	G	3	149798355	150043478	150032814
AGTR1	rs7617340	SNP_A-8535705	C	T	3	149798355	150043478	150033212
AGTR1	rs11711637	SNP_A-8686519	G	A	3	149798355	150043478	150033517
AGTR1	rs16861036	SNP_A-8518592	T	A	3	149798355	150043478	150033537
AGTR1	rs9830213	SNP_A-2209939	C	T	3	149798355	150043478	150035229
AGTR1	rs1318579	SNP_A-8619410	G	T	3	149798355	150043478	150036250
AGTR1	rs3772603	SNP_A-4263823	G	A	3	149798355	150043478	150037079
AGTR1	rs3772602	SNP_A-8440219	G	A	3	149798355	150043478	150037358
AGTR1	rs3772601	SNP_A-1974782	T	C	3	149798355	150043478	150037449
AGTR1	rs11919702	SNP_A-1787866	G	A	3	149798355	150043478	150037915
ANG	rs6576288	SNP_A-2028393	T	C	14	20122609	20332180	20123097
ANG	rs1756542	SNP_A-2204728	G	C	14	20122609	20332180	20123831
ANG	rs4981259	SNP_A-1904568	T	C	14	20122609	20332180	20128177
ANG	rs3827902	SNP_A-8701289	G	T	14	20122609	20332180	20128265
ANG	rs10151120	SNP_A-1865769	C	A	14	20122609	20332180	20130127
ANG	rs4981260	SNP_A-2228865	G	C	14	20122609	20332180	20130257
ANG	rs4981261	SNP_A-1953477	G	C	14	20122609	20332180	20130287
ANG	rs4981262	SNP_A-8397424	T	G	14	20122609	20332180	20130342
ANG	rs4981265	SNP_A-1898230	C	T	14	20122609	20332180	20130488
ANG	rs10131138	SNP_A-8562911	G	A	14	20122609	20332180	20131007
ANG	rs7159639	SNP_A-8410750	G	C	14	20122609	20332180	20134857
ANG	rs17242727	SNP_A-8573201	A	G	14	20122609	20332180	20135236
ANG	rs1272513	SNP_A-8359898	A	G	14	20122609	20332180	20139161
ANG	rs1243597	SNP_A-8295677	A	C	14	20122609	20332180	20140350
ANG	rs12323741	SNP_A-8558159	A	G	14	20122609	20332180	20140666
ANG	rs8018826	SNP_A-4258341	T	C	14	20122609	20332180	20141227

ANG	rs8016310	SNP_A-8313401	A	G	14	20122609	20332180	20142544
ANG	rs17242741	SNP_A-8331188	G	A	14	20122609	20332180	20147186
ANG	rs1243655	SNP_A-8529633	C	G	14	20122609	20332180	20147675
ANG	rs1243657	SNP_A-8512242	C	T	14	20122609	20332180	20150888
ANG	rs1243658	SNP_A-4206211	A	G	14	20122609	20332180	20151250
ANG	rs1243659	SNP_A-2023699	T	C	14	20122609	20332180	20151347
ANG	rs1756548	SNP_A-8635380	A	C	14	20122609	20332180	20151498
ANG	---	SNP_A-2061432	A	C	14	20122609	20332180	20151584
ANG	rs1243661	SNP_A-8455587	G	T	14	20122609	20332180	20153273
ANG	rs1243675	SNP_A-8533789	A	G	14	20122609	20332180	20162443
ANG	rs12432620	SNP_A-8543581	A	G	14	20122609	20332180	20175089
ANG	rs10498270	SNP_A-8603383	A	G	14	20122609	20332180	20176367
ANG	rs8012233	SNP_A-2302136	T	C	14	20122609	20332180	20177904
ANG	rs8007341	SNP_A-8521415	C	T	14	20122609	20332180	20177937
ANG	rs11844727	SNP_A-1879759	G	A	14	20122609	20332180	20178271
ANG	rs17277515	SNP_A-2030058	A	G	14	20122609	20332180	20178749
ANG	rs17114309	SNP_A-4231085	G	A	14	20122609	20332180	20178805
ANG	rs11622794	SNP_A-2191865	G	A	14	20122609	20332180	20179566
ANG	rs11156622	SNP_A-4256647	T	C	14	20122609	20332180	20179699
ANG	rs2115656	SNP_A-1934081	C	G	14	20122609	20332180	20181723
ANG	rs2319378	SNP_A-4293759	G	A	14	20122609	20332180	20182100
ANG	rs7158129	SNP_A-8631247	G	A	14	20122609	20332180	20182515
ANG	rs12147472	SNP_A-8566770	G	A	14	20122609	20332180	20186124
ANG	rs10135437	SNP_A-8671961	A	G	14	20122609	20332180	20193663
ANG	rs12893510	SNP_A-1915516	C	T	14	20122609	20332180	20194287
ANG	rs8003288	SNP_A-2191884	C	T	14	20122609	20332180	20194874
ANG	rs11620733	SNP_A-8604126	A	G	14	20122609	20332180	20195678
ANG	rs4981300	SNP_A-8690702	A	G	14	20122609	20332180	20195878
ANG	rs12431961	SNP_A-4231087	A	G	14	20122609	20332180	20201291
ANG	rs7158152	SNP_A-4256132	A	G	14	20122609	20332180	20203164
ANG	rs10145023	SNP_A-8312043	T	C	14	20122609	20332180	20205289
ANG	rs7143439	SNP_A-8516373	T	C	14	20122609	20332180	20206831
ANG	rs10145502	SNP_A-8467851	T	A	14	20122609	20332180	20213882
ANG	rs10498273	SNP_A-8604131	C	G	14	20122609	20332180	20214639
ANG	rs11629118	SNP_A-8649405	T	C	14	20122609	20332180	20214895
ANG	rs12588573	SNP_A-8581361	T	C	14	20122609	20332180	20216424
ANG	rs12587456	SNP_A-8715882	A	G	14	20122609	20332180	20219948
ANG	rs9322855	SNP_A-2191902	A	C	14	20122609	20332180	20223139
ANG	rs1010461	SNP_A-4290447	A	C	14	20122609	20332180	20223628
ANG	rs5019558	SNP_A-2309708	C	G	14	20122609	20332180	20224089
ANG	rs17114671	SNP_A-1955851	C	T	14	20122609	20332180	20225110
ANG	rs4470055	SNP_A-8407543	A	G	14	20122609	20332180	20226031
ANG	rs8008440	SNP_A-2245322	A	G	14	20122609	20332180	20226288
ANG	rs4982325	SNP_A-8656787	T	C	14	20122609	20332180	20226676
ANG	rs11156631	SNP_A-1936187	G	A	14	20122609	20332180	20229446
ANG	rs8010599	SNP_A-8431147	C	T	14	20122609	20332180	20234674
ANG	rs1999343	SNP_A-2250534	G	A	14	20122609	20332180	20235319
ANG	rs17242790	SNP_A-2279696	A	G	14	20122609	20332180	20236067
ANG	rs17211649	SNP_A-1953126	A	G	14	20122609	20332180	20236123
ANG	rs1320480	SNP_A-2033470	T	C	14	20122609	20332180	20245929
ANG	rs4982343	SNP_A-1794428	C	G	14	20122609	20332180	20248238
ANG	rs8004317	SNP_A-2249696	T	C	14	20122609	20332180	20249121
ANG	rs11628398	SNP_A-2298521	A	G	14	20122609	20332180	20252640
ANG	rs11156638	SNP_A-2260592	G	C	14	20122609	20332180	20258525
ANG	rs4982347	SNP_A-1871149	C	T	14	20122609	20332180	20259512
ANG	rs11156644	SNP_A-2191935	A	G	14	20122609	20332180	20271429

ANG	rs8012716	SNP_A-1950950	A	G	14	20122609	20332180	20272176
ANG	rs17277606	SNP_A-8345195	T	G	14	20122609	20332180	20272473
ANG	rs9672003	SNP_A-8621593	G	A	14	20122609	20332180	20272845
ANG	rs9671547	SNP_A-2274631	T	C	14	20122609	20332180	20273172
ANG	rs17308520	SNP_A-8402570	A	C	14	20122609	20332180	20273244
ANG	rs2319444	SNP_A-2052905	G	C	14	20122609	20332180	20273790
ANG	rs8019026	SNP_A-2092744	C	T	14	20122609	20332180	20275863
ANG	rs8013177	SNP_A-2154766	G	C	14	20122609	20332180	20275932
ANG	rs7153831	SNP_A-2084424	G	A	14	20122609	20332180	20276579
ANG	rs944440	SNP_A-1853096	C	T	14	20122609	20332180	20277396
ANG	rs8019599	SNP_A-8590612	G	A	14	20122609	20332180	20277477
ANG	rs17308534	SNP_A-8676568	A	C	14	20122609	20332180	20277488
ANG	rs8006368	SNP_A-1809101	T	C	14	20122609	20332180	20278496
ANG	rs17308541	SNP_A-2051706	G	A	14	20122609	20332180	20280006
ANG	rs1888567	SNP_A-2050991	G	A	14	20122609	20332180	20283701
ANG	rs11621687	SNP_A-8482975	T	G	14	20122609	20332180	20283810
ANG	rs2873979	SNP_A-1812098	G	A	14	20122609	20332180	20283863
ANG	rs1815437	SNP_A-2217327	A	G	14	20122609	20332180	20283985
ANG	rs1815438	SNP_A-8600336	T	C	14	20122609	20332180	20284120
ANG	rs3790066	SNP_A-2100458	C	T	14	20122609	20332180	20284485
ANG	rs4981328	SNP_A-8309032	C	G	14	20122609	20332180	20284645
ANG	rs8181973	SNP_A-2276928	G	A	14	20122609	20332180	20284910
ANG	rs10136680	SNP_A-8306260	T	C	14	20122609	20332180	20285532
ANG	rs4982352	SNP_A-1892960	C	T	14	20122609	20332180	20288180
ANG	rs2145870	SNP_A-2098798	G	A	14	20122609	20332180	20288504
ANG	rs10141260	SNP_A-8492761	G	A	14	20122609	20332180	20292291
ANG	rs970014	SNP_A-4204202	C	T	14	20122609	20332180	20292766
ANG	rs970015	SNP_A-2088188	G	A	14	20122609	20332180	20292831
ANG	rs1022739	SNP_A-8482246	C	A	14	20122609	20332180	20297013
ANG	rs1022740	SNP_A-4198081	A	G	14	20122609	20332180	20297342
ANG	rs1957333	SNP_A-1948101	T	G	14	20122609	20332180	20297432
ANG	rs8004417	SNP_A-4253819	G	C	14	20122609	20332180	20299040
ANG	rs11848221	SNP_A-2217146	A	G	14	20122609	20332180	20299145
ANG	rs11624594	SNP_A-2183869	A	G	14	20122609	20332180	20300012
ANG	rs12895364	SNP_A-4259413	C	T	14	20122609	20332180	20300040
ANG	rs10139490	SNP_A-8395852	T	A	14	20122609	20332180	20300174
ANG	rs10150727	SNP_A-2202538	T	C	14	20122609	20332180	20300264
ANG	rs12895983	SNP_A-8310849	C	T	14	20122609	20332180	20300416
ANG	rs10131121	SNP_A-8392460	G	C	14	20122609	20332180	20301415
ANG	rs10131142	SNP_A-8531122	A	C	14	20122609	20332180	20301501
ANG	rs762038	SNP_A-2075234	G	C	14	20122609	20332180	20304608
ANG	rs2009021	SNP_A-4257242	C	G	14	20122609	20332180	20304838
ANG	rs2093359	SNP_A-2101963	G	A	14	20122609	20332180	20305259
ANG	rs3748339	SNP_A-1834589	G	A	14	20122609	20332180	20308607
ANG	rs3827907	SNP_A-1790660	T	C	14	20122609	20332180	20308638
ANG	rs17096253	SNP_A-8531718	G	C	14	20122609	20332180	20308944
ANG	rs1951419	SNP_A-8558469	T	G	14	20122609	20332180	20310513
ANG	rs7152592	SNP_A-8660249	C	T	14	20122609	20332180	20314223
ANG	rs12586813	SNP_A-8602141	G	A	14	20122609	20332180	20314319
ANG	rs4312222	SNP_A-8634322	G	T	14	20122609	20332180	20315379
ANG	rs986192	SNP_A-1848333	A	G	14	20122609	20332180	20317060
ANG	rs1045922	SNP_A-4290459	G	A	14	20122609	20332180	20319964
ANG	rs5024915	SNP_A-2223266	T	C	14	20122609	20332180	20325564
ANG	rs4982360	SNP_A-8717497	G	A	14	20122609	20332180	20325966
ANG	rs12881063	SNP_A-8543611	C	G	14	20122609	20332180	20326016
ANG	rs8013362	SNP_A-2085268	T	A	14	20122609	20332180	20326763

ANG	rs8009569	SNP_A-2128484	T	G	14	20122609	20332180	20329136
ANG	rs11847602	SNP_A-8709330	G	A	14	20122609	20332180	20329875
EDN1	rs10484256	SNP_A-4211524	A	G	6	12298599	12505399	12303136
EDN1	rs16872422	SNP_A-2139920	T	A	6	12298599	12505399	12303625
EDN1	rs9471162	SNP_A-2049652	T	C	6	12298599	12505399	12304476
EDN1	rs9380894	SNP_A-2280276	T	C	6	12298599	12505399	12304590
EDN1	rs1040992	SNP_A-8406211	T	G	6	12298599	12505399	12304896
EDN1	rs1040993	SNP_A-8495124	C	T	6	12298599	12505399	12304924
EDN1	rs1040994	SNP_A-2070218	A	C	6	12298599	12505399	12305199
EDN1	rs11969649	SNP_A-4192805	A	C	6	12298599	12505399	12306289
EDN1	rs219974	SNP_A-1935288	T	C	6	12298599	12505399	12306661
EDN1	rs2012560	SNP_A-4265508	C	G	6	12298599	12505399	12308713
EDN1	rs1022848	SNP_A-4265509	A	G	6	12298599	12505399	12308758
EDN1	rs1023483	SNP_A-2175953	A	G	6	12298599	12505399	12308932
EDN1	rs2187912	SNP_A-8388494	C	T	6	12298599	12505399	12309022
EDN1	rs9380903	SNP_A-1903933	G	A	6	12298599	12505399	12309766
EDN1	rs219985	SNP_A-8667491	A	T	6	12298599	12505399	12316651
EDN1	rs219986	SNP_A-8667492	G	T	6	12298599	12505399	12316805
EDN1	rs11966018	SNP_A-8462538	G	C	6	12298599	12505399	12317214
EDN1	rs219987	SNP_A-8667493	A	C	6	12298599	12505399	12317346
EDN1	rs220012	SNP_A-8667494	C	T	6	12298599	12505399	12322593
EDN1	rs3021344	SNP_A-2253196	A	G	6	12298599	12505399	12324274
EDN1	rs12662776	SNP_A-8445176	T	G	6	12298599	12505399	12324462
EDN1	rs1794854	SNP_A-8375070	C	T	6	12298599	12505399	12324489
EDN1	rs1794856	SNP_A-1888919	G	A	6	12298599	12505399	12324632
EDN1	rs10755709	SNP_A-8417358	A	G	6	12298599	12505399	12324952
EDN1	rs12208557	SNP_A-8425467	T	G	6	12298599	12505399	12325106
EDN1	rs9380917	SNP_A-4219730	G	A	6	12298599	12505399	12325249
EDN1	rs7356945	SNP_A-8630333	C	T	6	12298599	12505399	12325408
EDN1	rs4711607	SNP_A-2229953	A	G	6	12298599	12505399	12325772
EDN1	rs6908010	SNP_A-2226979	T	G	6	12298599	12505399	12325985
EDN1	rs10807220	SNP_A-8544098	C	A	6	12298599	12505399	12326358
EDN1	rs12209755	SNP_A-8487008	G	A	6	12298599	12505399	12330912
EDN1	rs9367042	SNP_A-8332294	A	G	6	12298599	12505399	12331099
EDN1	rs2144221	SNP_A-8424324	C	T	6	12298599	12505399	12331506
EDN1	rs2180030	SNP_A-2230864	A	G	6	12298599	12505399	12331615
EDN1	rs6922296	SNP_A-2242578	A	G	6	12298599	12505399	12331675
EDN1	rs17592421	SNP_A-4276815	G	A	6	12298599	12505399	12331908
EDN1	rs9471238	SNP_A-8594326	T	C	6	12298599	12505399	12332314
EDN1	rs10947850	SNP_A-2113655	C	T	6	12298599	12505399	12332702
EDN1	rs1321167	SNP_A-2299906	A	G	6	12298599	12505399	12333013
EDN1	rs9296319	SNP_A-1937613	A	G	6	12298599	12505399	12334486
EDN1	rs6458133	SNP_A-2231973	C	T	6	12298599	12505399	12334672
EDN1	rs9380934	SNP_A-8402809	T	C	6	12298599	12505399	12337924
EDN1	rs10947858	SNP_A-8491240	C	T	6	12298599	12505399	12341874
EDN1	rs6919706	SNP_A-2097862	C	A	6	12298599	12505399	12342796
EDN1	rs9357318	SNP_A-8673004	A	G	6	12298599	12505399	12345980
EDN1	rs10947862	SNP_A-1886142	A	T	6	12298599	12505399	12350383
EDN1	rs10456479	SNP_A-4249686	T	G	6	12298599	12505399	12352833
EDN1	rs10456105	SNP_A-2039673	A	G	6	12298599	12505399	12355596
EDN1	rs500843	SNP_A-8498840	C	T	6	12298599	12505399	12358908
EDN1	rs511319	SNP_A-1830473	C	G	6	12298599	12505399	12359174
EDN1	rs4714340	SNP_A-8302281	C	T	6	12298599	12505399	12363276
EDN1	rs12201479	SNP_A-2059623	C	T	6	12298599	12505399	12364120
EDN1	rs9369198	SNP_A-1818592	A	C	6	12298599	12505399	12365414
EDN1	rs7744944	SNP_A-8375417	C	T	6	12298599	12505399	12366280

EDN1	rs6458155	SNP_A-8429014	C	T	6	12298599	12505399	12369907
EDN1	rs13192093	SNP_A-8509204	A	G	6	12298599	12505399	12371679
EDN1	rs4145451	SNP_A-2182445	A	C	6	12298599	12505399	12372611
EDN1	rs1321055	SNP_A-4265510	T	A	6	12298599	12505399	12372985
EDN1	rs9349158	SNP_A-8297722	C	A	6	12298599	12505399	12382974
EDN1	rs10807242	SNP_A-2221298	G	C	6	12298599	12505399	12387672
EDN1	rs9369217	SNP_A-1906655	T	C	6	12298599	12505399	12391748
EDN1	rs13198076	SNP_A-8392420	G	A	6	12298599	12505399	12394608
EDN1	rs9380978	SNP_A-1940317	G	A	6	12298599	12505399	12394824
EDN1	rs1800542	SNP_A-8704804	G	A	6	12298599	12505399	12400514
EDN1	rs2070699	SNP_A-2184379	T	G	6	12298599	12505399	12400758
EDN1	rs1476046	SNP_A-8692445	A	G	6	12298599	12505399	12401207
EDN1	rs9296343	SNP_A-8406891	G	C	6	12298599	12505399	12401519
EDN1	rs10478723	SNP_A-1902497	G	A	6	12298599	12505399	12403447
EDN1	rs1626492	SNP_A-2067429	G	A	6	12298599	12505399	12403489
EDN1	rs6912834	SNP_A-1796216	G	A	6	12298599	12505399	12403521
EDN1	rs1629862	SNP_A-8395565	A	G	6	12298599	12505399	12403862
EDN1	rs9296344	SNP_A-4194062	T	C	6	12298599	12505399	12405014
EDN1	rs4714383	SNP_A-2200484	C	T	6	12298599	12505399	12405468
EDN1	rs16872592	SNP_A-1985117	A	G	6	12298599	12505399	12409307
EDN1	rs7356986	SNP_A-4260964	G	A	6	12298599	12505399	12409448
EDN1	rs11755836	SNP_A-8312032	A	C	6	12298599	12505399	12410555
EDN1	rs9462653	SNP_A-8370196	C	T	6	12298599	12505399	12412393
EDN1	rs16872602	SNP_A-8717236	A	C	6	12298599	12505399	12412815
EDN1	rs6458188	SNP_A-8597386	T	A	6	12298599	12505399	12414123
EDN1	rs17594577	SNP_A-8667495	A	G	6	12298599	12505399	12414218
EDN1	rs9471439	SNP_A-8703080	A	C	6	12298599	12505399	12415840
EDN1	rs16872612	SNP_A-8295447	C	G	6	12298599	12505399	12416068
EDN1	rs16872628	SNP_A-2092114	T	C	6	12298599	12505399	12421381
EDN1	rs6458193	SNP_A-2094708	A	G	6	12298599	12505399	12425716
EDN1	rs17765653	SNP_A-8616762	T	C	6	12298599	12505399	12427513
EDN1	rs16872657	SNP_A-2263709	G	A	6	12298599	12505399	12433747
EDN1	rs9381033	SNP_A-2083042	A	G	6	12298599	12505399	12434176
EDN1	rs9381035	SNP_A-1892498	A	G	6	12298599	12505399	12434705
EDN1	rs7750360	SNP_A-8659163	A	G	6	12298599	12505399	12437181
EDN1	rs7760229	SNP_A-8594546	G	A	6	12298599	12505399	12444602
EDN1	rs12530046	SNP_A-8636175	C	T	6	12298599	12505399	12446145
EDN1	rs7758251	SNP_A-8315345	A	G	6	12298599	12505399	12447036
EDN1	rs17765682	SNP_A-2161958	C	T	6	12298599	12505399	12447226
EDN1	rs9471507	SNP_A-8648831	T	C	6	12298599	12505399	12447766
EDN1	rs1317584	SNP_A-2087026	G	A	6	12298599	12505399	12450775
EDN1	rs10947954	SNP_A-1816008	T	G	6	12298599	12505399	12451947
EDN1	rs10807258	SNP_A-8484350	C	T	6	12298599	12505399	12452248
EDN1	rs16872704	SNP_A-8706577	A	G	6	12298599	12505399	12453679
EDN1	rs2327544	SNP_A-4265511	A	G	6	12298599	12505399	12453955
EDN1	rs2095345	SNP_A-8626008	A	G	6	12298599	12505399	12454856
EDN1	rs6899475	SNP_A-8337164	C	T	6	12298599	12505399	12457723
EDN1	rs6903876	SNP_A-8651986	G	C	6	12298599	12505399	12457825
EDN1	rs2095342	SNP_A-8427740	T	C	6	12298599	12505399	12459987
EDN1	rs2050288	SNP_A-2172631	T	C	6	12298599	12505399	12462373
EDN1	rs12332935	SNP_A-8524768	T	C	6	12298599	12505399	12462667
EDN1	rs17615558	SNP_A-8631122	T	G	6	12298599	12505399	12463160
EDN1	rs971054	SNP_A-2311624	A	G	6	12298599	12505399	12464453
EDN1	rs16872713	SNP_A-8658622	C	T	6	12298599	12505399	12466835
EDN1	rs6925906	SNP_A-2056912	A	G	6	12298599	12505399	12467288
EDN1	rs6926651	SNP_A-4304087	G	A	6	12298599	12505399	12467703

EDN1	rs6458226	SNP_A-8321996	C	T	6	12298599	12505399	12479841
EDN1	rs16872733	SNP_A-1794432	G	A	6	12298599	12505399	12480275
EDN1	rs6458227	SNP_A-8610750	C	A	6	12298599	12505399	12481942
EDN1	rs9784878	SNP_A-1924679	C	G	6	12298599	12505399	12490217
EDN1	rs9462731	SNP_A-1985119	G	A	6	12298599	12505399	12491721
EDN1	rs9471621	SNP_A-8547831	T	G	6	12298599	12505399	12492550
EDN1	rs10947982	SNP_A-1840161	G	A	6	12298599	12505399	12492958
EDN1	rs9394825	SNP_A-8652479	C	T	6	12298599	12505399	12497308
EDN1	rs6922952	SNP_A-1840912	T	C	6	12298599	12505399	12499327
EDN1	rs994632	SNP_A-2268980	A	G	6	12298599	12505399	12499959
EDN1	rs7774034	SNP_A-1877632	T	G	6	12298599	12505399	12500116
EDN1	rs9394827	SNP_A-2264266	A	G	6	12298599	12505399	12500746
EDN1	rs7771680	SNP_A-8436000	C	T	6	12298599	12505399	12504008
EDN1	rs16872754	SNP_A-8421411	G	A	6	12298599	12505399	12504124
EDNRA	rs12508797	SNP_A-8441046	T	C	4	148521575	148785555	148522645
EDNRA	rs11939043	SNP_A-8627493	G	T	4	148521575	148785555	148530131
EDNRA	rs1864246	SNP_A-8476235	A	C	4	148521575	148785555	148536952
EDNRA	rs2042998	SNP_A-2010236	C	T	4	148521575	148785555	148540675
EDNRA	rs12647961	SNP_A-8402875	T	A	4	148521575	148785555	148543613
EDNRA	rs2195469	SNP_A-2217795	C	T	4	148521575	148785555	148548006
EDNRA	rs17023196	SNP_A-8398389	C	T	4	148521575	148785555	148548318
EDNRA	rs6537474	SNP_A-1847909	T	C	4	148521575	148785555	148548398
EDNRA	rs17023204	SNP_A-8645766	T	C	4	148521575	148785555	148549096
EDNRA	rs9995888	SNP_A-8696799	G	A	4	148521575	148785555	148552286
EDNRA	rs12511134	SNP_A-8560625	T	C	4	148521575	148785555	148555322
EDNRA	rs1429116	SNP_A-8385494	A	G	4	148521575	148785555	148558612
EDNRA	rs1429117	SNP_A-4280461	A	G	4	148521575	148785555	148560383
EDNRA	rs11947440	SNP_A-8426113	C	T	4	148521575	148785555	148562367
EDNRA	rs1896819	SNP_A-8663062	A	C	4	148521575	148785555	148564195
EDNRA	rs17612591	SNP_A-8663063	T	C	4	148521575	148785555	148565803
EDNRA	rs11722693	SNP_A-8697365	G	A	4	148521575	148785555	148570809
EDNRA	rs10004926	SNP_A-8418133	C	T	4	148521575	148785555	148572732
EDNRA	rs4835398	SNP_A-8335651	G	T	4	148521575	148785555	148572873
EDNRA	rs17023259	SNP_A-2230081	T	A	4	148521575	148785555	148581870
EDNRA	rs3943850	SNP_A-1824608	A	G	4	148521575	148785555	148582094
EDNRA	rs1560231	SNP_A-8404439	G	A	4	148521575	148785555	148582403
EDNRA	rs11944922	SNP_A-8382280	A	G	4	148521575	148785555	148582932
EDNRA	rs4631019	SNP_A-2191923	A	G	4	148521575	148785555	148586417
EDNRA	rs11731134	SNP_A-1873837	C	G	4	148521575	148785555	148599064
EDNRA	rs1400560	SNP_A-2030339	G	T	4	148521575	148785555	148599582
EDNRA	rs6537478	SNP_A-8335652	C	T	4	148521575	148785555	148604544
EDNRA	rs6537479	SNP_A-4214975	A	G	4	148521575	148785555	148604558
EDNRA	rs4469055	SNP_A-2185372	G	C	4	148521575	148785555	148605171
EDNRA	rs12642328	SNP_A-4287253	T	C	4	148521575	148785555	148605757
EDNRA	rs4333164	SNP_A-8494646	C	T	4	148521575	148785555	148616377
EDNRA	rs10305840	SNP_A-4224482	C	T	4	148521575	148785555	148619950
EDNRA	rs6841581	SNP_A-1914964	G	A	4	148521575	148785555	148620640
EDNRA	rs10305845	SNP_A-4202906	T	G	4	148521575	148785555	148620848
EDNRA	rs984457	SNP_A-2110850	T	C	4	148521575	148785555	148622935
EDNRA	rs984458	SNP_A-2086040	C	T	4	148521575	148785555	148622999
EDNRA	rs10305851	SNP_A-1811849	G	C	4	148521575	148785555	148623079
EDNRA	rs11942855	SNP_A-8413530	G	A	4	148521575	148785555	148630628
EDNRA	rs17023363	SNP_A-8650069	A	G	4	148521575	148785555	148631862
EDNRA	rs17023371	SNP_A-2253276	T	A	4	148521575	148785555	148633916
EDNRA	rs17023373	SNP_A-2058276	G	T	4	148521575	148785555	148634068
EDNRA	rs17612742	SNP_A-2273801	C	T	4	148521575	148785555	148634101

EDNRA	rs10305875	SNP_A-1789664	G	A	4	148521575	148785555	148636339
EDNRA	rs10024834	SNP_A-2082107	G	A	4	148521575	148785555	148640086
EDNRA	rs6840756	SNP_A-8690512	A	C	4	148521575	148785555	148640804
EDNRA	rs17474816	SNP_A-2300368	G	A	4	148521575	148785555	148641545
EDNRA	rs16998721	SNP_A-1781665	A	C	4	148521575	148785555	148641586
EDNRA	rs6848108	SNP_A-8641590	G	A	4	148521575	148785555	148642075
EDNRA	rs6537485	SNP_A-2182814	T	A	4	148521575	148785555	148647977
EDNRA	rs4591581	SNP_A-8496053	G	A	4	148521575	148785555	148648552
EDNRA	rs9997823	SNP_A-8593396	A	G	4	148521575	148785555	148653052
EDNRA	rs10305885	SNP_A-8555219	A	T	4	148521575	148785555	148653114
EDNRA	rs6537489	SNP_A-8442619	T	C	4	148521575	148785555	148653596
EDNRA	rs9308221	SNP_A-8690364	T	C	4	148521575	148785555	148654292
EDNRA	rs6827096	SNP_A-1934322	T	C	4	148521575	148785555	148654896
EDNRA	rs9999853	SNP_A-8663064	C	T	4	148521575	148785555	148656321
EDNRA	rs6812093	SNP_A-1979341	T	A	4	148521575	148785555	148656605
EDNRA	rs6822565	SNP_A-4264548	C	T	4	148521575	148785555	148656962
EDNRA	rs1517135	SNP_A-1979343	C	T	4	148521575	148785555	148657004
EDNRA	rs11939191	SNP_A-4264549	G	A	4	148521575	148785555	148658165
EDNRA	rs7674137	SNP_A-8663065	A	G	4	148521575	148785555	148659051
EDNRA	rs4835412	SNP_A-8695335	G	A	4	148521575	148785555	148660589
EDNRA	rs10003447	SNP_A-4248639	T	C	4	148521575	148785555	148666829
EDNRA	rs4639051	SNP_A-1979345	A	G	4	148521575	148785555	148668320
EDNRA	rs2048894	SNP_A-1935557	A	G	4	148521575	148785555	148671284
EDNRA	rs10305908	SNP_A-8660319	T	C	4	148521575	148785555	148674569
EDNRA	rs9307838	SNP_A-8613874	T	C	4	148521575	148785555	148674782
EDNRA	rs3756022	SNP_A-1802358	C	T	4	148521575	148785555	148676240
EDNRA	rs10305920	SNP_A-1894172	G	A	4	148521575	148785555	148676955
EDNRA	rs10305921	SNP_A-1943735	C	T	4	148521575	148785555	148677002
EDNRA	rs6841799	SNP_A-4224362	G	C	4	148521575	148785555	148680240
EDNRA	rs5333	SNP_A-1855680	T	C	4	148521575	148785555	148680487
EDNRA	rs5334	SNP_A-2028694	A	G	4	148521575	148785555	148680523
EDNRA	rs10305927	SNP_A-2060782	A	C	4	148521575	148785555	148681141
EDNRA	rs10305928	SNP_A-2181176	A	G	4	148521575	148785555	148682292
EDNRA	rs5341	SNP_A-1883747	T	C	4	148521575	148785555	148684049
EDNRA	rs10028838	SNP_A-2279445	T	G	4	148521575	148785555	148690782
EDNRA	rs11734418	SNP_A-2231622	A	T	4	148521575	148785555	148691903
EDNRA	rs12647366	SNP_A-2058266	C	T	4	148521575	148785555	148696673
EDNRA	rs1517137	SNP_A-2168154	T	C	4	148521575	148785555	148697327
EDNRA	rs1517131	SNP_A-1979346	A	G	4	148521575	148785555	148702718
EDNRA	rs17613180	SNP_A-8696257	A	G	4	148521575	148785555	148705749
EDNRA	rs2090422	SNP_A-8553057	C	T	4	148521575	148785555	148707995
EDNRA	rs1914331	SNP_A-1832620	C	A	4	148521575	148785555	148719626
EDNRA	rs1914329	SNP_A-8524515	G	A	4	148521575	148785555	148719987
EDNRA	rs7675774	SNP_A-4196399	A	G	4	148521575	148785555	148722748
EDNRA	rs11099658	SNP_A-1906157	T	G	4	148521575	148785555	148723823
EDNRA	rs6535545	SNP_A-8346604	A	G	4	148521575	148785555	148733120
EDNRA	rs9998608	SNP_A-8708197	C	T	4	148521575	148785555	148736327
EDNRA	rs11943395	SNP_A-2143898	A	G	4	148521575	148785555	148740688
EDNRA	rs4835438	SNP_A-2071702	A	G	4	148521575	148785555	148741744
EDNRA	rs11933787	SNP_A-8463483	T	G	4	148521575	148785555	148741810
EDNRA	rs17613313	SNP_A-1961522	T	C	4	148521575	148785555	148742883
EDNRA	rs2090424	SNP_A-2191593	A	G	4	148521575	148785555	148744302
EDNRA	rs6824884	SNP_A-8368525	A	G	4	148521575	148785555	148750995
EDNRA	rs10776520	SNP_A-8640805	G	T	4	148521575	148785555	148753332
EDNRA	rs6846220	SNP_A-8521713	A	C	4	148521575	148785555	148754567
EDNRA	rs931779	SNP_A-2085313	T	C	4	148521575	148785555	148755086

EDNRA	rs10519911	SNP_A-4154924	C	T	4	148521575	148785555	148770040
EDNRA	rs17023581	SNP_A-8604211	G	A	4	148521575	148785555	148775310
EDNRA	rs2718447	SNP_A-1979347	C	T	4	148521575	148785555	148776142
EDNRB	rs1668633	SNP_A-2222835	C	T	13	77267625	77491751	77269891
EDNRB	rs8000788	SNP_A-1839290	C	T	13	77267625	77491751	77273814
EDNRB	rs8002271	SNP_A-1787471	T	C	13	77267625	77491751	77273922
EDNRB	rs765377	SNP_A-8450679	C	T	13	77267625	77491751	77275835
EDNRB	rs975739	SNP_A-2161568	T	G	13	77267625	77491751	77279147
EDNRB	rs975738	SNP_A-8564941	T	A	13	77267625	77491751	77279180
EDNRB	rs17778592	SNP_A-8487643	G	A	13	77267625	77491751	77282415
EDNRB	rs1766347	SNP_A-1900775	G	C	13	77267625	77491751	77285964
EDNRB	rs10507874	SNP_A-4286941	G	T	13	77267625	77491751	77290759
EDNRB	rs9600936	SNP_A-8638487	A	G	13	77267625	77491751	77294841
EDNRB	rs681020	SNP_A-4200517	C	T	13	77267625	77491751	77301196
EDNRB	rs11619200	SNP_A-8422942	A	G	13	77267625	77491751	77303500
EDNRB	---	SNP_A-1897966	T	C	13	77267625	77491751	77303519
EDNRB	rs9530697	SNP_A-8465871	T	C	13	77267625	77491751	77303611
EDNRB	rs615608	SNP_A-1865732	T	C	13	77267625	77491751	77303884
EDNRB	rs686365	SNP_A-8566456	A	G	13	77267625	77491751	77306656
EDNRB	rs659016	SNP_A-1940833	C	T	13	77267625	77491751	77306995
EDNRB	rs17068394	SNP_A-4205365	G	A	13	77267625	77491751	77307136
EDNRB	rs1041619	SNP_A-8390767	A	T	13	77267625	77491751	77307151
EDNRB	rs1041620	SNP_A-8505380	A	G	13	77267625	77491751	77307535
EDNRB	rs9318501	SNP_A-8565698	A	T	13	77267625	77491751	77309063
EDNRB	rs667085	SNP_A-1910433	G	A	13	77267625	77491751	77319359
EDNRB	rs7332622	SNP_A-8582514	A	C	13	77267625	77491751	77319464
EDNRB	rs1572091	SNP_A-4290244	T	G	13	77267625	77491751	77326919
EDNRB	rs657507	SNP_A-2223080	A	G	13	77267625	77491751	77327261
EDNRB	rs605393	SNP_A-2236403	A	G	13	77267625	77491751	77328080
EDNRB	rs1924932	SNP_A-2161645	C	T	13	77267625	77491751	77338541
EDNRB	rs4885488	SNP_A-2161648	A	G	13	77267625	77491751	77338577
EDNRB	rs2329042	SNP_A-8472685	A	C	13	77267625	77491751	77340328
EDNRB	rs1924929	SNP_A-8322052	G	A	13	77267625	77491751	77345375
EDNRB	rs17068445	SNP_A-8515261	G	A	13	77267625	77491751	77345704
EDNRB	rs17780066	SNP_A-8542405	C	T	13	77267625	77491751	77346091
EDNRB	rs7982763	SNP_A-8565721	A	T	13	77267625	77491751	77353942
EDNRB	rs9600945	SNP_A-8636621	T	A	13	77267625	77491751	77358690
EDNRB	rs9544627	SNP_A-2133597	G	A	13	77267625	77491751	77359009
EDNRB	rs7998027	SNP_A-2161667	A	C	13	77267625	77491751	77359832
EDNRB	rs4885489	SNP_A-4227778	T	C	13	77267625	77491751	77361850
EDNRB	rs4591023	SNP_A-2076818	T	C	13	77267625	77491751	77362844
EDNRB	rs7994913	SNP_A-4286220	C	A	13	77267625	77491751	77363098
EDNRB	rs12720206	SNP_A-1836209	T	C	13	77267625	77491751	77366161
EDNRB	rs4885491	SNP_A-2271251	A	G	13	77267625	77491751	77368351
EDNRB	rs3027096	SNP_A-8583284	C	T	13	77267625	77491751	77370845
EDNRB	rs12720201	SNP_A-8704599	C	T	13	77267625	77491751	77370929
EDNRB	rs12720199	SNP_A-2243291	A	G	13	77267625	77491751	77371331
EDNRB	rs5351	SNP_A-8455980	C	T	13	77267625	77491751	77373314
EDNRB	rs12720193	SNP_A-8307609	T	C	13	77267625	77491751	77373482
EDNRB	rs12720192	SNP_A-8689058	A	G	13	77267625	77491751	77374330
EDNRB	rs3027108	SNP_A-1805728	T	C	13	77267625	77491751	77375898
EDNRB	rs2147555	SNP_A-2212536	C	A	13	77267625	77491751	77377386
EDNRB	rs3027111	SNP_A-8681517	C	T	13	77267625	77491751	77379869
EDNRB	rs4885493	SNP_A-2189443	C	G	13	77267625	77491751	77381064
EDNRB	rs3027128	SNP_A-8704428	G	A	13	77267625	77491751	77383685
EDNRB	rs3027129	SNP_A-8661740	C	T	13	77267625	77491751	77384402

EDNRB	rs1886480	SNP_A-2219914	A	G	13	77267625	77491751	77387085
EDNRB	rs1886481	SNP_A-4203075	T	C	13	77267625	77491751	77387386
EDNRB	rs7333867	SNP_A-8352819	T	A	13	77267625	77491751	77389535
EDNRB	rs9544636	SNP_A-8365944	C	T	13	77267625	77491751	77397570
EDNRB	rs2329049	SNP_A-2068337	A	G	13	77267625	77491751	77402856
EDNRB	rs7998775	SNP_A-8428197	C	T	13	77267625	77491751	77420428
EDNRB	rs7319342	SNP_A-2213537	G	A	13	77267625	77491751	77421485
EDNRB	rs11838546	SNP_A-8513709	A	G	13	77267625	77491751	77429615
EDNRB	rs12720129	SNP_A-8356508	C	T	13	77267625	77491751	77429851
EDNRB	rs4884076	SNP_A-8682512	T	C	13	77267625	77491751	77439953
EDNRB	rs13378491	SNP_A-8365383	T	C	13	77267625	77491751	77440968
EDNRB	rs4885499	SNP_A-8447667	G	A	13	77267625	77491751	77441722
EDNRB	rs4884077	SNP_A-8640927	T	C	13	77267625	77491751	77441896
EDNRB	rs12720100	SNP_A-2227018	G	A	13	77267625	77491751	77448249
EDNRB	rs9544656	SNP_A-2053365	G	A	13	77267625	77491751	77448842
EDNRB	rs7997031	SNP_A-8533133	G	A	13	77267625	77491751	77460488
EDNRB	rs17068628	SNP_A-4199863	A	G	13	77267625	77491751	77460551
EDNRB	rs17068642	SNP_A-1826349	G	A	13	77267625	77491751	77461037
EDNRB	rs17068646	SNP_A-1896517	A	T	13	77267625	77491751	77461465
EDNRB	rs3027091	SNP_A-8474837	T	C	13	77267625	77491751	77463816
EDNRB	rs9530709	SNP_A-2161767	T	A	13	77267625	77491751	77466447
EDNRB	rs10507878	SNP_A-2161768	T	C	13	77267625	77491751	77466739
EDNRB	rs17068678	SNP_A-4286956	G	A	13	77267625	77491751	77466765
EDNRB	rs17068683	SNP_A-4298626	G	A	13	77267625	77491751	77466872
EDNRB	rs4885506	SNP_A-4252283	T	C	13	77267625	77491751	77467402
EDNRB	rs4885507	SNP_A-1798098	G	A	13	77267625	77491751	77468514
EDNRB	rs1330910	SNP_A-1851030	T	C	13	77267625	77491751	77480045
EDNRB	rs1886482	SNP_A-2161799	C	T	13	77267625	77491751	77480389
EDNRB	rs9565375	SNP_A-1944542	A	G	13	77267625	77491751	77487553
HCN2	rs7258832	SNP_A-8621528	G	T	19	440893	668157	445057
HCN2	rs4919910	SNP_A-2109351	T	C	19	440893	668157	460717
HCN2	rs8107361	SNP_A-1878513	A	G	19	440893	668157	489160
HCN2	rs2288951	SNP_A-1881148	A	G	19	440893	668157	489487
HCN2	rs2070800	SNP_A-8369407	C	T	19	440893	668157	498973
HCN2	rs4919850	SNP_A-2079593	G	A	19	440893	668157	507632
HCN2	rs4919852	SNP_A-1795586	T	C	19	440893	668157	513530
HCN2	rs10417186	SNP_A-8645769	C	A	19	440893	668157	517738
HCN2	rs12609053	SNP_A-1785183	G	C	19	440893	668157	526083
HCN2	rs2283573	SNP_A-4289846	T	C	19	440893	668157	530100
HCN2	rs3752158	SNP_A-2100084	G	C	19	440893	668157	558984
HCN2	rs9304924	SNP_A-8376681	G	C	19	440893	668157	581844
HCN2	rs12610384	SNP_A-2300094	G	A	19	440893	668157	585095
HCN2	rs6510868	SNP_A-2297657	C	T	19	440893	668157	606608
HCN2	rs3787004	SNP_A-8320019	T	C	19	440893	668157	608666
HCN2	rs2301742	SNP_A-2182099	T	G	19	440893	668157	626513
HCN2	rs12611332	SNP_A-8439526	A	G	19	440893	668157	637181
HCN4	rs2680346	SNP_A-8716206	A	C	15	71300988	71548230	71314823
HCN4	rs747873	SNP_A-8716209	T	C	15	71300988	71548230	71315990
HCN4	rs1871385	SNP_A-8529296	C	T	15	71300988	71548230	71316651
HCN4	rs2254390	SNP_A-8716948	G	A	15	71300988	71548230	71334769
HCN4	rs8024434	SNP_A-8408396	A	C	15	71300988	71548230	71334780
HCN4	rs3784794	SNP_A-1785180	C	G	15	71300988	71548230	71334882
HCN4	rs2251205	SNP_A-8698194	A	G	15	71300988	71548230	71338303
HCN4	rs10518997	SNP_A-2295986	G	A	15	71300988	71548230	71343997
HCN4	rs2660826	SNP_A-4232715	G	A	15	71300988	71548230	71344687
HCN4	rs16957732	SNP_A-8608161	A	G	15	71300988	71548230	71350030

HCN4	rs8027588	SNP_A-2270937	T	C	15	71300988	71548230	71352703
HCN4	rs10438294	SNP_A-1949260	C	T	15	71300988	71548230	71363706
HCN4	rs499098	SNP_A-2278676	C	T	15	71300988	71548230	71371837
HCN4	---	SNP_A-2029130	T	A	15	71300988	71548230	71373224
HCN4	rs2252725	SNP_A-4260737	C	T	15	71300988	71548230	71373320
HCN4	rs2292915	SNP_A-8714195	T	C	15	71300988	71548230	71377632
HCN4	rs533460	SNP_A-2172069	G	A	15	71300988	71548230	71378143
HCN4	rs12442755	SNP_A-8713888	T	G	15	71300988	71548230	71378276
HCN4	rs8040516	SNP_A-2243045	T	C	15	71300988	71548230	71385601
HCN4	rs531019	SNP_A-2254886	A	G	15	71300988	71548230	71385660
HCN4	rs494493	SNP_A-2116304	T	C	15	71300988	71548230	71407050
HCN4	rs498005	SNP_A-2294121	C	T	15	71300988	71548230	71407363
HCN4	rs488156	SNP_A-1843421	A	G	15	71300988	71548230	71412948
HCN4	rs3859015	SNP_A-8696926	A	G	15	71300988	71548230	71414023
HCN4	rs488784	SNP_A-8716323	T	C	15	71300988	71548230	71422414
HCN4	rs568760	SNP_A-8305343	C	T	15	71300988	71548230	71431449
HCN4	rs489913	SNP_A-8375354	C	T	15	71300988	71548230	71432872
HCN4	rs8024056	SNP_A-1837843	G	T	15	71300988	71548230	71443821
HCN4	rs8038766	SNP_A-2111935	A	G	15	71300988	71548230	71450367
HCN4	rs3940418	SNP_A-4260529	C	G	15	71300988	71548230	71458570
HCN4	rs6495063	SNP_A-8478150	G	T	15	71300988	71548230	71469761
HCN4	rs6495064	SNP_A-8530756	C	T	15	71300988	71548230	71469920
HCN4	rs13329478	SNP_A-8324229	G	A	15	71300988	71548230	71484160
HCN4	rs1863403	SNP_A-8509958	T	G	15	71300988	71548230	71489605
HCN4	rs13379778	SNP_A-2056540	C	T	15	71300988	71548230	71490673
HCN4	rs11072418	SNP_A-8486876	C	T	15	71300988	71548230	71491217
HCN4	rs16957885	SNP_A-2296082	A	G	15	71300988	71548230	71504998
HCN4	rs10468058	SNP_A-8397194	C	T	15	71300988	71548230	71505973
HCN4	rs11072419	SNP_A-2175173	G	A	15	71300988	71548230	71507542
HCN4	rs11072420	SNP_A-8495612	C	T	15	71300988	71548230	71507759
HCN4	rs10519002	SNP_A-8706048	A	C	15	71300988	71548230	71509758
HCN4	rs2415151	SNP_A-2185857	A	C	15	71300988	71548230	71510318
HCN4	rs8032265	SNP_A-8530399	G	A	15	71300988	71548230	71519626
HCN4	rs16957901	SNP_A-8427975	T	C	15	71300988	71548230	71520844
HCN4	rs1582742	SNP_A-4214371	C	T	15	71300988	71548230	71525138
HCN4	rs3889591	SNP_A-8560555	G	A	15	71300988	71548230	71537925
HCN4	rs16957918	SNP_A-8585040	A	C	15	71300988	71548230	71538518
HCN4	rs11638992	SNP_A-2178319	T	C	15	71300988	71548230	71543937
HCN4	rs8043384	SNP_A-8645001	A	G	15	71300988	71548230	71544268
INS	rs10732518	SNP_A-1811408	C	T	11	2006927	2239027	2010508
INS	rs12287619	SNP_A-8411690	G	A	11	2006927	2239027	2011077
INS	rs11042391	SNP_A-8377768	G	A	11	2006927	2239027	2020174
INS	rs4930030	SNP_A-2100090	T	C	11	2006927	2239027	2043796
INS	rs10840297	SNP_A-8503014	T	G	11	2006927	2239027	2044303
INS	rs4930031	SNP_A-4220347	T	C	11	2006927	2239027	2044487
INS	rs10840303	SNP_A-8313824	G	C	11	2006927	2239027	2049559
INS	rs6578975	SNP_A-4251724	G	A	11	2006927	2239027	2052375
INS	rs11600952	SNP_A-8587391	G	A	11	2006927	2239027	2057859
INS	rs9971488	SNP_A-1812010	A	T	11	2006927	2239027	2057893
INS	rs7949013	SNP_A-8614940	T	C	11	2006927	2239027	2058268
INS	rs6578977	SNP_A-8704084	G	A	11	2006927	2239027	2058380
INS	rs12289890	SNP_A-8692919	T	G	11	2006927	2239027	2061791
INS	rs12575785	SNP_A-8424174	C	T	11	2006927	2239027	2065370
INS	rs7932949	SNP_A-8295584	G	A	11	2006927	2239027	2068107
INS	rs7947758	SNP_A-1784086	G	T	11	2006927	2239027	2075695
INS	rs4929957	SNP_A-8624565	G	A	11	2006927	2239027	2084885

INS	rs6578985	SNP_A-4232586	A	G	11	2006927	2239027	2094715
INS	rs6578986	SNP_A-8452384	A	G	11	2006927	2239027	2094728
INS	rs6578987	SNP_A-8322400	T	C	11	2006927	2239027	2098162
INS	rs734351	SNP_A-2222924	A	G	11	2006927	2239027	2112789
INS	rs3213225	SNP_A-8693341	G	A	11	2006927	2239027	2113112
INS	rs3213221	SNP_A-4253966	G	C	11	2006927	2239027	2113620
INS	rs3213219	SNP_A-8307885	C	T	11	2006927	2239027	2113726
INS	rs3213218	SNP_A-1870619	T	A	11	2006927	2239027	2114097
INS	rs3741208	SNP_A-2313355	G	A	11	2006927	2239027	2126350
INS	rs10840442	SNP_A-8632218	T	C	11	2006927	2239027	2128498
INS	rs7924316	SNP_A-8601849	G	T	11	2006927	2239027	2130023
INS	rs3842770	SNP_A-8324314	A	G	11	2006927	2239027	2135246
INS	rs11564708	SNP_A-8598009	G	C	11	2006927	2239027	2162902
INS	rs11043069	SNP_A-8684857	T	C	11	2006927	2239027	2177228
INS	rs3934904	SNP_A-4302409	T	C	11	2006927	2239027	2177516
INS	rs12270978	SNP_A-8404707	C	G	11	2006927	2239027	2179391
INS	rs12292212	SNP_A-8593347	G	A	11	2006927	2239027	2179489
INS	rs7478680	SNP_A-1861510	C	T	11	2006927	2239027	2179936
INS	rs11043147	SNP_A-8357242	G	C	11	2006927	2239027	2190745
INS	rs11606404	SNP_A-2194124	G	A	11	2006927	2239027	2197791
INS	rs11021732	SNP_A-2151950	T	C	11	2006927	2239027	2197848
INS	rs10765826	SNP_A-2073153	A	G	11	2006927	2239027	2197897
INS	rs6578246	SNP_A-2101944	A	G	11	2006927	2239027	2201921
INS	rs12099223	SNP_A-8606697	G	A	11	2006927	2239027	2212139
INS	rs11564688	SNP_A-8621810	T	G	11	2006927	2239027	2212857
INS	rs7941969	SNP_A-8554844	C	T	11	2006927	2239027	2217119
INS	rs10831633	SNP_A-8545344	A	G	11	2006927	2239027	2224512
INS	rs11021918	SNP_A-2170907	T	A	11	2006927	2239027	2225766
INS	rs6578252	SNP_A-4242618	G	T	11	2006927	2239027	2226817
NPPA	rs6660106	SNP_A-8354850	T	G	1	11728356	11930989	11729438
NPPA	rs6677313	SNP_A-8434763	C	G	1	11728356	11930989	11732432
NPPA	rs11121821	SNP_A-2148321	A	G	1	11728356	11930989	11733431
NPPA	rs1133398	SNP_A-2147876	A	G	1	11728356	11930989	11737218
NPPA	rs6661241	SNP_A-8699971	T	A	1	11728356	11930989	11737951
NPPA	rs6700513	SNP_A-8409392	G	C	1	11728356	11930989	11738679
NPPA	rs4845881	SNP_A-8373857	A	G	1	11728356	11930989	11750906
NPPA	rs6667720	SNP_A-1781313	T	C	1	11728356	11930989	11754202
NPPA	rs11121828	SNP_A-4260556	G	A	1	11728356	11930989	11757041
NPPA	rs4846042	SNP_A-1904281	C	A	1	11728356	11930989	11759886
NPPA	rs4845882	SNP_A-1852165	A	G	1	11728356	11930989	11765754
NPPA	rs4846048	SNP_A-8331309	A	G	1	11728356	11930989	11768839
NPPA	rs4845884	SNP_A-4222556	G	A	1	11728356	11930989	11769034
NPPA	rs7535669	SNP_A-4283999	C	G	1	11728356	11930989	11776186
NPPA	rs4846051	SNP_A-8625679	A	G	1	11728356	11930989	11777044
NPPA	rs1801131	SNP_A-8699092	T	G	1	11728356	11930989	11777063
NPPA	rs12121543	SNP_A-8302865	C	A	1	11728356	11930989	11777258
NPPA	rs9651118	SNP_A-1827473	T	C	1	11728356	11930989	11784801
NPPA	rs17367504	SNP_A-2274478	A	G	1	11728356	11930989	11785365
NPPA	rs17037429	SNP_A-8469046	C	T	1	11728356	11930989	11796374
NPPA	rs12404124	SNP_A-4286085	T	G	1	11728356	11930989	11796456
NPPA	rs11121833	SNP_A-8511837	C	T	1	11728356	11930989	11798630
NPPA	rs198391	SNP_A-4249172	C	T	1	11728356	11930989	11799004
NPPA	rs2076001	SNP_A-2121430	A	G	1	11728356	11930989	11801854
NPPA	rs12567136	SNP_A-1782458	T	C	1	11728356	11930989	11806318
NPPA	rs2076003	SNP_A-4202691	T	C	1	11728356	11930989	11806734
NPPA	rs7537765	SNP_A-1999881	G	A	1	11728356	11930989	11809890

NPPA	rs198401	SNP_A-8302978	A	G	1	11728356	11930989	11810971
NPPA	rs535107	SNP_A-4218646	G	A	1	11728356	11930989	11812055
NPPA	rs2236797	SNP_A-2210541	T	C	1	11728356	11930989	11815237
NPPA	rs2075538	SNP_A-4201134	C	T	1	11728356	11930989	11819189
NPPA	rs198403	SNP_A-2212770	A	G	1	11728356	11930989	11819241
NPPA	rs198404	SNP_A-4195470	A	T	1	11728356	11930989	11819414
NPPA	rs2075539	SNP_A-1843925	A	G	1	11728356	11930989	11820345
NPPA	rs1023252	SNP_A-4251993	G	T	1	11728356	11930989	11821620
NPPA	rs198413	SNP_A-4294108	T	C	1	11728356	11930989	11823389
NPPA	rs198414	SNP_A-8325275	T	G	1	11728356	11930989	11823412
NPPA	rs5065	SNP_A-4245831	G	A	1	11728356	11930989	11828655
NPPA	rs5063	SNP_A-2116869	C	T	1	11728356	11930989	11830235
NPPA	rs17376426	SNP_A-2288453	C	T	1	11728356	11930989	11830754
NPPA	rs198372	SNP_A-8409051	G	A	1	11728356	11930989	11832101
NPPA	rs6694164	SNP_A-2213757	T	C	1	11728356	11930989	11832926
NPPA	rs198381	SNP_A-8452767	A	G	1	11728356	11930989	11838336
NPPA	rs12562819	SNP_A-1880203	A	G	1	11728356	11930989	11846614
NPPA	rs12562952	SNP_A-8712968	T	C	1	11728356	11930989	11849643
NPPA	rs1009592	SNP_A-8559208	C	G	1	11728356	11930989	11851301
NPPA	rs1009591	SNP_A-8477217	T	C	1	11728356	11930989	11851417
NPPA	rs11803049	SNP_A-8630274	A	G	1	11728356	11930989	11851482
NPPA	rs4846064	SNP_A-2116773	C	T	1	11728356	11930989	11857429
NPPA	rs2008113	SNP_A-8300332	G	A	1	11728356	11930989	11861453
NPPA	rs12741980	SNP_A-8656415	C	A	1	11728356	11930989	11862180
NPPA	rs11588551	SNP_A-4301119	T	C	1	11728356	11930989	11864523
NPPA	rs6667637	SNP_A-1898145	A	G	1	11728356	11930989	11870544
NPPA	rs2336377	SNP_A-2123595	A	G	1	11728356	11930989	11872557
NPPA	rs1321073	SNP_A-2217826	G	A	1	11728356	11930989	11874213
NPPA	rs34175640	SNP_A-1795741	C	T	1	11728356	11930989	11874799
NPPA	rs17346334	SNP_A-4258689	C	T	1	11728356	11930989	11882981
NPPA	rs11800086	SNP_A-1804370	A	G	1	11728356	11930989	11894336
NPPA	rs744754	SNP_A-8402971	C	T	1	11728356	11930989	11930790
PTH	rs7116636	SNP_A-1916205	T	A	11	13370178	13574143	13386397
PTH	rs7103068	SNP_A-2218220	C	G	11	13370178	13574143	13386537
PTH	rs7122965	SNP_A-8597146	C	T	11	13370178	13574143	13386566
PTH	rs7925736	SNP_A-2139925	A	G	11	13370178	13574143	13393557
PTH	rs11022813	SNP_A-2156614	T	A	11	13370178	13574143	13399354
PTH	rs3789325	SNP_A-2193241	T	C	11	13370178	13574143	13399826
PTH	rs730415	SNP_A-8530617	C	T	11	13370178	13574143	13415321
PTH	rs10832036	SNP_A-1882183	C	A	11	13370178	13574143	13419429
PTH	rs7107468	SNP_A-4207234	A	C	11	13370178	13574143	13421460
PTH	rs307207	SNP_A-8333862	A	G	11	13370178	13574143	13432301
PTH	rs2593579	SNP_A-1837324	A	C	11	13370178	13574143	13435415
PTH	rs307246	SNP_A-1831582	A	G	11	13370178	13574143	13439803
PTH	rs307237	SNP_A-8333863	C	T	11	13370178	13574143	13450629
PTH	rs307238	SNP_A-4269210	A	G	11	13370178	13574143	13451340
PTH	rs12418886	SNP_A-2218929	T	C	11	13370178	13574143	13457354
PTH	rs307253	SNP_A-8333864	G	A	11	13370178	13574143	13466193
PTH	rs6254	SNP_A-8333865	T	C	11	13370178	13574143	13470839
PTH	rs16912882	SNP_A-8561899	T	C	11	13370178	13574143	13477575
PTH	rs12292991	SNP_A-8449569	G	A	11	13370178	13574143	13478596
PTH	rs10766086	SNP_A-1804810	C	T	11	13370178	13574143	13480027
PTH	rs11600801	SNP_A-2135172	T	C	11	13370178	13574143	13482815
PTH	rs11022858	SNP_A-2279314	C	T	11	13370178	13574143	13483209
PTH	rs307223	SNP_A-2037913	T	A	11	13370178	13574143	13490926
PTH	rs11022863	SNP_A-8644065	T	C	11	13370178	13574143	13495920

PTH	rs7930198	SNP_A-8571122	T	C	11	13370178	13574143	13503073
PTH	rs11605533	SNP_A-1797443	G	A	11	13370178	13574143	13503597
PTH	rs7931255	SNP_A-2188281	A	C	11	13370178	13574143	13504193
PTH	rs16912909	SNP_A-2184607	T	C	11	13370178	13574143	13504574
PTH	rs12365099	SNP_A-2090272	A	G	11	13370178	13574143	13504676
PTH	rs7101533	SNP_A-8543542	T	C	11	13370178	13574143	13506766
PTH	rs10832054	SNP_A-4222167	G	T	11	13370178	13574143	13506796
PTH	rs11022876	SNP_A-2086891	T	C	11	13370178	13574143	13529284
PTH	rs1459011	SNP_A-2007910	C	T	11	13370178	13574143	13530130
PTH	rs10500783	SNP_A-4269212	C	T	11	13370178	13574143	13530190
PTH	rs10500784	SNP_A-4269213	C	A	11	13370178	13574143	13530401
PTH	rs10500785	SNP_A-2007911	T	G	11	13370178	13574143	13530459
PTH	rs6486132	SNP_A-1914184	G	A	11	13370178	13574143	13540048
PTH	rs10500786	SNP_A-2007913	G	A	11	13370178	13574143	13540360
PTH	rs2018354	SNP_A-2007914	G	T	11	13370178	13574143	13540442
PTH	rs11022887	SNP_A-1855584	T	A	11	13370178	13574143	13541352
PTH	rs11022887	AFFX-SNP_11173969	T	A	11	13370178	13574143	13541352
PTH	rs1979877	SNP_A-4269214	A	G	11	13370178	13574143	13542274
PTH	rs1870411	SNP_A-2073019	G	A	11	13370178	13574143	13545519
PTH	rs11022889	SNP_A-2082100	A	G	11	13370178	13574143	13545586
PTH	rs1870413	SNP_A-1840039	T	G	11	13370178	13574143	13545731
PTH	rs898148	SNP_A-1823396	C	G	11	13370178	13574143	13554992
PTH	rs10734216	SNP_A-8513040	A	G	11	13370178	13574143	13564965
PTH	rs10766101	SNP_A-8558350	A	G	11	13370178	13574143	13573557
REN	rs2366007	SNP_A-8665989	C	G	1	202290571	202502088	202296226
REN	rs17404274	SNP_A-1980731	T	C	1	202290571	202502088	202299198
REN	rs7524385	SNP_A-2195213	A	G	1	202290571	202502088	202302170
REN	rs6696187	SNP_A-8711879	C	T	1	202290571	202502088	202307026
REN	rs4951300	SNP_A-8608138	A	G	1	202290571	202502088	202311189
REN	rs17404301	SNP_A-2077588	A	G	1	202290571	202502088	202314143
REN	rs11240658	SNP_A-8529153	C	T	1	202290571	202502088	202314510
REN	rs885330	SNP_A-8585491	G	A	1	202290571	202502088	202315716
REN	rs885332	SNP_A-1872112	T	G	1	202290571	202502088	202315811
REN	rs12117788	SNP_A-8684536	A	G	1	202290571	202502088	202315823
REN	rs12069284	SNP_A-8691374	A	G	1	202290571	202502088	202323744
REN	rs17404350	SNP_A-2114409	G	C	1	202290571	202502088	202323765
REN	rs7528560	SNP_A-8649552	G	A	1	202290571	202502088	202328367
REN	rs11240663	SNP_A-1980737	G	A	1	202290571	202502088	202328809
REN	rs12034028	SNP_A-8665991	C	T	1	202290571	202502088	202329031
REN	rs16852826	SNP_A-8703227	G	A	1	202290571	202502088	202334733
REN	rs3795579	SNP_A-1936277	T	G	1	202290571	202502088	202336461
REN	rs1534097	SNP_A-2186848	T	C	1	202290571	202502088	202336581
REN	rs11240666	SNP_A-8593194	G	A	1	202290571	202502088	202336847
REN	rs6700051	SNP_A-4264765	G	C	1	202290571	202502088	202338513
REN	rs16852855	SNP_A-1902774	G	C	1	202290571	202502088	202340845
REN	rs12563446	SNP_A-8354218	G	A	1	202290571	202502088	202341155
REN	rs4951043	SNP_A-2277203	T	C	1	202290571	202502088	202341488
REN	rs7549807	SNP_A-8317358	C	T	1	202290571	202502088	202345891
REN	rs940654	SNP_A-8665997	A	C	1	202290571	202502088	202347819
REN	rs2250538	SNP_A-1960774	C	T	1	202290571	202502088	202348515
REN	rs2293336	SNP_A-1980744	C	T	1	202290571	202502088	202348625
REN	rs2037679	SNP_A-1980745	C	T	1	202290571	202502088	202349561
REN	rs16852893	SNP_A-8344367	T	A	1	202290571	202502088	202354616
REN	rs12092337	SNP_A-2179125	T	A	1	202290571	202502088	202359648
REN	rs12085092	SNP_A-8425963	G	A	1	202290571	202502088	202359745
REN	rs2975631	SNP_A-8411646	A	T	1	202290571	202502088	202360112

REN	rs16852933	SNP_A-2109931	C	T	1	202290571	202502088	202362101
REN	rs12730254	SNP_A-8478353	A	G	1	202290571	202502088	202368044
REN	rs6679960	SNP_A-2268291	A	T	1	202290571	202502088	202371427
REN	rs16852953	SNP_A-1843824	A	T	1	202290571	202502088	202371798
REN	rs10900553	SNP_A-8325067	C	T	1	202290571	202502088	202374786
REN	rs4951307	SNP_A-2293103	G	A	1	202290571	202502088	202375116
REN	rs3737656	SNP_A-2314805	T	C	1	202290571	202502088	202377283
REN	rs1106778	SNP_A-8685833	C	T	1	202290571	202502088	202382171
REN	rs940651	SNP_A-8489688	C	G	1	202290571	202502088	202382209
REN	rs4951310	SNP_A-8305981	A	G	1	202290571	202502088	202384433
REN	rs1997034	SNP_A-1880165	T	C	1	202290571	202502088	202384983
REN	rs11571093	SNP_A-2253389	C	G	1	202290571	202502088	202390294
REN	rs2887284	SNP_A-2191870	C	A	1	202290571	202502088	202391271
REN	rs2368564	SNP_A-1980757	C	T	1	202290571	202502088	202391488
REN	rs3795575	SNP_A-8666007	G	A	1	202290571	202502088	202391712
REN	rs11571085	SNP_A-8380764	T	C	1	202290571	202502088	202394896
REN	rs11571116	SNP_A-8707288	A	G	1	202290571	202502088	202395460
REN	rs1464816	SNP_A-8717306	T	G	1	202290571	202502088	202395477
REN	rs5707	SNP_A-8307625	C	A	1	202290571	202502088	202396294
REN	rs7521667	SNP_A-8313532	G	T	1	202290571	202502088	202396750
REN	rs11571082	SNP_A-1914440	T	C	1	202290571	202502088	202397654
REN	rs11571080	SNP_A-8409826	T	C	1	202290571	202502088	202398254
REN	rs11571103	SNP_A-1905016	A	G	1	202290571	202502088	202398834
REN	rs10900555	SNP_A-2256856	A	G	1	202290571	202502088	202398933
REN	rs6693954	SNP_A-2113906	T	A	1	202290571	202502088	202399261
REN	rs11571078	SNP_A-8485287	G	A	1	202290571	202502088	202399797
REN	rs11240688	SNP_A-8389780	G	A	1	202290571	202502088	202401145
REN	rs16853055	SNP_A-8686217	G	T	1	202290571	202502088	202405925
REN	rs16853059	SNP_A-2260631	T	C	1	202290571	202502088	202406807
REN	rs16853062	SNP_A-2166246	A	T	1	202290571	202502088	202406841
REN	rs10494849	SNP_A-8666010	C	T	1	202290571	202502088	202410750
REN	rs7548714	SNP_A-8362994	T	C	1	202290571	202502088	202410872
REN	rs7513165	SNP_A-8679800	C	T	1	202290571	202502088	202413809
REN	rs10900558	SNP_A-2216394	C	T	1	202290571	202502088	202416146
REN	rs4951315	SNP_A-2093294	A	C	1	202290571	202502088	202423791
REN	rs12040249	SNP_A-8470045	G	A	1	202290571	202502088	202424851
REN	rs7524621	SNP_A-8677748	A	G	1	202290571	202502088	202427561
REN	rs7538038	SNP_A-2201887	G	A	1	202290571	202502088	202427649
REN	rs4951316	SNP_A-1807034	C	T	1	202290571	202502088	202428113
REN	rs12097666	SNP_A-4256358	T	C	1	202290571	202502088	202428835
REN	rs4951319	SNP_A-2155201	T	C	1	202290571	202502088	202428853
REN	rs12568353	SNP_A-8447148	A	G	1	202290571	202502088	202431314
REN	rs3924587	SNP_A-2313894	C	T	1	202290571	202502088	202432177
REN	rs9726776	SNP_A-8531854	G	A	1	202290571	202502088	202440010
REN	rs4373805	SNP_A-2192820	A	G	1	202290571	202502088	202442202
REN	rs6685338	SNP_A-4255017	T	C	1	202290571	202502088	202442351
REN	rs11240698	SNP_A-2123384	T	A	1	202290571	202502088	202443577
REN	rs10900560	SNP_A-8531843	T	A	1	202290571	202502088	202447249
REN	rs4534452	SNP_A-1980774	A	G	1	202290571	202502088	202451807
REN	rs4631764	SNP_A-2050370	T	C	1	202290571	202502088	202457131
REN	rs7546241	SNP_A-8527196	A	G	1	202290571	202502088	202460397
REN	rs17333220	SNP_A-8537227	A	T	1	202290571	202502088	202460414
REN	rs11240700	SNP_A-4271353	C	T	1	202290571	202502088	202462579
REN	rs12026655	SNP_A-8424337	T	C	1	202290571	202502088	202462643
REN	rs11240701	SNP_A-2247119	A	G	1	202290571	202502088	202462670
REN	rs4581328	SNP_A-4276728	C	A	1	202290571	202502088	202462950

REN	rs4614318	SNP_A-1785674	C	T	1	202290571	202502088	202463105
REN	rs16853122	SNP_A-8425216	G	A	1	202290571	202502088	202463279
REN	rs12062034	SNP_A-8471963	A	G	1	202290571	202502088	202463430
REN	rs12760824	SNP_A-8645136	T	C	1	202290571	202502088	202464248
REN	rs6665623	SNP_A-8695484	A	G	1	202290571	202502088	202466633
REN	rs6683388	SNP_A-2151149	T	G	1	202290571	202502088	202466883
REN	rs6679037	SNP_A-4213967	T	C	1	202290571	202502088	202467898
REN	rs4434894	SNP_A-2257929	A	C	1	202290571	202502088	202470274
REN	rs6702751	SNP_A-1820712	C	A	1	202290571	202502088	202478416
REN	rs12031222	SNP_A-2270673	G	C	1	202290571	202502088	202480337
REN	rs4951327	SNP_A-2046028	A	C	1	202290571	202502088	202480981
REN	rs4951329	SNP_A-8561500	T	C	1	202290571	202502088	202483033
REN	rs4245724	SNP_A-1906205	C	T	1	202290571	202502088	202483385
REN	rs4951054	SNP_A-8682847	G	A	1	202290571	202502088	202483769
REN	rs10900568	SNP_A-2095195	C	T	1	202290571	202502088	202486855
REN	rs4453105	SNP_A-8704880	G	A	1	202290571	202502088	202486985
REN	rs11808198	SNP_A-8466331	T	C	1	202290571	202502088	202490807
REN	rs4951338	SNP_A-4236424	G	A	1	202290571	202502088	202496675
REN	rs4338423	SNP_A-8415424	T	C	1	202290571	202502088	202497787
REN	rs10751446	SNP_A-4201677	C	T	1	202290571	202502088	202498123
REN	rs4951056	SNP_A-8553137	T	C	1	202290571	202502088	202499595
REN	rs6675149	SNP_A-8666024	A	G	1	202290571	202502088	202500399
