# Supplementary material for: Computational Analysis of Candidate Disease Genes and Variants for Salt-Sensitive Hypertension in Indigenous Southern Africans
Source: PLoS One. 2010 Sep 27;5(9):e12989. doi: 10.1371/journal.pone.0012989 (PMC2946338; doi:10.1371/journal.pone.0012989)
Supplement: Data File S3 — Final Scoring matrix for all genes. (0.08 MB PDF) [file pone.0012989.s003.pdf]

Supplementary data file S3. Final Scoring matrix for all genes

ensembl_id	adrenergic_sympathetic	atrial_natriuretic_peptide	dopamine_receptor	renin_angiotensin_aldosterone	sodium_transport	sodium_reabsorption_kidney	(sodium_reabsorption)_kidney	(sodium_excretion)_kidney	sodium_excretion_kidney	low_renin	aldosterone_levels	salt_sensitivity	hypertension_associated	Total
ENSG00000152266														6
ENSG00000144891														6
ENSG00000151617														5
ENSG00000135744														5
ENSG00000143839														5
ENSG00000107937														4
ENSG00000138622														4
ENSG00000136160														4
ENSG00000099822														4
ENSG00000214274														4
ENSG00000124721														4
ENSG00000175206														4
ENSG00000129965														4
ENSG00000163631														4
ENSG00000137992														4
ENSG00000197956														4
ENSG00000100359														4
ENSG00000159640														4
ENSG00000162711														4
ENSG00000078401														4
ENSG00000180772														3.5
ENSG00000007171														3.5
ENSG00000198873														3
ENSG00000215782														3
ENSG00000121807														3
ENSG00000128271														3
ENSG00000091428														3
ENSG00000082556														3
ENSG00000183134														3
ENSG00000185149														3
ENSG00000166148														3
ENSG00000171596														3
ENSG00000197405														3
ENSG00000138669														3
ENSG00000185532														3
ENSG00000005249														3
ENSG00000170289														3
ENSG00000164588														3
ENSG00000143630														3
ENSG00000136237														3
ENSG00000164047														3
ENSG00000101200														3
ENSG00000174827														3

ENSG00000113889	3
ENSG00000076685	3
ENSG00000185813	3
ENSG00000000971	3
ENSG00000120937	3
ENSG00000171873	2.5
ENSG00000115263	2.5
ENSG00000080293	2.5
ENSG00000110680	2.5
ENSG00000189162	2.5
ENSG00000197408	2.5
ENSG00000147571	2.5
ENSG00000197561	2.5
ENSG00000105516	2.5
ENSG00000115138	2.5
ENSG00000141404	2
ENSG00000166833	2
ENSG00000156052	2
ENSG00000087460	2
ENSG00000114353	2
ENSG00000060558	2
ENSG00000049540	2
ENSG00000087258	2
ENSG00000065135	2
ENSG00000185974	2
ENSG00000100077	2
ENSG00000125388	2
ENSG00000173020	2
ENSG00000153283	2
ENSG00000198055	2
ENSG00000114124	2
ENSG00000175658	2
ENSG00000185321	2
ENSG00000215778	2
ENSG00000215775	2
ENSG00000215644	2
ENSG00000158748	2
ENSG00000204918	2
ENSG00000204917	2
ENSG00000204916	2
ENSG00000204914	2
ENSG00000179546	2
ENSG00000099869	2
ENSG00000205928	2
ENSG00000133105	2
ENSG00000206282	2
ENSG00000099910	2
ENSG00000205107	2
ENSG00000182090	2
ENSG00000168329	2
ENSG00000181773	2
ENSG00000158195	2
ENSG00000206210	2
ENSG00000121797	2
ENSG00000169403	2
ENSG00000148734	2

ENSG00000116329	2
ENSG00000103495	2
ENSG00000171509	2
ENSG00000173368	2
ENSG00000121764	2
ENSG00000102468	2
ENSG00000205432	2
ENSG00000139679	2
ENSG00000180561	2
ENSG00000120088	2
ENSG00000198933	2
ENSG00000126010	2
ENSG00000166603	2
ENSG00000184303	2
ENSG00000050767	2
ENSG00000204919	2
ENSG00000186998	2
ENSG00000182157	2
ENSG00000112038	2
ENSG00000166573	2
ENSG00000178110	2
ENSG00000169676	2
ENSG00000174600	2
ENSG00000165621	2
ENSG00000120907	2
ENSG00000069696	2
ENSG00000182631	2
ENSG00000169508	2
ENSG00000196277	2
ENSG00000128313	2
ENSG00000171659	2
ENSG00000137414	2
ENSG00000171522	2
ENSG00000164850	2
ENSG00000111321	2
ENSG00000128310	2
ENSG00000105663	2
ENSG00000110148	2
ENSG00000213906	2
ENSG00000213903	2
ENSG00000204681	2
ENSG00000068308	2
ENSG00000178394	2
ENSG00000072195	2
ENSG00000128272	2
ENSG00000128285	2
ENSG00000171860	2
ENSG00000183813	2
ENSG00000182223	2
ENSG00000121335	2
ENSG00000179934	2
ENSG00000106113	2
ENSG00000164220	2
ENSG00000164251	2
ENSG00000106128	2
ENSG00000065325	2

ENSG00000144648	2
ENSG00000171201	2
ENSG00000056291	2
ENSG00000186912	2
ENSG00000173585	2
ENSG00000172215	2
ENSG00000173578	2
ENSG00000099864	2
ENSG00000163823	2
ENSG00000183625	2
ENSG00000160791	2
ENSG00000170425	2
ENSG00000108557	2
ENSG00000169429	2
ENSG00000186810	2
ENSG00000204174	2
ENSG00000213694	2
ENSG00000138756	2
ENSG00000173198	2
ENSG00000147145	2
ENSG00000078589	2
ENSG00000188779	2
ENSG00000187140	2
ENSG00000129911	2
ENSG00000134817	2
ENSG00000165617	2
ENSG00000182759	2
ENSG00000125910	2
ENSG00000204218	2
ENSG00000136928	2
ENSG00000050628	2
ENSG00000169951	2
ENSG00000164082	2
ENSG00000125510	2
ENSG00000124493	2
ENSG00000160973	2
ENSG00000198121	2
ENSG00000147246	2
ENSG00000198822	2
ENSG00000196739	2
ENSG00000181291	2
ENSG00000004948	2
ENSG00000125730	2
ENSG00000112164	2
ENSG00000164270	2
ENSG00000169252	2
ENSG00000180535	2
ENSG00000179097	2
ENSG00000154165	2
ENSG00000102239	2
ENSG00000171475	2
ENSG00000126353	2
ENSG00000170989	2
ENSG00000170214	2
ENSG00000166856	2
ENSG00000151577	2

ENSG00000164128	2
ENSG00000184845	2
ENSG00000113749	2
ENSG00000126895	2
ENSG00000165409	2
ENSG00000175591	2
ENSG00000140030	2
ENSG00000119714	2
ENSG00000170775	2
ENSG00000179603	2
ENSG00000129048	2
ENSG00000150594	2
ENSG00000181072	2
ENSG00000168398	2
ENSG00000149295	2
ENSG00000043591	2
ENSG00000121966	2
ENSG00000198851	2
ENSG00000119973	2
ENSG00000174944	2
ENSG00000169313	2
ENSG00000107281	2
ENSG00000168830	2
ENSG00000169860	2
ENSG00000160683	2
ENSG00000180616	2
ENSG00000183484	2
ENSG00000090539	2
ENSG00000182687	2
ENSG00000176170	2
ENSG00000146399	2
ENSG00000064989	2
ENSG00000173080	2
ENSG00000064547	2
ENSG00000131016	2
ENSG00000183671	2
ENSG00000130368	2
ENSG00000180871	2
ENSG00000163464	2
ENSG00000112486	2
ENSG00000196218	2
ENSG00000187994	2
ENSG00000054356	2
ENSG00000130669	2
ENSG00000144476	2
ENSG00000170128	2
ENSG00000142273	2
ENSG00000130201	2
ENSG00000177464	2
ENSG00000198049	2
ENSG00000134830	2
ENSG00000171051	2
ENSG00000142408	2
ENSG00000181210	2
ENSG00000182559	2
ENSG00000196712	2

ENSG00000113389	**2**
ENSG00000183943	**2**
ENSG00000132356	**2**
ENSG00000165059	**2**
ENSG00000181929	**2**
ENSG00000142875	**2**
ENSG00000072062	**2**
ENSG00000215677	**2**
ENSG00000198515	**2**
ENSG00000188191	**2**
ENSG00000132259	**2**
ENSG00000147081	**2**
ENSG00000114302	**2**
ENSG00000079337	**2**
ENSG00000138784	**2**
ENSG00000032444	**2**
ENSG00000183862	**2**
ENSG00000144191	**2**
ENSG00000131791	**2**
ENSG00000108946	**2**
ENSG00000168734	**2**
ENSG00000171033	**2**
ENSG00000135549	**2**
ENSG00000215855	**2**
ENSG00000154040	**2**
ENSG00000169245	**2**
ENSG00000145491	**2**
ENSG00000065371	**2**
ENSG00000114547	**2**
ENSG00000064199	**2**
ENSG00000138193	**2**
ENSG00000176387	**2**
ENSG00000023843	**2**
ENSG00000140992	**2**
ENSG00000171759	**2**
ENSG00000198931	**2**
ENSG00000147889	**2**
ENSG00000167580	**2**
ENSG00000174738	**2**
ENSG00000166710	**2**
ENSG00000089250	**2**
ENSG00000205403	**2**
ENSG00000152670	**2**
ENSG00000006062	**2**
ENSG00000160226	**2**
ENSG00000141342	**2**
ENSG00000165078	**2**
ENSG00000174697	**2**
ENSG00000196549	**2**
ENSG00000135318	**2**
ENSG00000198467	**2**
ENSG00000148926	**2**
ENSG00000179142	**2**
ENSG00000138792	**2**
ENSG00000127955	**1.5**
ENSG00000036828	**1.5**

ENSG00000163485	**1.5**
ENSG00000100031	**1.5**
ENSG00000066230	**1.5**
ENSG00000132932	**1.5**
ENSG00000124813	**1.5**
ENSG00000180210	**1.5**
ENSG00000143199	**1.5**
ENSG00000074803	**1.5**
ENSG00000153767	**1.5**
ENSG00000197406	**1.5**
ENSG00000108784	**1.5**
ENSG00000070915	**1.5**
ENSG00000035928	**1.5**
ENSG00000073756	**1.5**
ENSG00000106258	**1.5**
ENSG00000121691	**1.5**
ENSG00000163956	**1.5**
ENSG00000165471	**1.5**
ENSG00000215522	**1.5**
ENSG00000137332	**1.5**
ENSG00000204580	**1.5**
ENSG00000188610	**1**
ENSG00000132698	**1**
ENSG00000197959	**1**
ENSG00000215823	**1**
ENSG00000215814	**1**
ENSG00000215813	**1**
ENSG00000215801	**1**
ENSG00000116688	**1**
ENSG00000132881	**1**
ENSG00000215634	**1**
ENSG00000206492	**1**
ENSG00000173213	**1**
ENSG00000206412	**1**
ENSG00000206418	**1**
ENSG00000168461	**1**
ENSG00000183311	**1**
ENSG00000137379	**1**
ENSG00000161149	**1**
ENSG00000215451	**1**
ENSG00000149656	**1**
ENSG00000088387	**1**
ENSG00000070831	**1**
ENSG00000198033	**1**
ENSG00000125780	**1**
ENSG00000178605	**1**
ENSG00000139890	**1**
ENSG00000166922	**1**
ENSG00000176014	**1**
ENSG00000183785	**1**
ENSG00000122035	**1**
ENSG00000120555	**1**
ENSG00000196503	**1**
ENSG00000185739	**1**
ENSG00000215093	**1**
ENSG00000170590	**1**

ENSG00000185305	[redacted]			1
ENSG00000174225	[redacted]			1
ENSG00000206004	[redacted]			1
ENSG00000103342	[redacted]			1
ENSG00000184702	[redacted]			1
ENSG00000215032	[redacted]			1
ENSG00000152784	[redacted]			1
ENSG00000122872	[redacted]			1
ENSG00000176276	[redacted]			1
ENSG00000164740	[redacted]			1
ENSG00000128482	[redacted]			1
ENSG00000214834	[redacted]			1
ENSG00000122545	[redacted]			1
ENSG00000106608	[redacted]			1
ENSG00000112090	[redacted]			1
ENSG00000188060	[redacted]			1
ENSG00000214765	[redacted]			1
ENSG00000180574	[redacted]			1
ENSG00000214744	[redacted]			1
ENSG00000164402	[redacted]			1
ENSG00000184743	[redacted]			1
ENSG00000152214	[redacted]			1
ENSG00000205922	[redacted]			1
ENSG00000188447	[redacted]			1
ENSG00000088538	[redacted]			1
ENSG00000188144	[redacted]			1
ENSG00000214622	[redacted]			1
ENSG00000140598	[redacted]			1
ENSG00000127589	[redacted]			1
ENSG00000183486	[redacted]			1
ENSG00000157601	[redacted]			1
ENSG00000214576	[redacted]			1
ENSG00000185051	[redacted]			1
ENSG00000141956	[redacted]			1
ENSG00000141748	[redacted]			1
ENSG00000128266	[redacted]			1
ENSG00000100228	[redacted]			1
ENSG00000205242	[redacted]			1
ENSG00000138069	[redacted]			1
ENSG00000165178	[redacted]			1
ENSG00000214492	[redacted]			1
ENSG00000123595	[redacted]			1
ENSG00000147324	[redacted]			1
ENSG00000041353	[redacted]			1
ENSG00000196642	[redacted]			1
ENSG00000214415	[redacted]			1
ENSG00000214405	[redacted]			1
ENSG00000185829	[redacted]			1
ENSG00000154237	[redacted]			1
ENSG00000214391	[redacted]			1
ENSG00000136447	[redacted]			1
ENSG00000152213	[redacted]			1
ENSG00000088320	[redacted]			1
ENSG00000150316	[redacted]			1
ENSG00000088325	[redacted]			1
ENSG00000159247	[redacted]			1

ENSG00000107099	[redacted]			**1**
ENSG00000197183	[redacted]			**1**
ENSG00000173876	[redacted]			**1**
ENSG00000100276	[redacted]			**1**
ENSG00000158417	[redacted]			**1**
ENSG00000130158	[redacted]			**1**
ENSG00000136155	[redacted]			**1**
ENSG00000147853	[redacted]			**1**
ENSG00000120805	[redacted]			**1**
ENSG00000129472	[redacted]			**1**
ENSG00000157869	[redacted]			**1**
ENSG00000186522	[redacted]			**1**
ENSG00000008853	[redacted]			**1**
ENSG00000137267	[redacted]			**1**
ENSG00000197562	[redacted]			**1**
ENSG00000137285	[redacted]			**1**
ENSG00000134697	[redacted]			**1**
ENSG00000140983	[redacted]			**1**
ENSG00000184640	[redacted]			**1**
ENSG00000130741	[redacted]			**1**
ENSG00000174775	[redacted]			**1**
ENSG00000157741	[redacted]			**1**
ENSG00000125249	[redacted]			**1**
ENSG00000116954	[redacted]			**1**
ENSG00000185721	[redacted]			**1**
ENSG00000111737	[redacted]			**1**
ENSG00000141519	[redacted]			**1**
ENSG00000214087	[redacted]			**1**
ENSG00000155876	[redacted]			**1**
ENSG00000104140	[redacted]			**1**
ENSG00000134108	[redacted]			**1**
ENSG00000178462	[redacted]			**1**
ENSG00000100302	[redacted]			**1**
ENSG00000103966	[redacted]			**1**
ENSG00000133612	[redacted]			**1**
ENSG00000124795	[redacted]			**1**
ENSG00000214005	[redacted]			**1**
ENSG00000139832	[redacted]			**1**
ENSG00000100360	[redacted]			**1**
ENSG00000004478	[redacted]			**1**
ENSG00000167964	[redacted]			**1**
ENSG00000131653	[redacted]			**1**
ENSG00000128340	[redacted]			**1**
ENSG00000198959	[redacted]			**1**
ENSG00000100889	[redacted]			**1**
ENSG00000177105	[redacted]			**1**
ENSG00000146535	[redacted]			**1**
ENSG00000086475	[redacted]			**1**
ENSG00000112812	[redacted]			**1**
ENSG00000100226	[redacted]			**1**
ENSG00000187682	[redacted]			**1**
ENSG00000132254	[redacted]			**1**
ENSG00000152932	[redacted]			**1**
ENSG00000136238	[redacted]			**1**
ENSG00000163526	[redacted]			**1**
ENSG00000068400	[redacted]			**1**

ENSG00000160007				1
ENSG00000144566				1
ENSG00000140623				1
ENSG00000104388				1
ENSG00000197885				1
ENSG00000113595				1
ENSG00000122644				1
ENSG00000168421				1
ENSG00000135905				1
ENSG00000049769				1
ENSG00000100852				1
ENSG00000165997				1
ENSG00000151806				1
ENSG00000144635				1
ENSG00000204590				1
ENSG00000189369				1
ENSG00000188042				1
ENSG00000131409				1
ENSG00000100883				1
ENSG00000100167				1
ENSG00000196230				1
ENSG00000133818				1
ENSG00000128045				1
ENSG00000069974				1
ENSG00000164347				1
ENSG00000099246				1
ENSG00000084092				1
ENSG00000134533				1
ENSG00000134333				1
ENSG00000130119				1
ENSG00000166796				1
ENSG00000166800				1
ENSG00000111404				1
ENSG00000164949				1
ENSG00000083750				1
ENSG00000111716				1
ENSG00000171989				1
ENSG00000156049				1
ENSG00000169213				1
ENSG00000104450				1
ENSG00000165105				1
ENSG00000133703				1
ENSG00000147127				1
ENSG00000006451				1
ENSG00000143878				1
ENSG00000165527				1
ENSG00000198513				1
ENSG00000115137				1
ENSG00000124253				1
ENSG00000166128				1
ENSG00000100503				1
ENSG00000124209				1
ENSG00000084733				1
ENSG00000108551				1
ENSG00000087470				1
ENSG00000108591				1

ENSG00000103710				**1**
ENSG00000101162				**1**
ENSG00000138758				**1**
ENSG00000188906				**1**
ENSG00000165023				**1**
ENSG00000103769				**1**
ENSG00000079974				**1**
ENSG00000101181				**1**
ENSG00000178952				**1**
ENSG00000132434				**1**
ENSG00000154997				**1**
ENSG00000116641				**1**
ENSG00000115254				**1**
ENSG00000067560				**1**
ENSG00000162433				**1**
ENSG00000013016				**1**
ENSG00000072422				**1**
ENSG00000109113				**1**
ENSG00000101210				**1**
ENSG00000176490				**1**
ENSG00000162595				**1**
ENSG00000172602				**1**
ENSG00000134287				**1**
ENSG00000114349				**1**
ENSG00000167550				**1**
ENSG00000088256				**1**
ENSG00000123416				**1**
ENSG00000101246				**1**
ENSG00000180096				**1**
ENSG00000167552				**1**
ENSG00000172409				**1**
ENSG00000119787				**1**
ENSG00000167553				**1**
ENSG00000102128				**1**
ENSG00000172476				**1**
ENSG00000079332				**1**
ENSG00000167658				**1**
ENSG00000123570				**1**
ENSG00000152700				**1**
ENSG00000126785				**1**
ENSG00000138036				**1**
ENSG00000177733				**1**
ENSG00000132591				**1**
ENSG00000137959				**1**
ENSG00000137965				**1**
ENSG00000119729				**1**
ENSG00000163938				**1**
ENSG00000126858				**1**
ENSG00000147251				**1**
ENSG00000117226				**1**
ENSG00000117228				**1**
ENSG00000139998				**1**
ENSG00000125354				**1**
ENSG00000162645				**1**
ENSG00000213512				**1**
ENSG00000105793				**1**

ENSG00000162654				**1**
ENSG00000154451				**1**
ENSG00000168374				**1**
ENSG00000183347				**1**
ENSG00000085760				**1**
ENSG00000141150				**1**
ENSG00000104833				**1**
ENSG00000148672				**1**
ENSG00000151276				**1**
ENSG00000134594				**1**
ENSG00000119396				**1**
ENSG00000110047				**1**
ENSG00000124615				**1**
ENSG00000213465				**1**
ENSG00000123728				**1**
ENSG00000169379				**1**
ENSG00000172007				**1**
ENSG00000113966				**1**
ENSG00000111540				**1**
ENSG00000132911				**1**
ENSG00000174903				**1**
ENSG00000166592				**1**
ENSG00000114993				**1**
ENSG00000171403				**1**
ENSG00000185236				**1**
ENSG00000173156				**1**
ENSG00000172432				**1**
ENSG00000168256				**1**
ENSG00000119718				**1**
ENSG00000163541				**1**
ENSG00000108774				**1**
ENSG00000163607				**1**
ENSG00000204764				**1**
ENSG00000128581				**1**
ENSG00000131462				**1**
ENSG00000037042				**1**
ENSG00000175414				**1**
ENSG00000145439				**1**
ENSG00000135439				**1**
ENSG00000108830				**1**
ENSG00000134183				**1**
ENSG00000144840				**1**
ENSG00000198496				**1**
ENSG00000175906				**1**
ENSG00000169228				**1**
ENSG00000106976				**1**
ENSG00000175582				**1**
ENSG00000127314				**1**
ENSG00000079805				**1**
ENSG00000108883				**1**
ENSG00000172992				**1**
ENSG00000155961				**1**
ENSG00000116473				**1**
ENSG00000105514				**1**
ENSG00000112210				**1**
ENSG00000155366				**1**

ENSG00000137502				1
ENSG00000132394				1
ENSG00000166197				1
ENSG00000075785				1
ENSG00000080371				1
ENSG00000123892				1
ENSG00000004059				1
ENSG00000172780				1
ENSG00000138175				1
ENSG00000213281				1
ENSG00000156508				1
ENSG00000144134				1
ENSG00000144867				1
ENSG00000154917				1
ENSG00000179331				1
ENSG00000108387				1
ENSG00000144118				1
ENSG00000158186				1
ENSG00000198211				1
ENSG00000108423				1
ENSG00000146955				1
ENSG00000085265				1
ENSG00000152086				1
ENSG00000075886				1
ENSG00000087191				1
ENSG00000050327				1
ENSG00000171115				1
ENSG00000179144				1
ENSG00000133574				1
ENSG00000133561				1
ENSG00000181467				1
ENSG00000106560				1
ENSG00000213203				1
ENSG00000196329				1
ENSG00000115963				1
ENSG00000162980				1
ENSG00000167461				1
ENSG00000188229				1
ENSG00000025039				1
ENSG00000168827				1
ENSG00000106615				1
ENSG00000179674				1
ENSG00000120063				1
ENSG00000100664				1
ENSG00000130299				1
ENSG00000182934				1
ENSG00000171109				1
ENSG00000185100				1
ENSG00000172794				1
ENSG00000148824				1
ENSG00000138430				1
ENSG00000143545				1
ENSG00000074935				1
ENSG00000139725				1
ENSG00000182782				1
ENSG00000198836				1

ENSG00000041802			1
ENSG00000105649			1
ENSG00000163374			1
ENSG00000132341			1
ENSG00000143622			1
ENSG00000112339			1
ENSG00000169750			1
ENSG00000064933			1
ENSG00000141542			1
ENSG00000118508			1
ENSG00000127824			1
ENSG00000167578			1
ENSG00000157985			1
ENSG00000124839			1
ENSG00000168385			1
ENSG00000143862			1
ENSG00000124449			1
ENSG00000117280			1
ENSG00000054392			1
ENSG00000024422			1
ENSG00000143761			1
ENSG00000116574			1
ENSG00000168118			1
ENSG00000126458			1
ENSG00000035687			1
ENSG00000105492			1
ENSG00000175704			1
ENSG00000204826			1
ENSG00000204782			1
ENSG00000185637			1
ENSG00000183940			1
ENSG00000133375			1
ENSG00000204650			1
ENSG00000173386			1
ENSG00000116219			1
ENSG00000204105			1
ENSG00000162618			1
ENSG00000211451			1
ENSG00000159450			1
ENSG00000203758			1
ENSG00000180437			1
ENSG00000180409			1
ENSG00000198452			1
ENSG00000153230			1
ENSG00000177693			1
ENSG00000185097			1
ENSG00000169962			1
ENSG00000116151			1
ENSG00000158292			1
ENSG00000187017			1
ENSG00000173662			1
ENSG00000180758			1
ENSG00000206517			1
ENSG00000206516			1
ENSG00000112461			1
ENSG00000204692			1

ENSG00000206472				1
ENSG00000206471				1
ENSG00000179002				1
ENSG00000099972				1
ENSG00000125816				1
ENSG00000215535				1
ENSG00000134508				1
ENSG00000141431				1
ENSG00000206449				1
ENSG00000137315				1
ENSG00000096155				1
ENSG00000132205				1
ENSG00000196673				1
ENSG00000215385				1
ENSG00000196383				1
ENSG00000177257				1
ENSG00000206313				1
ENSG00000206014				1
ENSG00000185896				1
ENSG00000128739				1
ENSG00000197526				1
ENSG00000177400				1
ENSG00000188822				1
ENSG00000168484				1
ENSG00000133226				1
ENSG00000188459				1
ENSG00000130538				1
ENSG00000167949				1
ENSG00000156304				1
ENSG00000176136				1
ENSG00000185231				1
ENSG00000215211				1
ENSG00000180144				1
ENSG00000125861				1
ENSG00000132970				1
ENSG00000179443				1
ENSG00000132975				1
ENSG00000215167				1
ENSG00000150471				1
ENSG00000165556				1
ENSG00000215134				1
ENSG00000205710				1
ENSG00000089486				1
ENSG00000159128				1
ENSG00000205510				1
ENSG00000197674				1
ENSG00000197426				1
ENSG00000197023				1
ENSG00000183553				1
ENSG00000215080				1
ENSG00000197455				1
ENSG00000205409				1
ENSG00000163739				1
ENSG00000180947				1
ENSG00000188691				1
ENSG00000184523				1

ENSG00000176716	[redacted]			1
ENSG00000072952	[redacted]			1
ENSG00000163749	[redacted]			1
ENSG00000184113	[redacted]			1
ENSG00000111679	[redacted]			1
ENSG00000166963	[redacted]			1
ENSG00000184058	[redacted]			1
ENSG00000133149	[redacted]			1
ENSG00000185838	[redacted]			1
ENSG00000134489	[redacted]			1
ENSG00000141380	[redacted]			1
ENSG00000101292	[redacted]			1
ENSG00000185903	[redacted]			1
ENSG00000168126	[redacted]			1
ENSG00000204702	[redacted]			1
ENSG00000204697	[redacted]			1
ENSG00000159212	[redacted]			1
ENSG00000102195	[redacted]			1
ENSG00000109920	[redacted]			1
ENSG00000197161	[redacted]			1
ENSG00000214909	[redacted]			1
ENSG00000102080	[redacted]			1
ENSG00000187503	[redacted]			1
ENSG00000181943	[redacted]			1
ENSG00000214880	[redacted]			1
ENSG00000182242	[redacted]			1
ENSG00000185701	[redacted]			1
ENSG00000166693	[redacted]			1
ENSG00000204516	[redacted]			1
ENSG00000205025	[redacted]			1
ENSG00000197467	[redacted]			1
ENSG00000131981	[redacted]			1
ENSG00000188152	[redacted]			1
ENSG00000158161	[redacted]			1
ENSG00000188712	[redacted]			1
ENSG00000150907	[redacted]			1
ENSG00000180714	[redacted]			1
ENSG00000204989	[redacted]			1
ENSG00000181790	[redacted]			1
ENSG00000197870	[redacted]			1
ENSG00000183935	[redacted]			1
ENSG00000182162	[redacted]			1
ENSG00000169084	[redacted]			1
ENSG00000102780	[redacted]			1
ENSG00000002586	[redacted]			1
ENSG00000214662	[redacted]			1
ENSG00000124749	[redacted]			1
ENSG00000101850	[redacted]			1
ENSG00000214591	[redacted]			1
ENSG00000214582	[redacted]			1
ENSG00000176269	[redacted]			1
ENSG00000198678	[redacted]			1
ENSG00000188888	[redacted]			1
ENSG00000131504	[redacted]			1
ENSG00000090061	[redacted]			1
ENSG00000184055	[redacted]			1

ENSG00000179799				**1**
ENSG00000006638				**1**
ENSG00000164825				**1**
ENSG00000105278				**1**
ENSG00000177306				**1**
ENSG00000205847				**1**
ENSG00000159692				**1**
ENSG00000171711				**1**
ENSG00000119919				**1**
ENSG00000152207				**1**
ENSG00000177679				**1**
ENSG00000102539				**1**
ENSG00000196143				**1**
ENSG00000182652				**1**
ENSG00000176299				**1**
ENSG00000176294				**1**
ENSG00000169249				**1**
ENSG00000165762				**1**
ENSG00000176281				**1**
ENSG00000155249				**1**
ENSG00000205331				**1**
ENSG00000169488				**1**
ENSG00000169484				**1**
ENSG00000176253				**1**
ENSG00000176246				**1**
ENSG00000176230				**1**
ENSG00000184394				**1**
ENSG00000196832				**1**
ENSG00000176219				**1**
ENSG00000176198				**1**
ENSG00000088247				**1**
ENSG00000214344				**1**
ENSG00000183444				**1**
ENSG00000179170				**1**
ENSG00000121904				**1**
ENSG00000135540				**1**
ENSG00000168992				**1**
ENSG00000186629				**1**
ENSG00000183706				**1**
ENSG00000182974				**1**
ENSG00000178336				**1**
ENSG00000183248				**1**
ENSG00000181803				**1**
ENSG00000166225				**1**
ENSG00000116560				**1**
ENSG00000112419				**1**
ENSG00000131142				**1**
ENSG00000214251				**1**
ENSG00000105061				**1**
ENSG00000174948				**1**
ENSG00000174930				**1**
ENSG00000164488				**1**
ENSG00000181214				**1**
ENSG00000196703				**1**
ENSG00000197849				**1**
ENSG00000165804				**1**

ENSG00000169327				1
ENSG00000116871				1
ENSG00000131061				1
ENSG00000076344				1
ENSG00000184984				1
ENSG00000155269				1
ENSG00000169777				1
ENSG00000139746				1
ENSG00000169208				1
ENSG00000169202				1
ENSG00000108370				1
ENSG00000185187				1
ENSG00000145147				1
ENSG00000175509				1
ENSG00000125631				1
ENSG00000166073				1
ENSG00000111707				1
ENSG00000167281				1
ENSG00000127588				1
ENSG00000162009				1
ENSG00000125245				1
ENSG00000163394				1
ENSG00000198173				1
ENSG00000176923				1
ENSG00000185666				1
ENSG00000104290				1
ENSG00000180914				1
ENSG00000010017				1
ENSG00000179420				1
ENSG00000176510				1
ENSG00000170356				1
ENSG00000050393				1
ENSG00000157827				1
ENSG00000133619				1
ENSG00000171657				1
ENSG00000183016				1
ENSG00000179862				1
ENSG00000188778				1
ENSG00000129691				1
ENSG00000184166				1
ENSG00000183024				1
ENSG00000172150				1
ENSG00000163703				1
ENSG00000172146				1
ENSG00000180090				1
ENSG00000166159				1
ENSG00000180068				1
ENSG00000180016				1
ENSG00000159961				1
ENSG00000127780				1
ENSG00000053702				1
ENSG00000107262				1
ENSG00000078246				1
ENSG00000157017				1
ENSG00000183473				1
ENSG00000164849				1

ENSG00000148426			1
ENSG00000196639			1
ENSG00000008056			1
ENSG00000126767			1
ENSG00000181963			1
ENSG00000196778			1
ENSG00000197790			1
ENSG00000175609			1
ENSG00000197428			1
ENSG00000180785			1
ENSG00000167332			1
ENSG00000188069			1
ENSG00000176937			1
ENSG00000176925			1
ENSG00000176922			1
ENSG00000176900			1
ENSG00000083168			1
ENSG00000176895			1
ENSG00000176893			1
ENSG00000176879			1
ENSG00000205497			1
ENSG00000205496			1
ENSG00000176798			1
ENSG00000205495			1
ENSG00000176787			1
ENSG00000205494			1
ENSG00000171944			1
ENSG00000182070			1
ENSG00000176742			1
ENSG00000183251			1
ENSG00000184881			1
ENSG00000176239			1
ENSG00000167355			1
ENSG00000167360			1
ENSG00000167359			1
ENSG00000187918			1
ENSG00000181609			1
ENSG00000181616			1
ENSG00000168124			1
ENSG00000168131			1
ENSG00000124657			1
ENSG00000124191			1
ENSG00000168158			1
ENSG00000015285			1
ENSG00000181023			1
ENSG00000181009			1
ENSG00000181001			1
ENSG00000180988			1
ENSG00000183269			1
ENSG00000180974			1
ENSG00000183389			1
ENSG00000180934			1
ENSG00000180919			1
ENSG00000175485			1
ENSG00000184574			1
ENSG00000166311			1

ENSG00000204704			**1**
ENSG00000179921			**1**
ENSG00000204703			**1**
ENSG00000112462			**1**
ENSG00000204695			**1**
ENSG00000183729			**1**
ENSG00000168787			**1**
ENSG00000206474			**1**
ENSG00000204694			**1**
ENSG00000204688			**1**
ENSG00000204687			**1**
ENSG00000148481			**1**
ENSG00000110811			**1**
ENSG00000168140			**1**
ENSG00000188124			**1**
ENSG00000170803			**1**
ENSG00000184933			**1**
ENSG00000111664			**1**
ENSG00000166363			**1**
ENSG00000170790			**1**
ENSG00000170782			**1**
ENSG00000166368			**1**
ENSG00000178358			**1**
ENSG00000204657			**1**
ENSG00000140274			**1**
ENSG00000183303			**1**
ENSG00000182334			**1**
ENSG00000175393			**1**
ENSG00000170683			**1**
ENSG00000137098			**1**
ENSG00000168828			**1**
ENSG00000122718			**1**
ENSG00000164651			**1**
ENSG00000046889			**1**
ENSG00000136235			**1**
ENSG00000204646			**1**
ENSG00000104043			**1**
ENSG00000182083			**1**
ENSG00000168267			**1**
ENSG00000184194			**1**
ENSG00000145216			**1**
ENSG00000151025			**1**
ENSG00000121377			**1**
ENSG00000121314			**1**
ENSG00000121381			**1**
ENSG00000121318			**1**
ENSG00000212128			**1**
ENSG00000134551			**1**
ENSG00000212127			**1**
ENSG00000212126			**1**
ENSG00000212125			**1**
ENSG00000212124			**1**
ENSG00000186136			**1**
ENSG00000105997			**1**
ENSG00000069966			**1**
ENSG00000183150			**1**

ENSG00000136754			1
ENSG00000013588			1
ENSG00000111291			1
ENSG00000179094			1
ENSG00000139874			1
ENSG00000179826			1
ENSG00000179817			1
ENSG00000084463			1
ENSG00000181104			1
ENSG00000078549			1
ENSG00000187550			1
ENSG00000109163			1
ENSG00000170255			1
ENSG00000114812			1
ENSG00000183695			1
ENSG00000167191			1
ENSG00000180269			1
ENSG00000169006			1
ENSG00000187258			1
ENSG00000176695			1
ENSG00000204612			1
ENSG00000109208			1
ENSG00000204463			1
ENSG00000187037			1
ENSG00000038427			1
ENSG00000158813			1
ENSG00000205213			1
ENSG00000106536			1
ENSG00000124089			1
ENSG00000178015			1
ENSG00000177283			1
ENSG00000164930			1
ENSG00000143006			1
ENSG00000107562			1
ENSG00000174417			1
ENSG00000116014			1
ENSG00000172678			1
ENSG00000072310			1
ENSG00000163734			1
ENSG00000081041			1
ENSG00000186469			1
ENSG00000188603			1
ENSG00000084693			1
ENSG00000138755			1
ENSG00000168229			1
ENSG00000125384			1
ENSG00000136286			1
ENSG00000136960			1
ENSG00000164054			1
ENSG00000163792			1
ENSG00000099625			1
ENSG00000087495			1
ENSG00000163794			1
ENSG00000156787			1
ENSG00000130699			1
ENSG00000101180			1

ENSG00000071626				1
ENSG00000175216				1
ENSG00000147138				1
ENSG00000168672				1
ENSG00000168175				1
ENSG00000168944				1
ENSG00000101188				1
ENSG00000092758				1
ENSG00000167371				1
ENSG00000101190				1
ENSG00000149658				1
ENSG00000000003				1
ENSG00000213611				1
ENSG00000175619				1
ENSG00000172208				1
ENSG00000176567				1
ENSG00000176555				1
ENSG00000176547				1
ENSG00000205046				1
ENSG00000172640				1
ENSG00000197376				1
ENSG00000142864				1
ENSG00000188763				1
ENSG00000186419				1
ENSG00000178306				1
ENSG00000176529				1
ENSG00000185926				1
ENSG00000204882				1
ENSG00000181961				1
ENSG00000181958				1
ENSG00000181939				1
ENSG00000181935				1
ENSG00000172188				1
ENSG00000181927				1
ENSG00000174982				1
ENSG00000181903				1
ENSG00000198877				1
ENSG00000172380				1
ENSG00000186113				1
ENSG00000186117				1
ENSG00000186119				1
ENSG00000205030				1
ENSG00000205029				1
ENSG00000187612				1
ENSG00000167825				1
ENSG00000174970				1
ENSG00000149133				1
ENSG00000181785				1
ENSG00000172154				1
ENSG00000181767				1
ENSG00000181761				1
ENSG00000167822				1
ENSG00000181752				1
ENSG00000174957				1
ENSG00000181718				1
ENSG00000172489				1

ENSG00000181698			1
ENSG00000181693			1
ENSG00000181689			1
ENSG00000150261			1
ENSG00000172487			1
ENSG00000174942			1
ENSG00000150269			1
ENSG00000174937			1
ENSG00000181371			1
ENSG00000172464			1
ENSG00000172459			1
ENSG00000174914			1
ENSG00000172457			1
ENSG00000181273			1
ENSG00000183908			1
ENSG00000169221			1
ENSG00000186907			1
ENSG00000181619			1
ENSG00000139970			1
ENSG00000186509			1
ENSG00000172381			1
ENSG00000172377			1
ENSG00000186513			1
ENSG00000197887			1
ENSG00000172774			1
ENSG00000180475			1
ENSG00000172772			1
ENSG00000197786			1
ENSG00000172769			1
ENSG00000172365			1
ENSG00000172362			1
ENSG00000198283			1
ENSG00000178719			1
ENSG00000075073			1
ENSG00000176495			1
ENSG00000172324			1
ENSG00000172320			1
ENSG00000166884			1
ENSG00000176200			1
ENSG00000172742			1
ENSG00000172289			1
ENSG00000171700			1
ENSG00000081059			1
ENSG00000180929			1
ENSG00000125522			1
ENSG00000110844			1
ENSG00000149534			1
ENSG00000096395			1
ENSG00000099381			1
ENSG00000137309			1
ENSG00000186881			1
ENSG00000148136			1
ENSG00000204246			1
ENSG00000186943			1
ENSG00000204245			1
ENSG00000179074			1

ENSG00000136839				**1**
ENSG00000179055				**1**
ENSG00000162881				**1**
ENSG00000160949				**1**
ENSG00000133131				**1**
ENSG00000167987				**1**
ENSG00000148143				**1**
ENSG00000122420				**1**
ENSG00000169758				**1**
ENSG00000117114				**1**
ENSG00000138279				**1**
ENSG00000120738				**1**
ENSG00000135180				**1**
ENSG00000143933				**1**
ENSG00000109320				**1**
ENSG00000174021				**1**
ENSG00000171133				**1**
ENSG00000184261				**1**
ENSG00000171517				**1**
ENSG00000059769				**1**
ENSG00000169836				**1**
ENSG00000120306				**1**
ENSG00000138039				**1**
ENSG00000170820				**1**
ENSG00000131725				**1**
ENSG00000162188				**1**
ENSG00000213523				**1**
ENSG00000119737				**1**
ENSG00000119431				**1**
ENSG00000010244				**1**
ENSG00000168539				**1**
ENSG00000180245				**1**
ENSG00000157240				**1**
ENSG00000167771				**1**
ENSG00000180316				**1**
ENSG00000106952				**1**
ENSG00000006075				**1**
ENSG00000148604				**1**
ENSG00000107771				**1**
ENSG00000122375				**1**
ENSG00000113649				**1**
ENSG00000186867				**1**
ENSG00000127928				**1**
ENSG00000106804				**1**
ENSG00000127920				**1**
ENSG00000173250				**1**
ENSG00000168066				**1**
ENSG00000102970				**1**
ENSG00000205352				**1**
ENSG00000174837				**1**
ENSG00000147262				**1**
ENSG00000198369				**1**
ENSG00000171054				**1**
ENSG00000169618				**1**
ENSG00000139572				**1**
ENSG00000162298				**1**

ENSG00000136834			**1**
ENSG00000170837			**1**
ENSG00000197233			**1**
ENSG00000213457			**1**
ENSG00000171505			**1**
ENSG00000171501			**1**
ENSG00000171496			**1**
ENSG00000165202			**1**
ENSG00000171484			**1**
ENSG00000173679			**1**
ENSG00000171481			**1**
ENSG00000136939			**1**
ENSG00000171459			**1**
ENSG00000148215			**1**
ENSG00000165204			**1**
ENSG00000148680			**1**
ENSG00000167258			**1**
ENSG00000164040			**1**
ENSG00000135413			**1**
ENSG00000170605			**1**
ENSG00000179919			**1**
ENSG00000197706			**1**
ENSG00000188324			**1**
ENSG00000205330			**1**
ENSG00000205329			**1**
ENSG00000187857			**1**
ENSG00000205328			**1**
ENSG00000185821			**1**
ENSG00000179695			**1**
ENSG00000184954			**1**
ENSG00000205327			**1**
ENSG00000182732			**1**
ENSG00000179626			**1**
ENSG00000188394			**1**
ENSG00000175398			**1**
ENSG00000107864			**1**
ENSG00000196578			**1**
ENSG00000197036			**1**
ENSG00000206537			**1**
ENSG00000198068			**1**
ENSG00000197621			**1**
ENSG00000197938			**1**
ENSG00000196098			**1**
ENSG00000206536			**1**
ENSG00000181828			**1**
ENSG00000144810			**1**
ENSG00000186188			**1**
ENSG00000108352			**1**
ENSG00000176607			**1**
ENSG00000165370			**1**
ENSG00000131759			**1**
ENSG00000101977			**1**
ENSG00000139641			**1**
ENSG00000184140			**1**
ENSG00000182854			**1**
ENSG00000186092			**1**

ENSG00000181656				1
ENSG00000166925				1
ENSG00000146216				1
ENSG00000173621				1
ENSG00000172354				1
ENSG00000115353				1
ENSG00000181733				1
ENSG00000170929				1
ENSG00000170923				1
ENSG00000161807				1
ENSG00000170920				1
ENSG00000188000				1
ENSG00000174667				1
ENSG00000175514				1
ENSG00000184347				1
ENSG00000188038				1
ENSG00000187122				1
ENSG00000121957				1
ENSG00000130810				1
ENSG00000157606				1
ENSG00000145414				1
ENSG00000164129				1
ENSG00000172938				1
ENSG00000172935				1
ENSG00000166987				1
ENSG00000184451				1
ENSG00000213380				1
ENSG00000175898				1
ENSG00000153292				1
ENSG00000100583				1
ENSG00000175697				1
ENSG00000156097				1
ENSG00000124818				1
ENSG00000102076				1
ENSG00000147380				1
ENSG00000166160				1
ENSG00000185254				1
ENSG00000131096				1
ENSG00000186635				1
ENSG00000180739				1
ENSG00000105851				1
ENSG00000168597				1
ENSG00000172209				1
ENSG00000171631				1
ENSG00000180340				1
ENSG00000137252				1
ENSG00000164342				1
ENSG00000171561				1
ENSG00000164604				1
ENSG00000121933				1
ENSG00000168412				1
ENSG00000135272				1
ENSG00000113262				1
ENSG00000050820				1
ENSG00000198668				1
ENSG00000182742				1

ENSG00000128519	[redacted]			**1**
ENSG00000135973	[redacted]			**1**
ENSG00000106299	[redacted]			**1**
ENSG00000167083	[redacted]			**1**
ENSG00000174339	[redacted]			**1**
ENSG00000139292	[redacted]			**1**
ENSG00000182613	[redacted]			**1**
ENSG00000174804	[redacted]			**1**
ENSG00000168959	[redacted]			**1**
ENSG00000183127	[redacted]			**1**
ENSG00000173401	[redacted]			**1**
ENSG00000134640	[redacted]			**1**
ENSG00000136457	[redacted]			**1**
ENSG00000128617	[redacted]			**1**
ENSG00000163914	[redacted]			**1**
ENSG00000123901	[redacted]			**1**
ENSG00000128602	[redacted]			**1**
ENSG00000134200	[redacted]			**1**
ENSG00000138172	[redacted]			**1**
ENSG00000017260	[redacted]			**1**
ENSG00000167513	[redacted]			**1**
ENSG00000129993	[redacted]			**1**
ENSG00000153951	[redacted]			**1**
ENSG00000091527	[redacted]			**1**
ENSG00000072071	[redacted]			**1**
ENSG00000123146	[redacted]			**1**
ENSG00000160951	[redacted]			**1**
ENSG00000123159	[redacted]			**1**
ENSG00000051382	[redacted]			**1**
ENSG00000100739	[redacted]			**1**
ENSG00000144230	[redacted]			**1**
ENSG00000127362	[redacted]			**1**
ENSG00000127364	[redacted]			**1**
ENSG00000127366	[redacted]			**1**
ENSG00000136715	[redacted]			**1**
ENSG00000183609	[redacted]			**1**
ENSG00000131355	[redacted]			**1**
ENSG00000135312	[redacted]			**1**
ENSG00000127507	[redacted]			**1**
ENSG00000173302	[redacted]			**1**
ENSG00000127530	[redacted]			**1**
ENSG00000188269	[redacted]			**1**
ENSG00000127515	[redacted]			**1**
ENSG00000185385	[redacted]			**1**
ENSG00000127529	[redacted]			**1**
ENSG00000183840	[redacted]			**1**
ENSG00000179468	[redacted]			**1**
ENSG00000196405	[redacted]			**1**
ENSG00000107518	[redacted]			**1**
ENSG00000094661	[redacted]			**1**
ENSG00000143368	[redacted]			**1**
ENSG00000185899	[redacted]			**1**
ENSG00000187164	[redacted]			**1**
ENSG00000213215	[redacted]			**1**
ENSG00000141867	[redacted]			**1**
ENSG00000174946	[redacted]			**1**

ENSG00000138271			1
ENSG00000181631			1
ENSG00000198829			1
ENSG00000171942			1
ENSG00000171936			1
ENSG00000172519			1
ENSG00000186723			1
ENSG00000136026			1
ENSG00000118432			1
ENSG00000176231			1
ENSG00000136542			1
ENSG00000033100			1
ENSG00000112218			1
ENSG00000154478			1
ENSG00000157219			1
ENSG00000196099			1
ENSG00000181518			1
ENSG00000171014			1
ENSG00000181499			1
ENSG00000196248			1
ENSG00000181457			1
ENSG00000184863			1
ENSG00000204299			1
ENSG00000127533			1
ENSG00000204298			1
ENSG00000182634			1
ENSG00000196341			1
ENSG00000197263			1
ENSG00000204293			1
ENSG00000196661			1
ENSG00000198657			1
ENSG00000197125			1
ENSG00000170953			1
ENSG00000196119			1
ENSG00000175029			1
ENSG00000171634			1
ENSG00000154146			1
ENSG00000152034			1
ENSG00000182263			1
ENSG00000173890			1
ENSG00000106018			1
ENSG00000121853			1
ENSG00000186766			1
ENSG00000159516			1
ENSG00000196169			1
ENSG00000084234			1
ENSG00000170412			1
ENSG00000108046			1
ENSG00000197177			1
ENSG00000142208			1
ENSG00000146360			1
ENSG00000135144			1
ENSG00000161204			1
ENSG00000135111			1
ENSG00000155130			1
ENSG00000128652			1

ENSG00000143595				1
ENSG00000123500				1
ENSG00000160712				1
ENSG00000173612				1
ENSG00000066651				1
ENSG00000157005				1
ENSG00000196917				1
ENSG00000130511				1
ENSG00000180658				1
ENSG00000146385				1
ENSG00000146383				1
ENSG00000135569				1
ENSG00000146378				1
ENSG00000111432				1
ENSG00000105662				1
ENSG00000168542				1
ENSG00000181408				1
ENSG00000135577				1
ENSG00000152822				1
ENSG00000132694				1
ENSG00000186306				1
ENSG00000180708				1
ENSG00000173285				1
ENSG00000198965				1
ENSG00000197532				1
ENSG00000186400				1
ENSG00000198967				1
ENSG00000196171				1
ENSG00000203757				1
ENSG00000180433				1
ENSG00000197403				1
ENSG00000188340				1
ENSG00000155760				1
ENSG00000213088				1
ENSG00000196266				1
ENSG00000196184				1
ENSG00000184155				1
ENSG00000138443				1
ENSG00000126266				1
ENSG00000185897				1
ENSG00000126251				1
ENSG00000126262				1
ENSG00000161249				1
ENSG00000163251				1
ENSG00000146469				1
ENSG00000126254				1
ENSG00000186517				1
ENSG00000115365				1
ENSG00000132185				1
ENSG00000162746				1
ENSG00000120436				1
ENSG00000143147				1
ENSG00000117479				1
ENSG00000117560				1
ENSG00000203737				1
ENSG00000135898				1

ENSG00000135914			**1**
ENSG00000143324			**1**
ENSG00000077312			**1**
ENSG00000116690			**1**
ENSG00000116703			**1**
ENSG00000127074			**1**
ENSG00000178623			**1**
ENSG00000143355			**1**
ENSG00000079385			**1**
ENSG00000170075			**1**
ENSG00000133067			**1**
ENSG00000104859			**1**
ENSG00000125753			**1**
ENSG00000010310			**1**
ENSG00000125755			**1**
ENSG00000160014			**1**
ENSG00000160013			**1**
ENSG00000167414			**1**
ENSG00000082482			**1**
ENSG00000136630			**1**
ENSG00000154380			**1**
ENSG00000168243			**1**
ENSG00000126461			**1**
ENSG00000133019			**1**
ENSG00000182901			**1**
ENSG00000054277			**1**
ENSG00000104973			**1**
ENSG00000204673			**1**
ENSG00000177535			**1**
ENSG00000203664			**1**
ENSG00000196242			**1**
ENSG00000177489			**1**
ENSG00000177476			**1**
ENSG00000197437			**1**
ENSG00000169214			**1**
ENSG00000196772			**1**
ENSG00000169136			**1**
ENSG00000197591			**1**
ENSG00000198281			**1**
ENSG00000177462			**1**
ENSG00000196071			**1**
ENSG00000196936			**1**
ENSG00000187080			**1**
ENSG00000203663			**1**
ENSG00000198128			**1**
ENSG00000162727			**1**
ENSG00000198601			**1**
ENSG00000177233			**1**
ENSG00000171180			**1**
ENSG00000177212			**1**
ENSG00000177201			**1**
ENSG00000177186			**1**
ENSG00000177174			**1**
ENSG00000196944			**1**
ENSG00000198104			**1**
ENSG00000175143			**1**

ENSG00000196240				**1**
ENSG00000196539				**1**
ENSG00000203661				**1**
ENSG00000188558				**1**
ENSG00000183310				**1**
ENSG00000184022				**1**
ENSG00000183130				**1**
ENSG00000177151				**1**
ENSG00000187701				**1**
ENSG00000189181				**1**
ENSG00000142511				**1**
ENSG00000142544				**1**
ENSG00000105509				**1**
ENSG00000171049				**1**
ENSG00000187474				**1**
ENSG00000196131				**1**
ENSG00000174677				**1**
ENSG00000167615				**1**
ENSG00000167617				**1**
ENSG00000131037				**1**
ENSG00000178201				**1**
ENSG00000180467				**1**
ENSG00000136832				**1**
ENSG00000198371				**1**
ENSG00000212937				**1**
ENSG00000186445				**1**
ENSG00000176312				**1**
ENSG00000176213				**1**
ENSG00000212931				**1**
ENSG00000172611				**1**
ENSG00000198703				**1**
ENSG00000186440				**1**
ENSG00000158731				**1**
ENSG00000175180				**1**
ENSG00000198118				**1**
ENSG00000196079				**1**
ENSG00000177275				**1**
ENSG00000197454				**1**
ENSG00000203662				**1**
ENSG00000182783				**1**
ENSG00000206525				**1**
ENSG00000206524				**1**
ENSG00000206522				**1**
ENSG00000206523				**1**
ENSG00000206519				**1**
ENSG00000206515				**1**
ENSG00000206512				**1**
ENSG00000124732				**1**
ENSG00000112453				**1**
ENSG00000196906				**1**
ENSG00000196231				**1**
ENSG00000112459				**1**
ENSG00000204690				**1**
ENSG00000204689				**1**
ENSG00000206470				**1**
ENSG00000206467				**1**

ENSG00000179266				1
ENSG00000179880				1
ENSG00000212821				1
ENSG00000212820				1
ENSG00000212819				1
ENSG00000179257				1
ENSG00000212818				1
ENSG00000212817				1
ENSG00000212816				1
ENSG00000173525				1
ENSG00000212814				1
ENSG00000212813				1
ENSG00000212812				1
ENSG00000212811				1
ENSG00000212810				1
ENSG00000212809				1
ENSG00000212808				1
ENSG00000212807				1
ENSG00000211458				1
ENSG00000212806				1
ENSG00000168113				1
ENSG00000204701				1
ENSG00000204700				1
ENSG00000204698				1
ENSG00000204686				1
ENSG00000172764				1
ENSG00000182170				1
ENSG00000184350				1
ENSG00000174541				1
ENSG00000176904				1
ENSG00000176748				1
ENSG00000184698				1
ENSG00000184321				1
ENSG00000187747				1
ENSG00000181074				1
ENSG00000184478				1
ENSG00000183313				1
ENSG00000180923				1
ENSG00000180913				1
ENSG00000180909				1
ENSG00000166408				1
ENSG00000170688				1
ENSG00000176540				1
ENSG00000182565				1
ENSG00000176537				1
ENSG00000181950				1
ENSG00000188439				1
ENSG00000186886				1
ENSG00000181837				1
ENSG00000186124				1
ENSG00000184741				1
ENSG00000181780				1
ENSG00000181723				1
ENSG00000172199				1
ENSG00000184878				1
ENSG00000205026				1

ENSG00000181296	1
ENSG00000181282	1
ENSG00000196779	1
ENSG00000185408	1
ENSG00000182743	1
ENSG00000182625	1
ENSG00000183909	1
ENSG00000205797	1
ENSG00000133136	1
ENSG00000165312	1
ENSG00000204332	1
ENSG00000182880	1
ENSG00000205813	1
ENSG00000132671	1
ENSG00000198261	1
ENSG00000198674	1
ENSG00000186268	1
ENSG00000196403	1
ENSG00000197309	1
ENSG00000204294	1
ENSG00000198317	1
ENSG00000178586	1
ENSG00000188314	1
ENSG00000172148	1
ENSG00000176938	1
ENSG00000205292	1
ENSG00000177799	1
ENSG00000198760	1
ENSG00000141194	1
ENSG00000170374	1
ENSG00000196534	1
ENSG00000205332	1
ENSG00000179615	1
ENSG00000184899	1
ENSG00000159279	1
ENSG00000107485	1
ENSG00000146013	1
ENSG00000109062	1
ENSG00000169418	1
ENSG00000172367	1
ENSG00000183751	1
ENSG00000168824	1
ENSG00000170091	1
ENSG00000130643	1
ENSG00000173175	1
ENSG00000178473	1
ENSG00000118702	1
ENSG00000087494	1
ENSG00000213705	1
ENSG00000144407	1
ENSG00000175279	1
ENSG00000090104	1
ENSG00000206511	1
ENSG00000206466	1
ENSG00000131236	1
ENSG00000112186	1

ENSG00000137841	1
ENSG00000171777	1
ENSG00000146648	1
ENSG00000149527	1
ENSG00000197421	1
ENSG00000182621	1
ENSG00000101333	1
ENSG00000182378	1
ENSG00000161714	1
ENSG00000197943	1
ENSG00000182836	1
ENSG00000124181	1
ENSG00000167004	1
ENSG00000115556	1
ENSG00000154822	1
ENSG00000072818	1
ENSG00000187091	1
ENSG00000139151	1
ENSG00000149782	1
ENSG00000144824	1
ENSG00000114805	1
ENSG00000115896	1
ENSG00000182220	1
ENSG00000113441	1
ENSG00000177674	1
ENSG00000078369	1
ENSG00000153162	1
ENSG00000156113	1
ENSG00000109756	1
ENSG00000164258	1
ENSG00000148730	1
ENSG00000129467	1
ENSG00000157388	1
ENSG00000117602	1
ENSG00000159200	1
ENSG00000172348	1
ENSG00000086730	1
ENSG00000168970	1
ENSG00000204498	1
ENSG00000204496	1
ENSG00000204490	1
ENSG00000204487	1
ENSG00000204475	1
ENSG00000204469	1
ENSG00000175471	1
ENSG00000213658	1
ENSG00000010671	1
ENSG00000161896	1
ENSG00000140563	1
ENSG00000172071	1
ENSG00000173598	1
ENSG00000110931	1
ENSG00000115020	1
ENSG00000101082	1
ENSG00000010610	1
ENSG00000165025	1

ENSG00000163519	**1**
ENSG00000153563	**1**
ENSG00000115085	**1**
ENSG00000179639	**1**
ENSG00000130988	**1**
ENSG00000023516	**1**
ENSG00000111254	**1**
ENSG00000151320	**1**
ENSG00000179841	**1**
ENSG00000105127	**1**
ENSG00000118507	**1**
ENSG00000186009	**1**
ENSG00000129244	**1**
ENSG00000101892	**1**
ENSG00000163399	**1**
ENSG00000069849	**1**
ENSG00000137731	**1**
ENSG00000018625	**1**
ENSG00000132681	**1**
ENSG00000143153	**1**
ENSG00000105409	**1**
ENSG00000047936	**1**
ENSG00000188157	**1**
ENSG00000151466	**1**
ENSG00000091129	**1**
ENSG00000162572	**1**
ENSG00000142185	**1**
ENSG00000111319	**1**
ENSG00000166828	**1**
ENSG00000168447	**1**
ENSG00000110881	**1**
ENSG00000108684	**1**
ENSG00000213199	**1**
ENSG00000072182	**1**
ENSG00000049759	**1**
ENSG00000160183	**1**
ENSG00000101049	**1**
ENSG00000183873	**1**
ENSG00000196876	**1**
ENSG00000148408	**1**
ENSG00000007314	**1**
ENSG00000169432	**1**
ENSG00000136546	**1**
ENSG00000185313	**1**
ENSG00000168356	**1**
ENSG00000118762	**1**
ENSG00000177098	**1**
ENSG00000149575	**1**
ENSG00000166257	**1**
ENSG00000153253	**1**
ENSG00000136531	**1**
ENSG00000144285	**1**
ENSG00000105711	**1**
ENSG00000081248	**1**
ENSG00000126088	**1**
ENSG00000151239	**1**

ENSG00000123349	**1**
ENSG00000177156	**1**
ENSG00000107651	**1**
ENSG00000118515	**1**
ENSG00000136628	**1**
ENSG00000177301	**1**
ENSG00000130427	**1**
ENSG00000106125	**1**
ENSG00000197535	**1**
ENSG00000206305	**1**
ENSG00000206239	**1**
ENSG00000196735	**1**
ENSG00000121281	**1**
ENSG00000138798	**1**
ENSG00000090382	**1**
ENSG00000115204	**1**
ENSG00000113580	**1**
ENSG00000110080	**1**
ENSG00000062485	**1**
ENSG00000198805	**1**
ENSG00000131467	**1**
ENSG00000104324	**1**
ENSG00000004939	**1**
ENSG00000206372	**1**
ENSG00000166278	**1**
ENSG00000204364	**1**
ENSG00000163431	**1**
ENSG00000176340	**1**
ENSG00000186575	**1**
ENSG00000078699	**1**
ENSG00000137869	**1**
ENSG00000172179	**1**
ENSG00000143815	**1**
ENSG00000083444	**1**
ENSG00000215077	**1**
ENSG00000112526	**1**
ENSG00000204256	**1**
ENSG00000131620	**0.5**
ENSG00000100170	**0.5**
ENSG00000080166	**0.5**
ENSG00000173692	**0.5**
ENSG00000175166	**0.5**
ENSG00000140675	**0.5**
ENSG00000131183	**0.5**
ENSG00000151704	**0.5**
ENSG00000080493	**0.5**
ENSG00000164924	**0.5**
ENSG00000143627	**0.5**
ENSG00000108344	**0.5**
ENSG00000126562	**0.5**
ENSG00000197299	**0.5**
ENSG00000081800	**0.5**
ENSG00000196502	**0.5**
ENSG00000165029	**0.5**
ENSG00000127412	**0.5**
ENSG00000091137	**0.5**

ENSG00000165272	**0.5**
ENSG00000145217	**0.5**
ENSG00000081479	**0.5**
ENSG00000197711	**0.5**
ENSG00000107165	**0.5**
ENSG00000118972	**0.5**
ENSG00000001626	**0.5**
ENSG00000171124	**0.5**
ENSG00000074966	**0.5**
ENSG00000133216	**0.5**
ENSG00000160801	**0.5**
ENSG00000112936	**0.5**
ENSG00000164068	**0.5**
ENSG00000102174	**0.5**
ENSG00000183023	**0.5**
ENSG00000017427	**0.5**
ENSG00000005022	**0.5**
ENSG00000111348	**0.5**
ENSG00000196924	**0.5**
ENSG00000122585	**0.5**
ENSG00000110395	**0.5**
ENSG00000211448	**0.5**
ENSG00000079459	**0.5**
ENSG00000177706	**0.5**
ENSG00000142168	**0.5**
ENSG00000167748	**0.5**
ENSG00000149452	**0.5**
ENSG00000047410	**0.5**
ENSG00000213614	**0.5**
ENSG00000044012	**0.5**
ENSG00000049247	**0**
ENSG00000151067	**0**
ENSG00000140199	**0**
ENSG00000120915	**0**
ENSG00000100292	**0**
ENSG00000157168	**0**
ENSG00000197616	**0**
ENSG00000108405	**0**
ENSG00000188170	**0**
ENSG00000159899	**0**
ENSG00000145244	**0**
ENSG00000005187	**0**
ENSG00000164307	**0**
ENSG00000164308	**0**
ENSG00000010322	**0**
ENSG00000139567	**0**
ENSG00000095303	**0**
ENSG00000171564	**0**
ENSG00000171560	**0**
ENSG00000171557	**0**
ENSG00000102032	**0**
ENSG00000100604	**0**
ENSG00000163273	**0**
ENSG00000131979	**0**
