# Supplementary material for: Computational Analysis of Candidate Disease Genes and Variants for Salt-Sensitive Hypertension in Indigenous Southern Africans
Source: PLoS One. 2010 Sep 27;5(9):e12989. doi: 10.1371/journal.pone.0012989 (PMC2946338; doi:10.1371/journal.pone.0012989)
Supplement: Data File S4 — Ensembl IDs, HUGO symbols and descriptions of all top candidates. (0.01 MB PDF) [file pone.0012989.s004.pdf]

Supplementary data S4: Ensembl IDs, HUGO symbols and descriptions of all top candidates

Ensembl ID	HUGO ID	Description
ENSG00000152266	PTH	Parathyroid hormone precursor (Parathyrin) (PTH) (Parathormone). [Source:Uniprot/SWISSPROT;Acc:P01270]
ENSG00000144891	AGTR1	Type-1 angiotensin II receptor (AT1) (AT1AR) (AT1BR). [Source:Uniprot/SWISSPROT;Acc:P30556]
ENSG00000151617	EDNRA	Endothelin-1 receptor precursor (Endothelin A receptor) (ET-A) (hET- AR) (ETA-R). [Source:Uniprot/SWISSPROT;Acc:P25101]
ENSG00000135744	AGT	Angiotensinogen precursor (Serpin A8) [Contains: Angiotensin-1 (Angiotensin I) (Ang I); Angiotensin-2 (Angiotensin II) (Ang II); Angiotensin-3 (Angiotensin III) (Ang III) (Des-Asp[1]-angiotensin II)]. [Source:Uniprot/SWISSPROT;Acc:P01019]
ENSG00000143839	REN	Renin precursor (EC 3.4.23.15) (Angiotensinogenase). [Source:Uniprot/SWISSPROT;Acc:P00797]
ENSG00000107937	GTPBP4	Nucleolar GTP-binding protein 1 (Chronic renal failure gene protein) (GTP-binding protein NGB). [Source:Uniprot/SWISSPROT;Acc:Q9BZE4]
ENSG00000138622	HCN4	Potassium/sodium hyperpolarization-activated cyclic nucleotide-gated channel 4. [Source:Uniprot/SWISSPROT;Acc:Q9Y3Q4]
ENSG00000136160	EDNRB	Endothelin B receptor precursor (ET-B) (Endothelin receptor Non- selective type). [Source:Uniprot/SWISSPROT;Acc:P24530]
ENSG00000099822	HCN2	Potassium/sodium hyperpolarization-activated cyclic nucleotide-gated channel 2 (Brain cyclic nucleotide-gated channel 2) (BCNG-2). [Source:Uniprot/SWISSPROT;Acc:Q9UL51]
ENSG00000214274	ANG	Angiogenin precursor (EC 3.1.27.-) (Ribonuclease 5) (RNase 5). [Source:Uniprot/SWISSPROT;Acc:P03950]
ENSG00000124721	DNAH8	Ciliary dynein heavy chain 8 (Axonemal beta dynein heavy chain 8). [Source:Uniprot/SWISSPROT;Acc:Q96JB1]
ENSG00000175206	NPPA	Atrial natriuretic factor precursor (ANF) (Atrial natriuretic peptide) (ANP) (Prepronatriodilatin) (CDD-ANF) [Contains: Cardiodilatin-related peptide (CDP)]. [Source:Uniprot/SWISSPROT;Acc:P01160]
ENSG00000129965	INS	Insulin precursor [Contains: Insulin B chain; Insulin A chain]. [Source:Uniprot/SWISSPROT;Acc:P01308]
ENSG00000163631	ALB	Serum albumin precursor. [Source:Uniprot/SWISSPROT;Acc:P02768]
ENSG00000137992	DBT	Lipoamide acyltransferase component of branched-chain alpha-keto acid dehydrogenase complex
ENSG00000197956	S100A6	Protein S100-A6 (S100 calcium-binding protein A6) (Calcyclin) (Prolactin receptor-associated protein) (PRA) (Growth factor-inducible protein 2A9) (MLN 4). [Source:Uniprot/SWISSPROT;Acc:P06703]
ENSG00000100359	SGSM3	RUN and TBC1 domain containing 3 [Source:RefSeq_peptide;Acc:NP_056520]
ENSG00000159640	ACE	Angiotensin-converting enzyme
ENSG00000162711	NLRP3	NACHT
ENSG00000078401	EDN1	Endothelin-1 precursor (Preproendothelin-1) (PPET1) [Contains: Endothelin-1 (ET-1); Big endothelin-1]. [Source:Uniprot/SWISSPROT;Acc:P05305]
