# Supplementary material for: Computational Analysis of Candidate Disease Genes and Variants for Salt-Sensitive Hypertension in Indigenous Southern Africans
Source: PLoS One. 2010 Sep 27;5(9):e12989. doi: 10.1371/journal.pone.0012989 (PMC2946338; doi:10.1371/journal.pone.0012989)
Supplement: Data File S5 — Distribution of SNPs with significantly different allele frequencies when comparing Black South African and Caucasian populations. Each black cross represents a SNP with allele frequencies that differ at the indicated significance, between Caucasian and South African populations. Grey crosses indicate all SNPs assayed. Gene name, chromosome and position are shown along the horizontal axis; Gene exons are shown as vertical black bars. (1.13 MB PDF) [file pone.0012989.s005.pdf]

Supplementary data File S-5: Distribution of SNPs with significantly different allele frequencies when comparing Black South African and Caucasian populations. Each black cross represents a SNP with allele frequencies that differ at the indicated significance, between Caucasian and South African populations. Grey crosses indicate all SNPs assayed. Gene name, chromosome and position are shown along the horizontal axis; Gene exons are shown as vertical black bars.

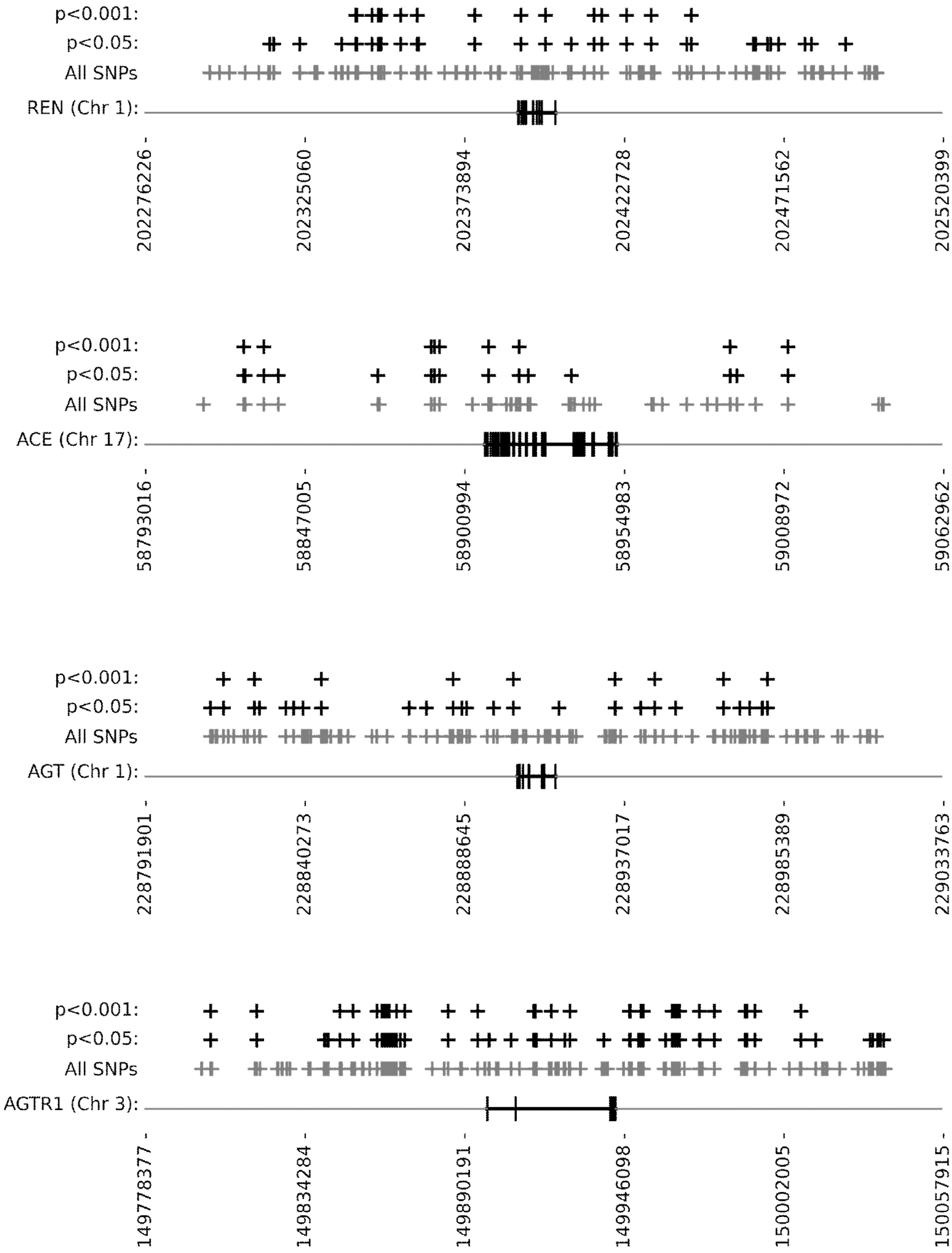

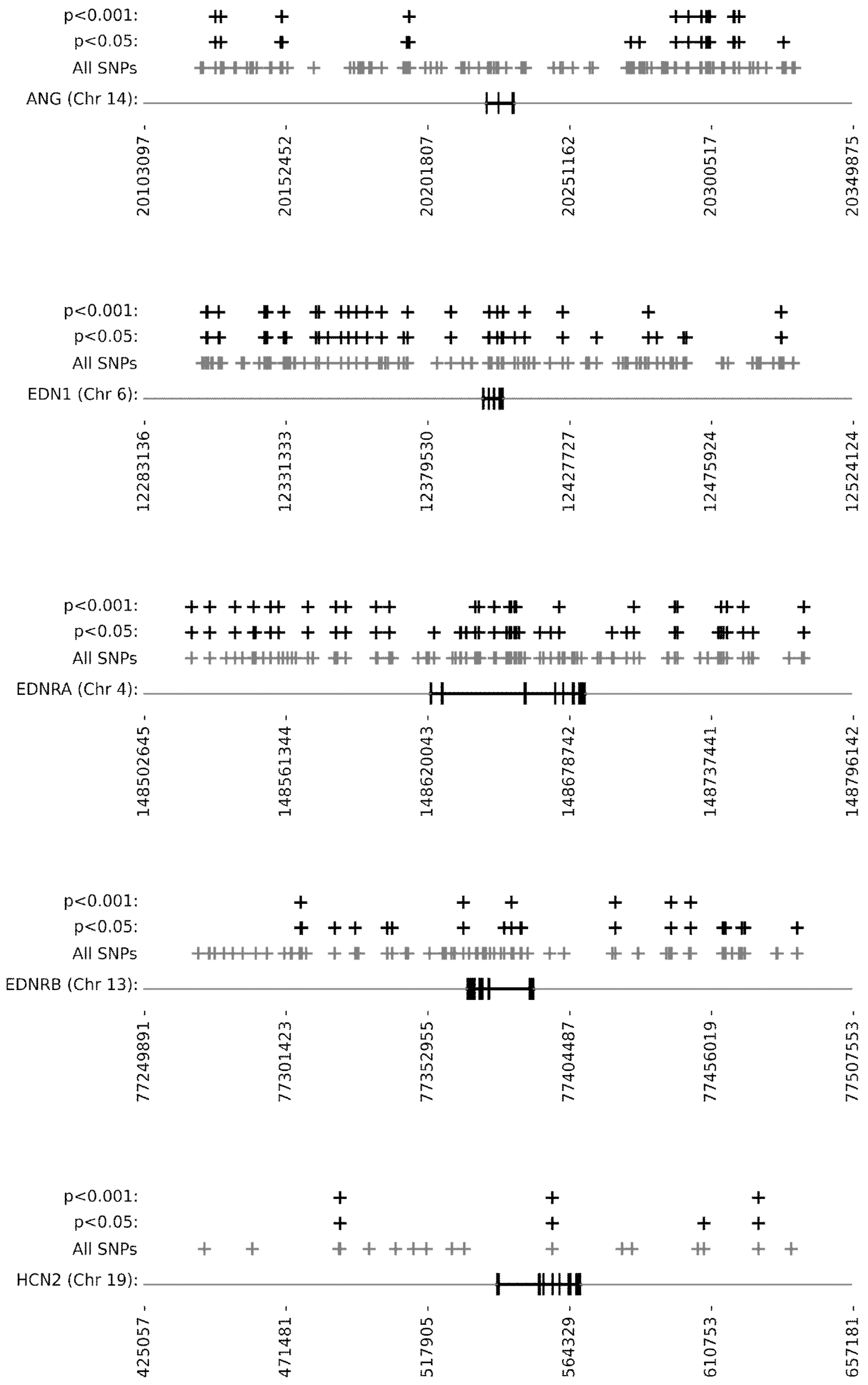
p<0.001:
p<0.05:
All SNPs
ANG (Chr 14):
20103097
20152452
20201807
20251162
20300517
20349875
p<0.001:
p<0.05:
All SNPs
EDN1 (Chr 6):
12283136
12331333
12379530
12427727
12475924
12524124
p<0.001:
p<0.05:
All SNPs
EDNRA (Chr 4):
148502645
148561344
148620043
148678742
148737441
148796142
p<0.001:
p<0.05:
All SNPs
EDNRB (Chr 13):
77249891
77301423
77352955
77404487
77456019
77507553
p<0.001:
p<0.05:
All SNPs
HCN2 (Chr 19):
425057
471481
517905
564329
610753
657181

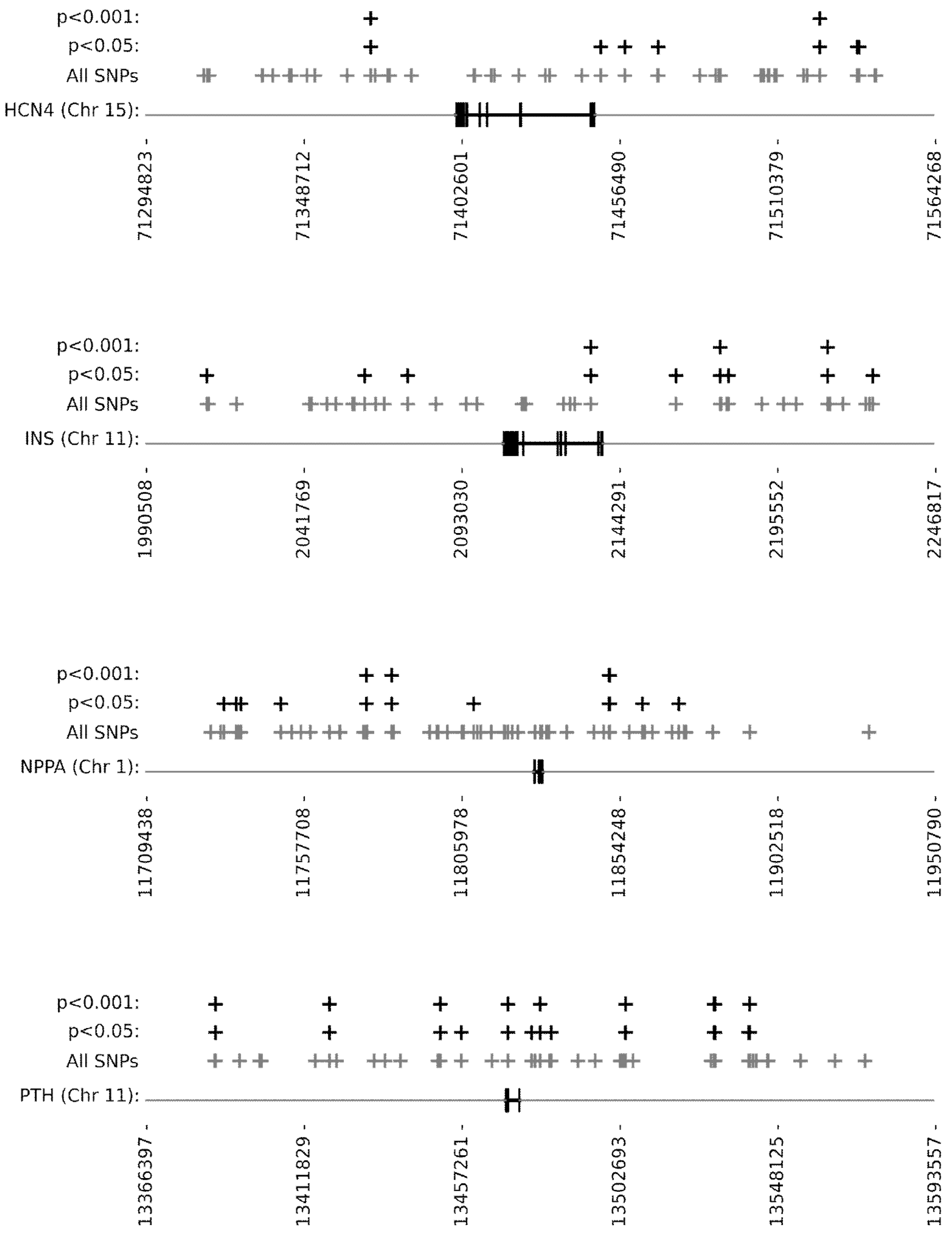
p<0.001:
p<0.05:
All SNPs
HCN4 (Chr 15):
71294823
71348712
71402601
71456490
71510379
71564268
p<0.001:
p<0.05:
All SNPs
INS (Chr 11):
1990508
2041769
2093030
2144291
2195552
2246817
p<0.001:
p<0.05:
All SNPs
NPPA (Chr 1):
11709438
11757708
11805978
11854248
11902518
11950790
p<0.001:
p<0.05:
All SNPs
PTH (Chr 11):
13366397
13411829
13457261
13502693
13548125
13593557
