# Supplementary material for: Computational Analysis of Candidate Disease Genes and Variants for Salt-Sensitive Hypertension in Indigenous Southern Africans
Source: PLoS One. 2010 Sep 27;5(9):e12989. doi: 10.1371/journal.pone.0012989 (PMC2946338; doi:10.1371/journal.pone.0012989)
Supplement: Data File S6 — Heatmaps showing copy number variation analysis for candidate genes. Red shading shows a deletion at that position, blue shading shows a copy number gain at that position. HUGO gene symbol is shown. Chrm = chromosome. Green lines denote the gene start and end. Horizontal axis shows position on the chromosome. Vertical axis shows the individuals from each population: CEU = HapMap Central European population, XHS = Xhosa population, HER = Hererro population, STS = Setswana population, ZUL = Zulu population, KHS = Khoisan population. (3.53 MB PDF) [file pone.0012989.s006.pdf]

Supplementary Data File S-6: Heatmaps showing copy number variation analysis for candidate genes. Red shading shows a deletion at that position, blue shading shows a copy number gain at that position. HUGO gene symbol is shown. Chrm = chromosome. Green lines denote the gene start and end. Horizontal axis shows position on the chromosome. Vertical axis shows the individuals from each population: CEU = HapMap Central European population, XHS = Xhosa population, HER = Hererro population, STS = Setswana population, ZUL = Zulu population, KHS = Khoisan population.

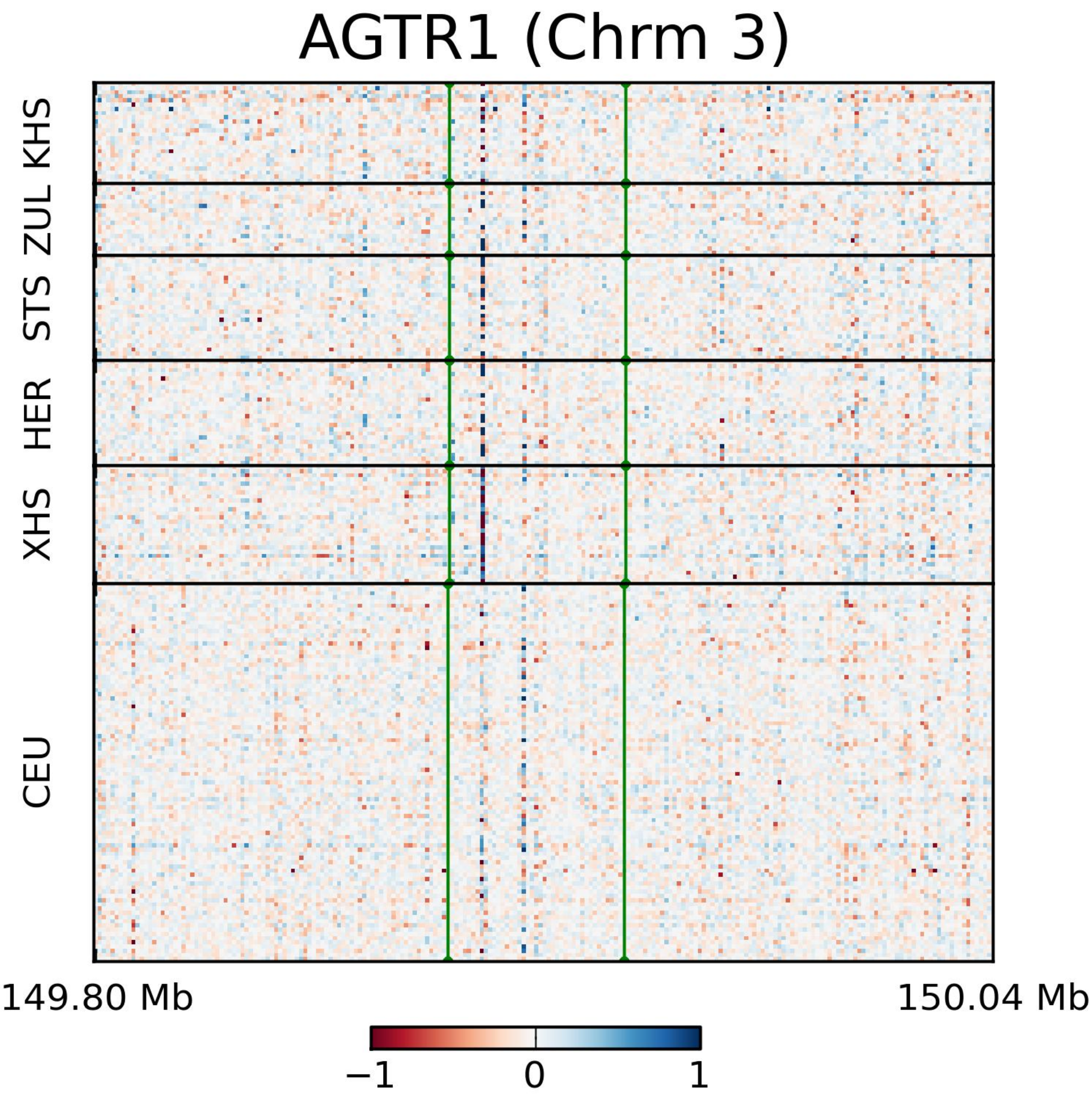

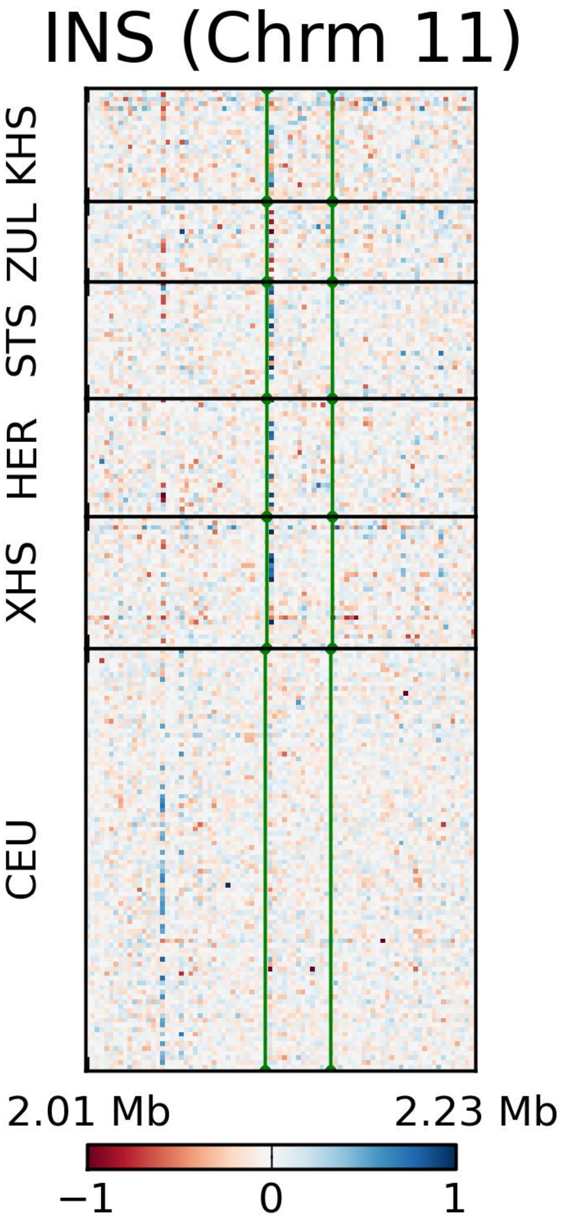
INS (Chrm 11)
KHS
ZUL
STS
HER
XHS
CEU
2.01 Mb
2.23 Mb
−1
0
1

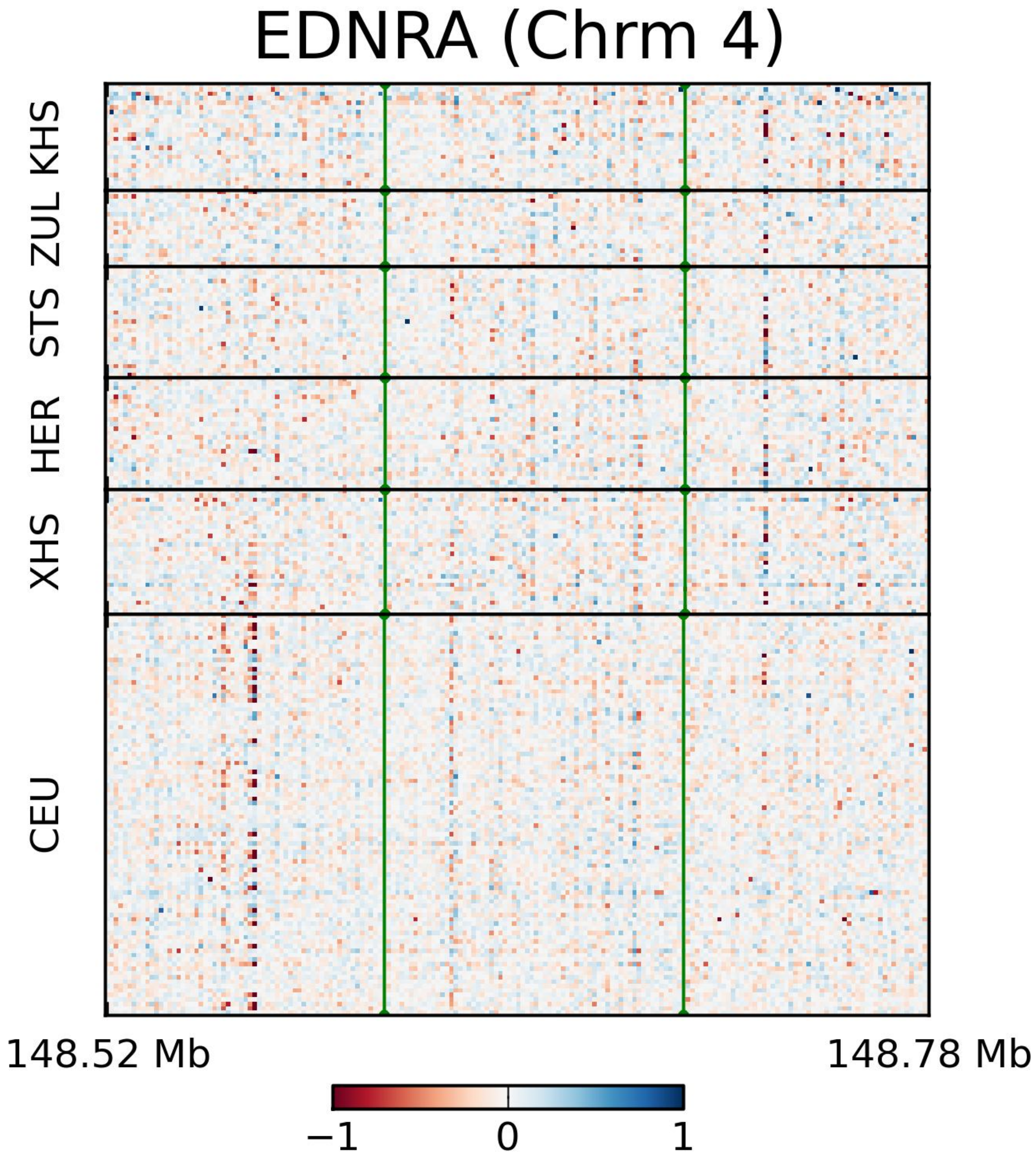
EDNRA (Chrm 4)
KHS
ZUL
STS
HER
XHS
CEU
148.52 Mb
148.78 Mb
−1
0
1

EDNRB (Chrm 13)

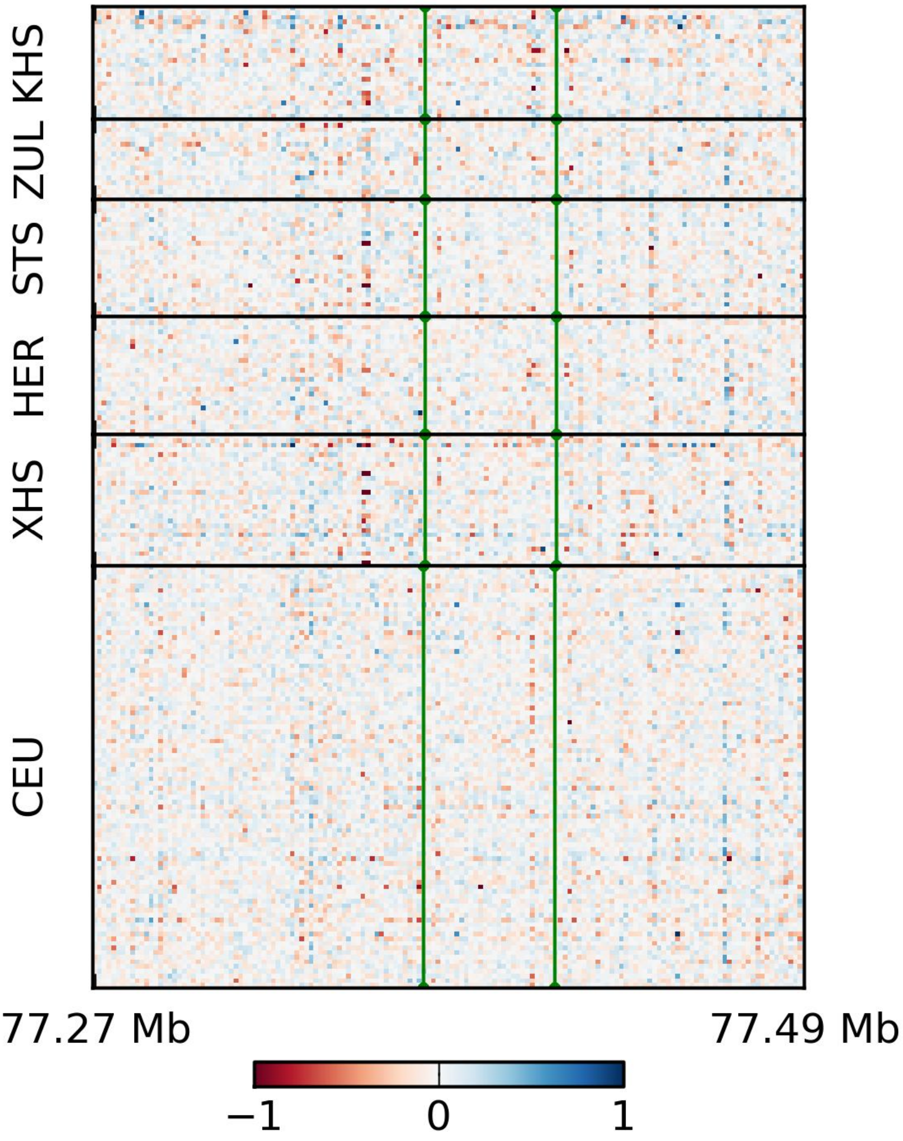

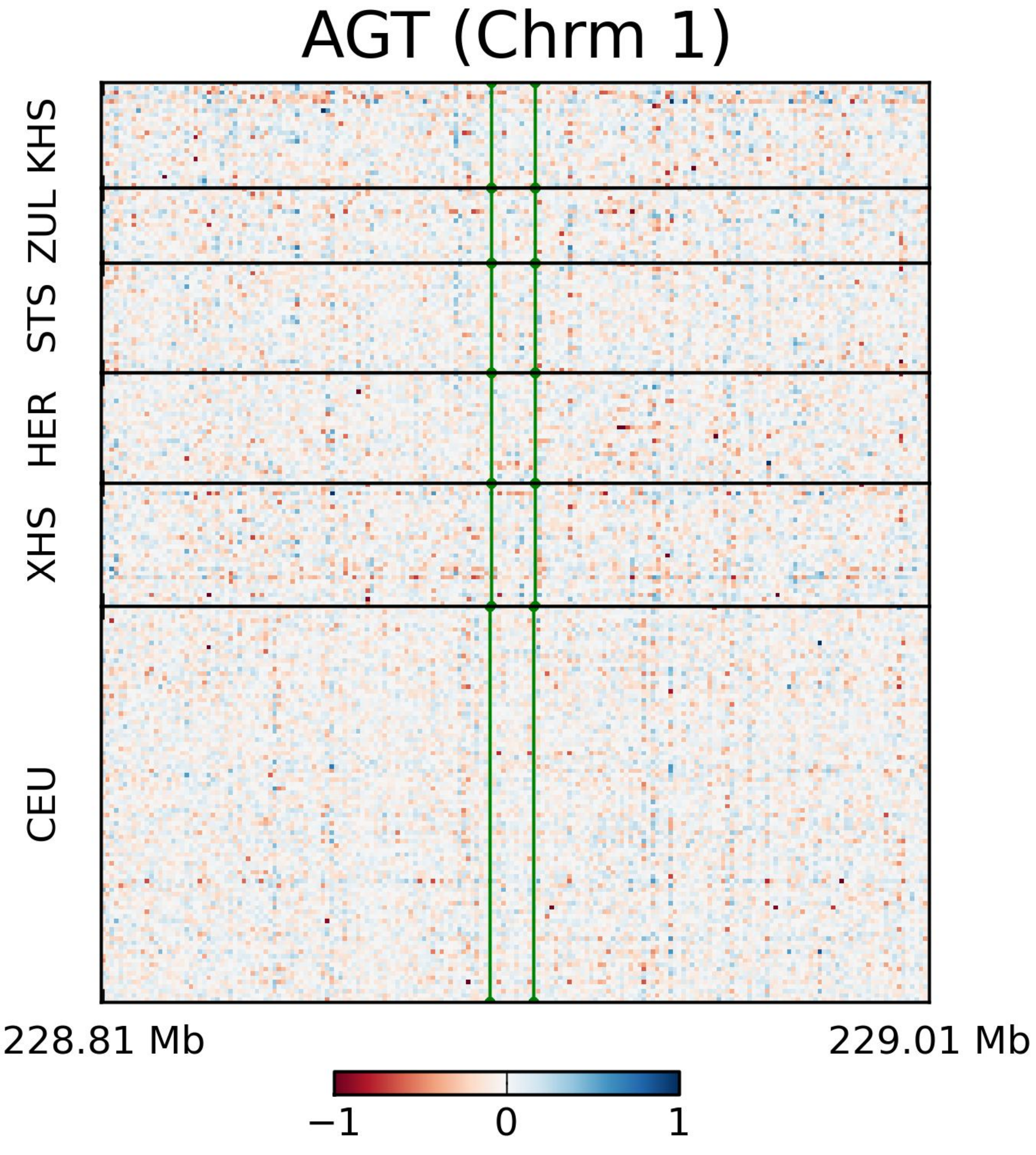
AGT (Chrm 1)
KHS
ZUL
STS
HER
XHS
CEU
228.81 Mb
229.01 Mb
−1
0
1

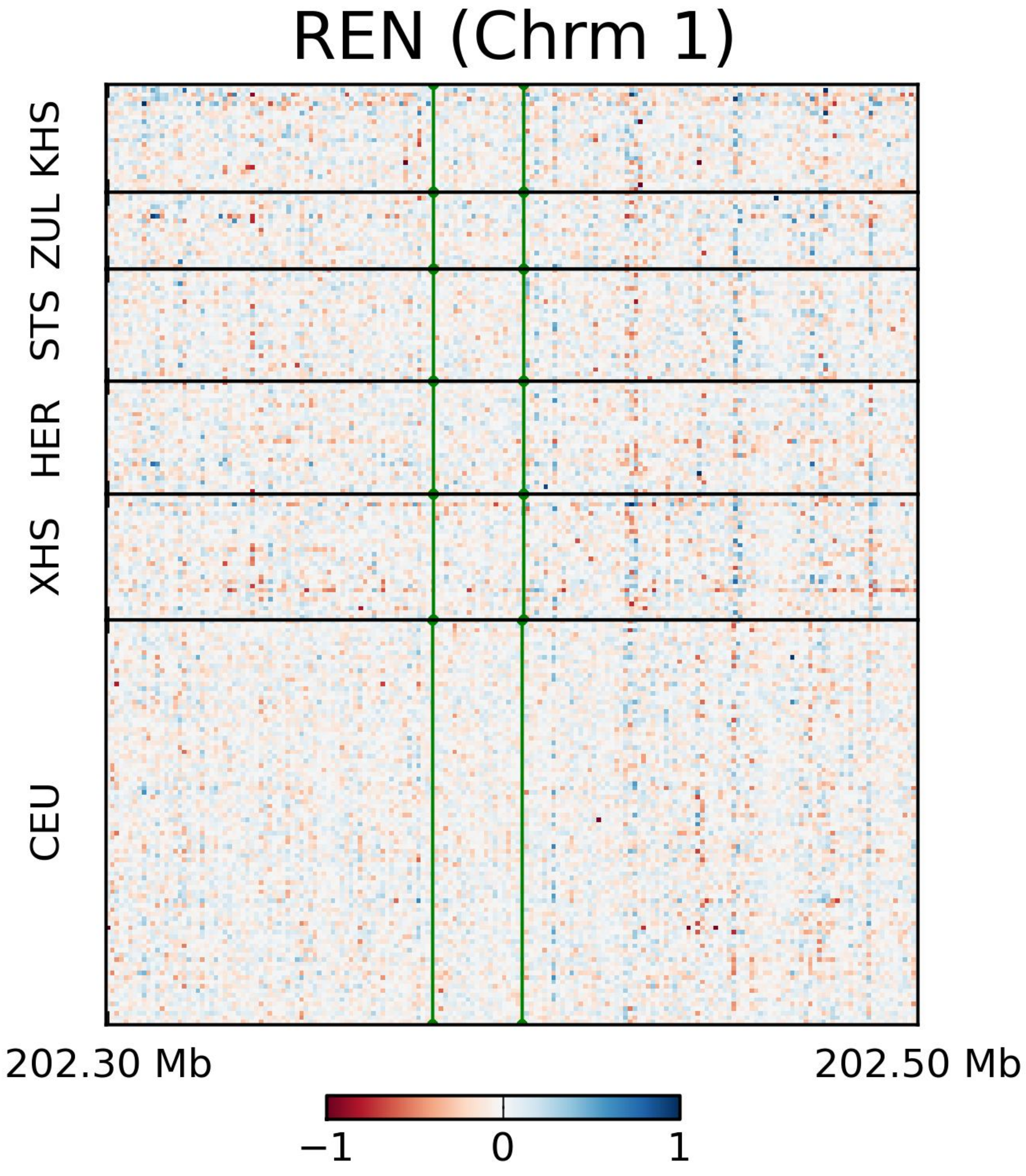
REN (Chrm 1)
KHS
ZUL
STS
HER
XHS
CEU
202.30 Mb
202.50 Mb
−1
0
1

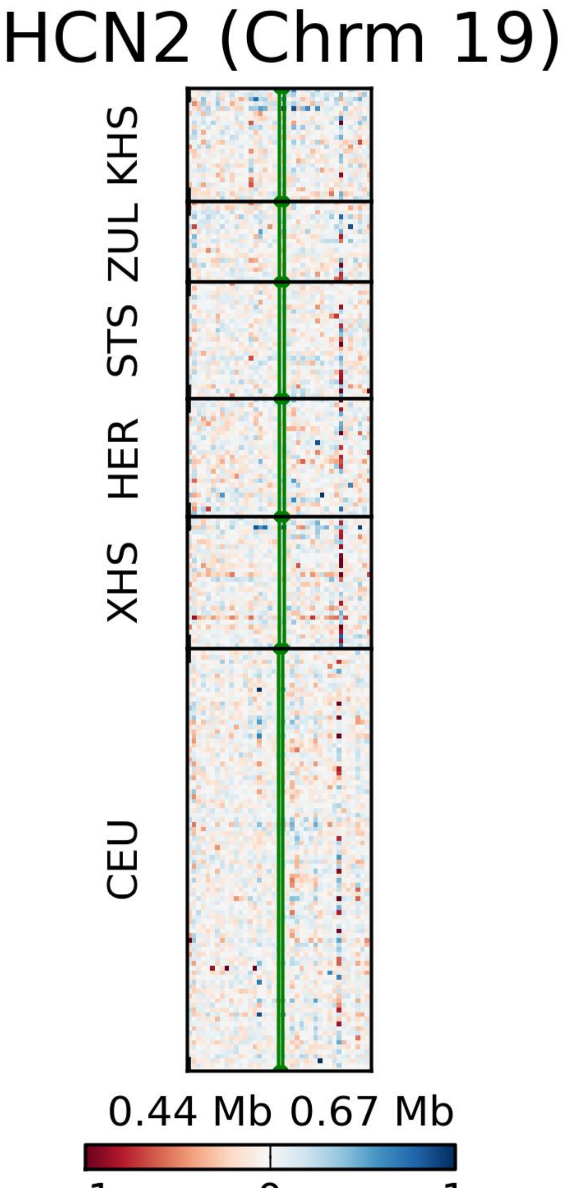
HCN2 (Chrm 19)
KHS
ZUL
STS
HER
XHS
CEU
0.44 Mb
0.67 Mb
−1
0
1

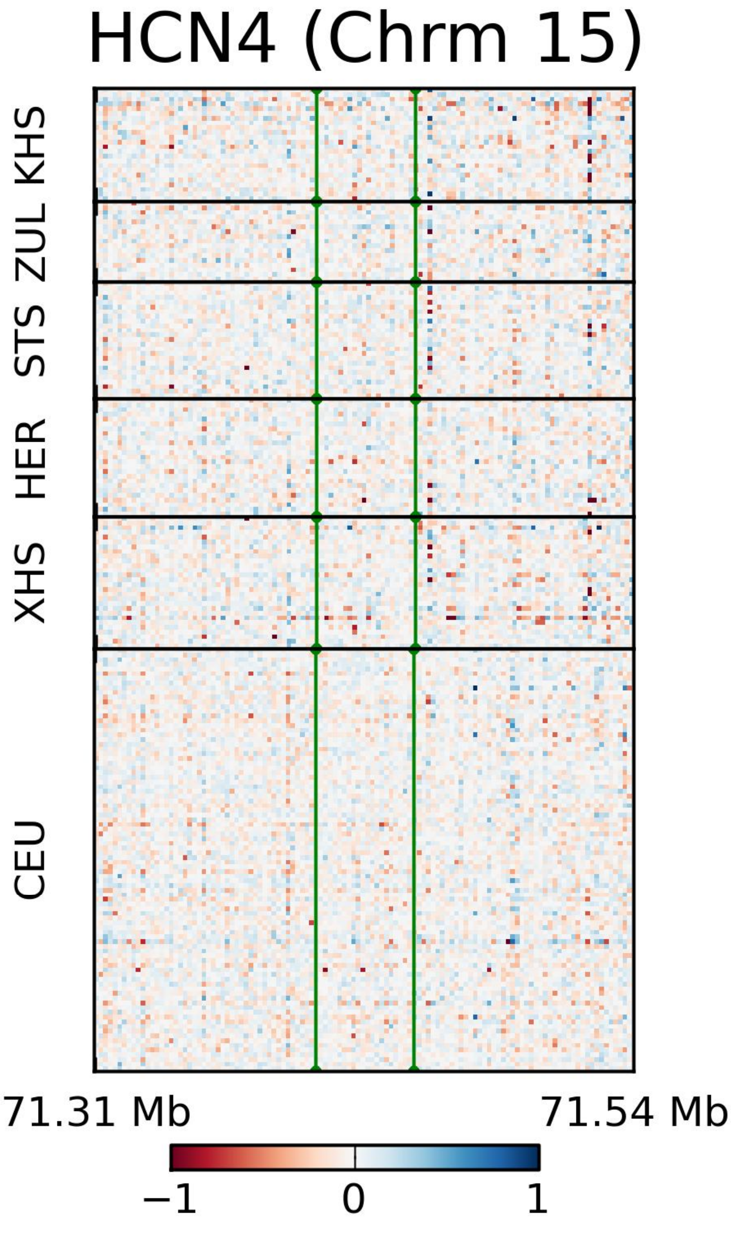
HCN4 (Chrm 15)
KHS
ZUL
STS
HER
XHS
CEU
71.31 Mb
71.54 Mb
−1
0
1

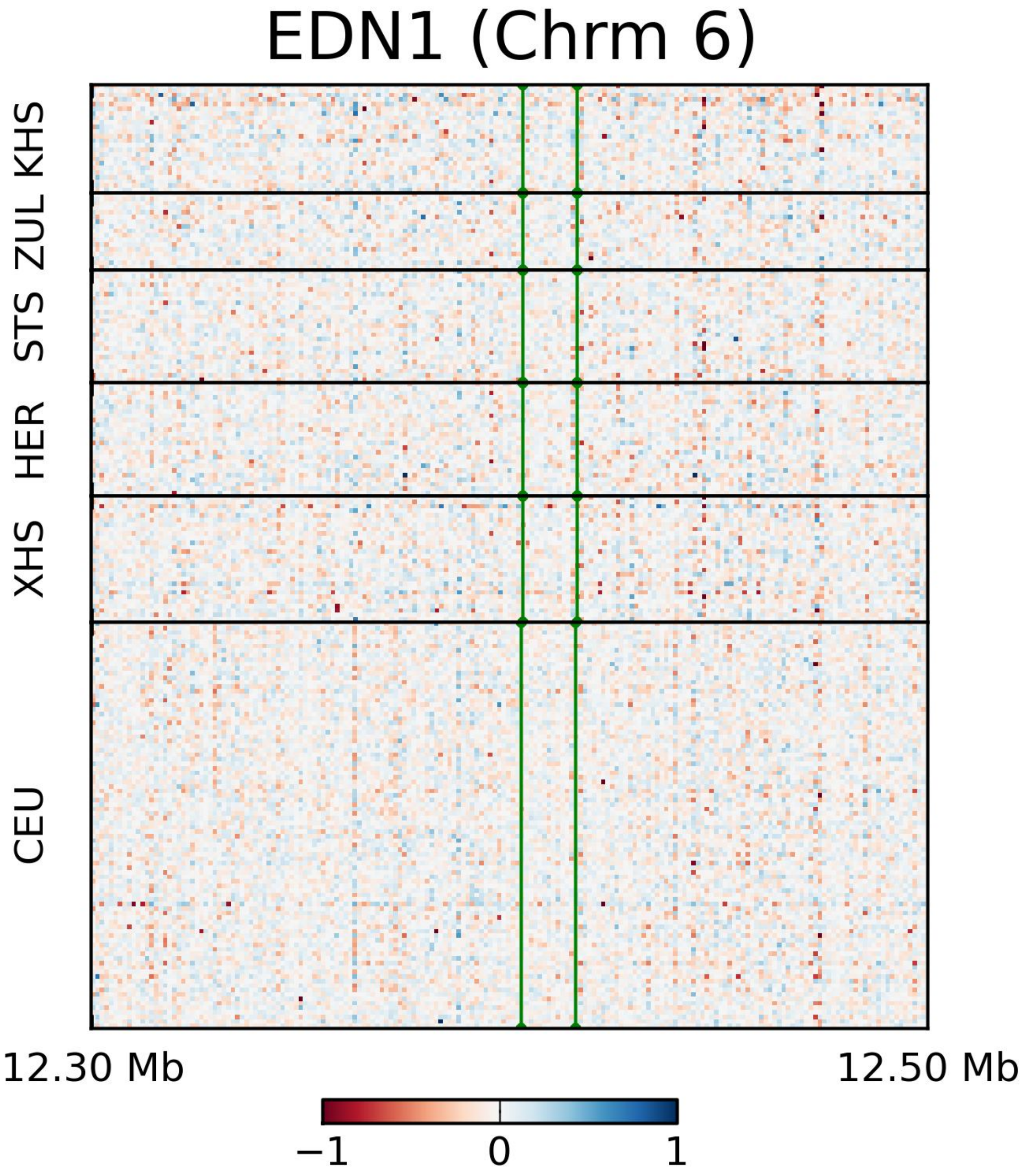
EDN1 (Chrm 6)
KHS
ZUL
STS
HER
XHS
CEU
12.30 Mb
12.50 Mb
−1
0
1

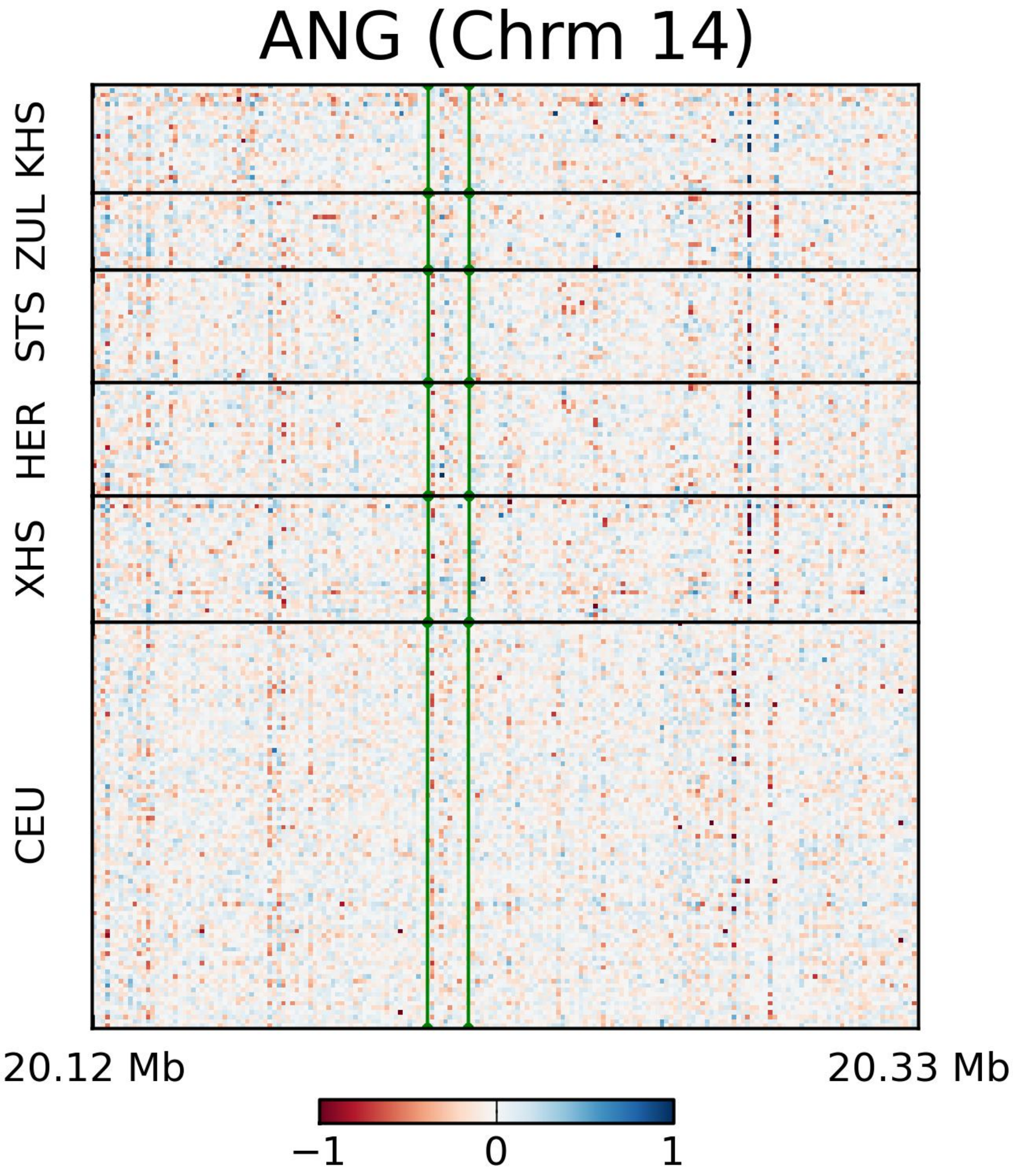
ANG (Chrm 14)
KHS
ZUL
STS
HER
XHS
CEU
20.12 Mb
20.33 Mb
−1
0
1

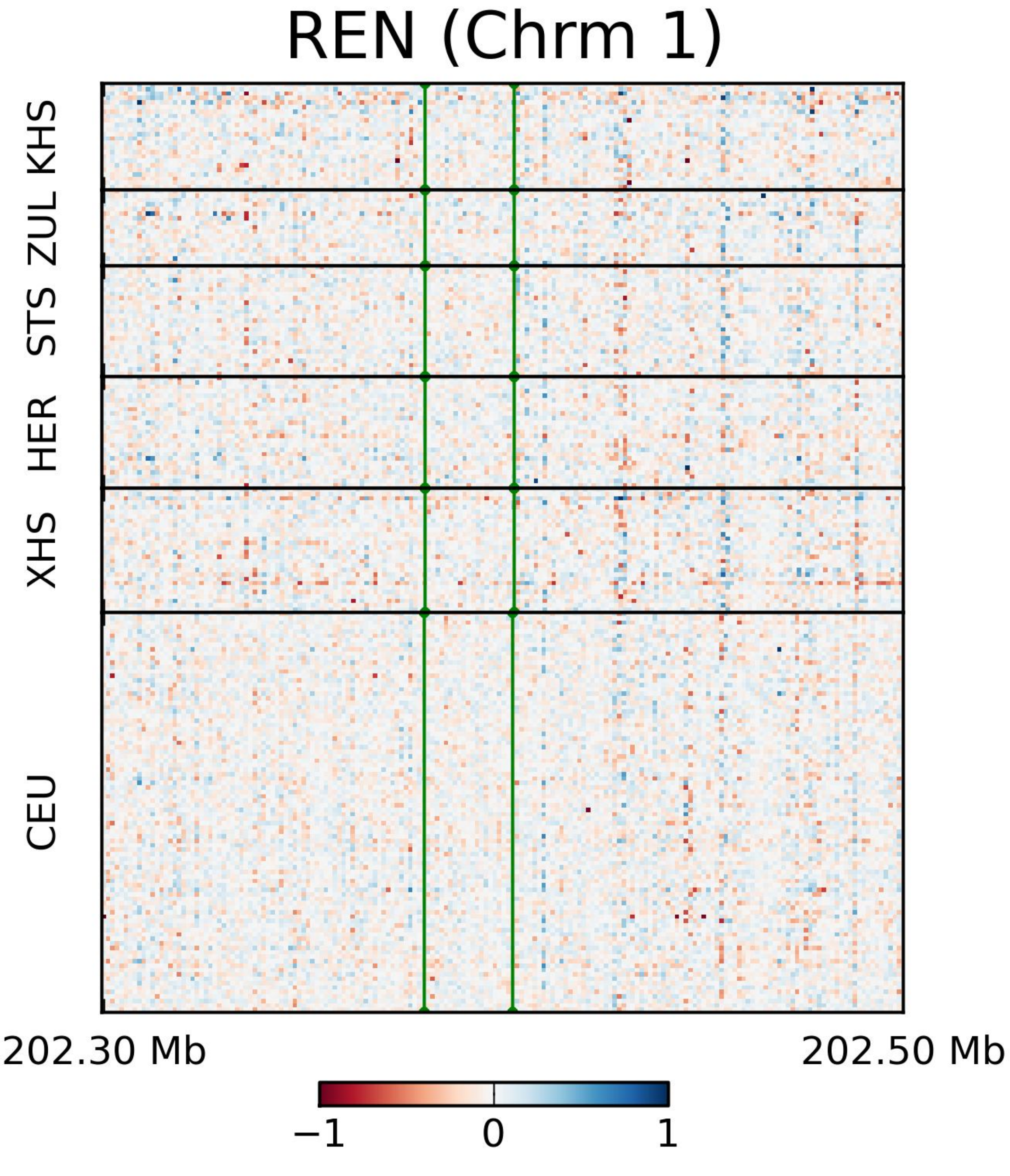
REN (Chrm 1)
KHS
ZUL
STS
HER
XHS
CEU
202.30 Mb
202.50 Mb
−1
0
1

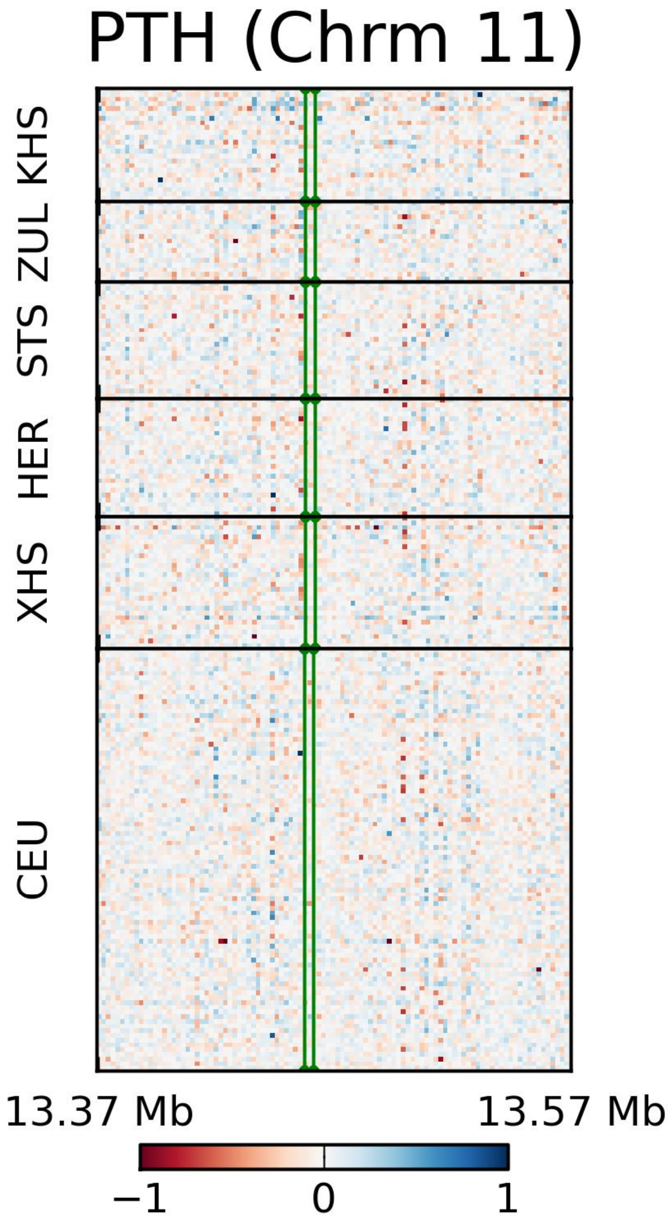

PTH (Chrm 11)
KHS
ZUL
STS
HER
XHS
CEU
13.37 Mb
13.57 Mb
−1
0
1

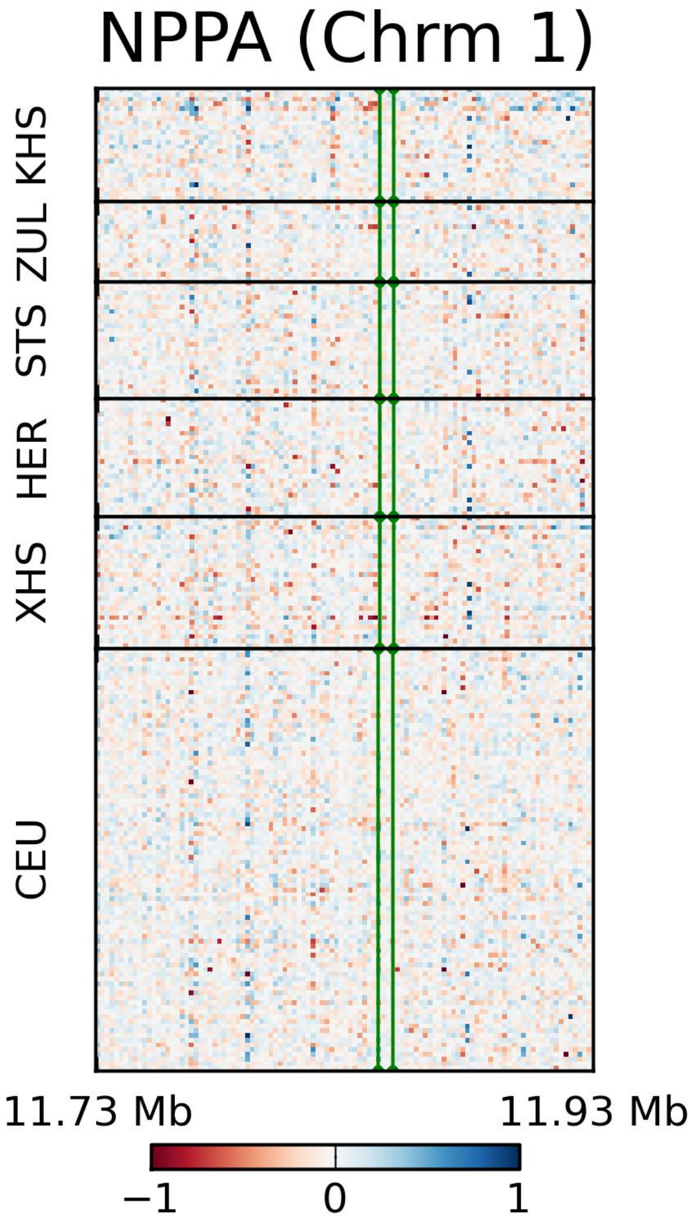
NPPA (Chrm 1)
KHS
ZUL
STS
HER
XHS
CEU
11.73 Mb
11.93 Mb
−1
0
1
